# Equipping Coiled-Coil Peptide Dimers With Furan Warheads Reveals Novel Cross-Link Partners

**DOI:** 10.3389/fchem.2021.799706

**Published:** 2022-02-16

**Authors:** Laia Miret-Casals, Sander Van De Putte, Dorien Aerssens, Julien Diharce, Pascal Bonnet, Annemieke Madder

**Affiliations:** ^1^ Department of Organic and Macromolecular Chemistry, Organic and Biomimetic Chemistry Research Group, Faculty of Science, Ghent University, Ghent, Belgium; ^2^ Institut de Chimie Organique et Analytique, Université d’Orléans, UMR CNRS 7311, Orléans, France

**Keywords:** furan oxidation, singlet oxygen, molecular modeling, cross-link (CL), coiled-coil peptide, protein–protein interaction (PPI), peptide–protein interaction

## Abstract

Using a coiled-coil peptide dimer as a model system to explore furan reactivity, we describe novel cross-link partners of furan warheads for site-specific cross-linking. We demonstrate that replacement of weak interhelical ionic contacts with a furan moiety and its potential cross-link partner affords covalently connected coiled-coil motifs upon furan activation. We describe for the first time the reaction of the activated furan warhead with cysteine and tyrosine, besides the previously reported lysine, thus enhancing the versatility of the furan cross-link methodology by the possibility to target different amino acids. The present *in vitro* validation of “furan-armed” *α*-helices provides further grounds for exploiting furan technology in the development of furan-modified ligands/proteins to target proteins in a covalent way through various amino acid side chains.

## Introduction

**FIGURE 1:**
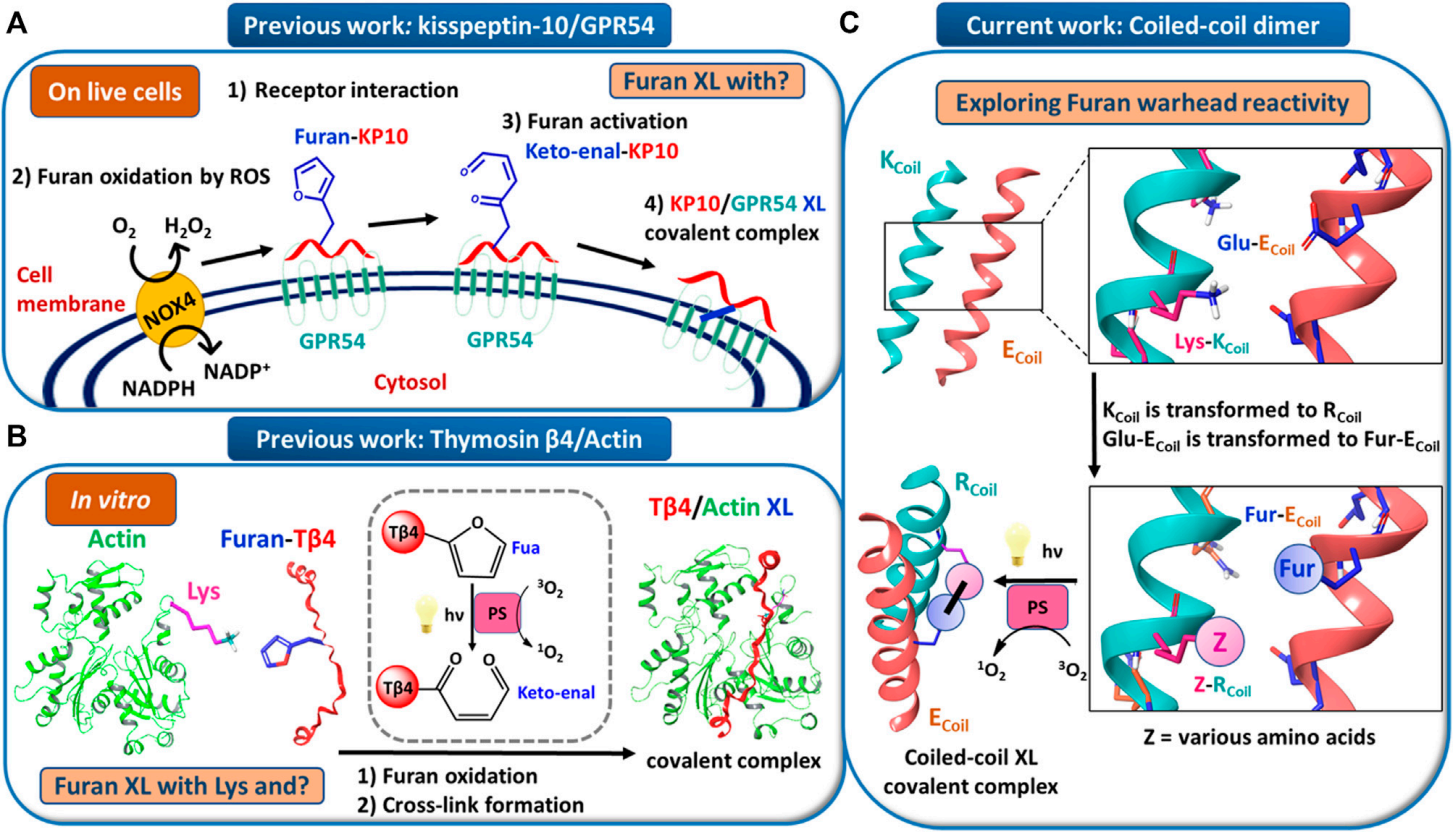
**(A)** Model for endogenous furan oxidation by reactive oxygen species (ROS). The mature glycosylated GPR54 receptor is present at the cell surface. When the furan-modified kisspeptin-10 peptide (Furan-KP10) is added, it interacts at the cell surface with GPR54 (step 1). Due to the presence of an NADPH oxidase (likely NOX4), present in the plasma membrane, ROS are locally released in the extracellular environment. Then, the Furan-KP10 is oxidized by ROS (step 2) to a keto-enal-KP10 (step 3) and forms a covalent cross-link with the receptor (step 4). **(B)** Model for *in vitro* furan oxidation by singlet oxygen production. Actin and Fua-T*β*4 were first pre-incubated. Then, the furan moiety was activated by oxidation followed by cross-link formation. The oxidation relies on singlet oxygen generation by visible light irradiation in the presence of a photosensitizer (PS), such as Rose Bengal or Rhodamine B. **(C)** Model system to explore furan warhead reactivity towards different amino acid side chains in a coiled-coil peptide heterodimer.

**FIGURE 2:**
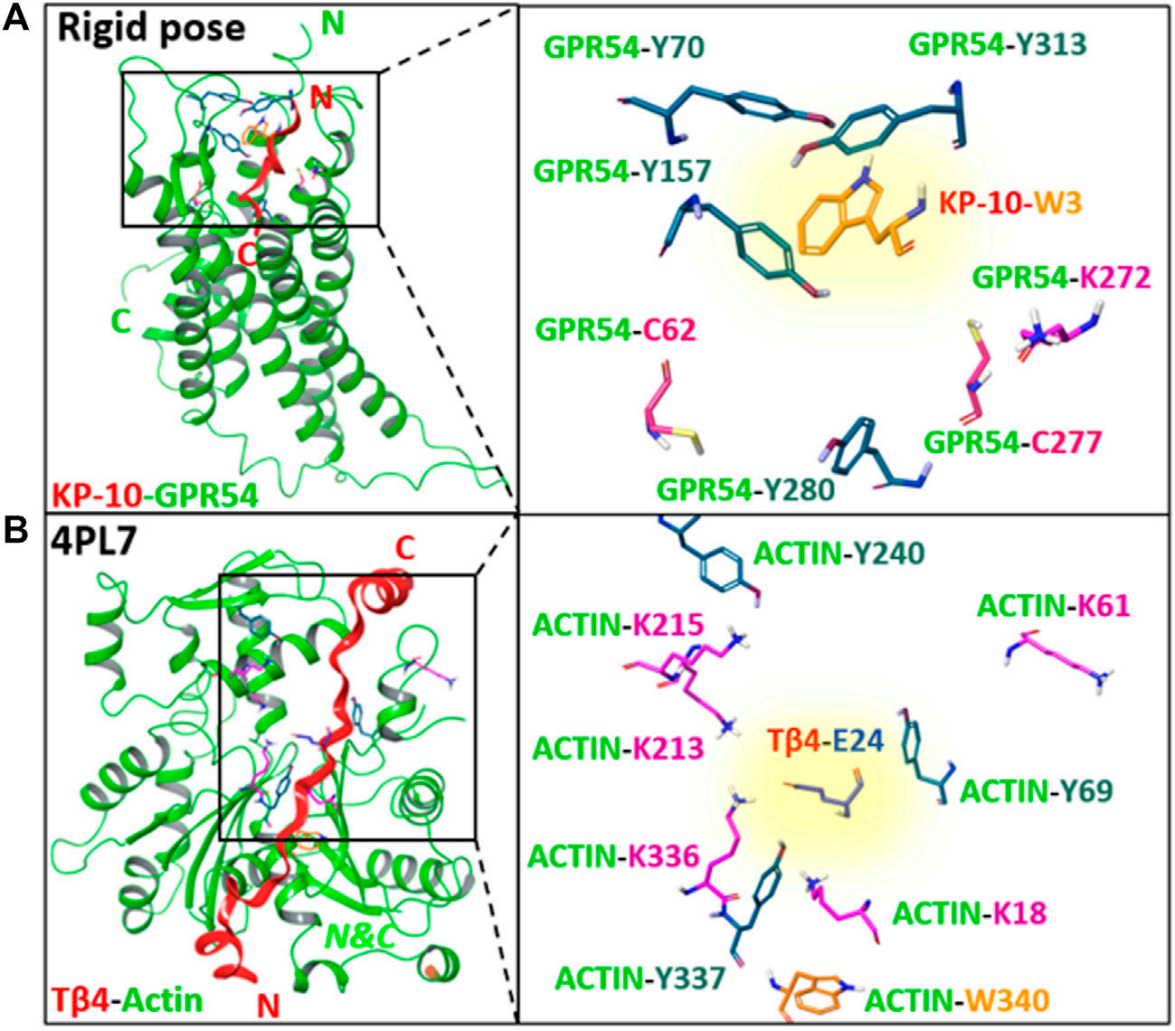
**(A)** The best predicted docking pose of the native kisspeptin-10 ligand into the GPR54 model receptor structure using the rigid pose conformation of the receptor. **(B)** 3D structure of T*β*4-actin complex based on PDB 4PL7. Left panels: an overview of the complex system; right panels: zoom in on GPR54/ACTIN amino acids side chains (Lys, Cys, Tyr, and Trp) proximate to KP-10-**W3** or T*β*4-**E24**, respectively, without the secondary structure. The amino acid that is replaced by furylalanine (W3 in kisspeptin, E24 in Thymosin *β*4) is highlighted with a yellow background. N, C = N-, C-termini*.* Images prepared using MAESTRO (for details, see ESI 1).

Peptide–protein and protein–protein interactions play key roles in biological processes. Therefore, approaches to target proteins in a covalent way can provide useful analytical tools for chemical biology and could lead to potential applications in therapeutic development. We recently reported on a novel cross-link technology applied to peptide–protein (Vannecke et al., 2017) and protein–protein (Miret-Casals et al., 2021a) interactions, based on the introduction of an unnatural, furan-containing amino acid in a peptide or protein and its subsequent oxidation into an electrophilic moiety upon the action of reactive oxygen species (ROS) or visible light irradiation in the presence of a photosensitizer (PS, Figures 1A,B, respectively) (Vannecke et al., 2017; Miret-Casals et al., 2021a). Initially, a furan modified kisspeptin-10 peptide ligand could be covalently cross-linked to its membrane receptor, GPR54, on live cancer cells with spontaneous oxidation of the furan moiety by endogenous ROS (Figure 1A) (Vannecke et al., 2017). The GPR54 (also called KISS1R) is a well-known modulator of the physiology of reproduction in mammals (Pinilla et al., 2012). GPR54 binds kisspeptins, a family of truncated forms of kisspeptin peptides (54, 14, 13, or 10 amino acids) with a common Arg-Phe-amide C terminus responsible for the high-affinity binding and activation of GPR54 (Kotani et al., 2001). We chemically synthesized kisspeptin-10 (KP-10) replacing the Trp3 residue with a furylalanine (Fua) amino acid. We could demonstrate that upon incubation of live cells expressing the GPR54 receptor with the furan-modified peptide, a covalently cross-linked kisspeptin-10/GPR54 complex was formed as demonstrated by extensive analysis of the cell lysate by Western blotting (Vannecke et al., 2017). Due to the complexity of the system combined with the low abundance of the GPR54 membrane receptor, we were not able to determine the identity of the attacking amino acid residue and the exact structure of the cross-linked species, despite extensive attempts at receptor pulldown and MS-based identification of the complex. In order to shed more light on the exact nature of the nucleophile involved in the covalent bond formation, and since no crystal structure of GPR54 is available, a homology model was built (see ESI 1 and Supplementary Figure S1). Three GPCRs were used as templates: the nociceptin opioid receptor, the delta-opioid receptor, and the kappa-opioid receptor. The homology model of GPR54 was built using Modeller software (Webb and Sali, 2016) and 10 models were generated. Molecular dynamics were applied with Amber 16 (Case, 2016) to relax the generated structure and one representative structure was selected to propose two starting points for the prediction of the GPR54/kisspeptin-10 interaction: the rigid and the relaxed state of GPR54. Then, the new *in silico* Iterative Residue Docking and Linking method (Diharce et al., 2019), which allows to efficiently predict peptide–protein interactions, was used to dock kisspeptin-10. The best predicted docking poses of the native kisspeptin-10 ligand into the GPR54 model receptor structure using the rigid (Figure 2A; Supplementary Figure S2A in ESI 1) and the relaxed (Supplementary Figure S2B in ESI 1) conformation of the receptor show that several GPR54-tyrosine (Y) residues, as well as GPR54-tryptophan (W), -cysteine (C), and -lysine (K) are located in sufficient proximity of the KP-10-W3 residue and could cross-link when W3 is replaced by Fua. In a more recent work on the further use of the furan-oxidation strategy for protein–protein cross-linking, we studied the interaction between actin, the major cytoskeletal protein of the cell that forms filaments, and T*β*4, which regulates the polymerization of actin and keeps it in the monomeric form. Starting from the available 3D structure–function information on the T*β*4–actin complex (PDB 4PL7, Figure 2B), we determined the optimal position to insert a furan in T*β*4 (T*β*4-E24), and several Actin-lysine (K) residues were identified as potential nucleophiles, proximate enough to engage in covalent bond formation (Miret-Casals et al., 2021a). In addition, several Actin-tyrosine (Y) residues were also observed in close proximity to T*β*4-E24 (Figure 2B). The furan-modified T*β*4 analogue was found able to efficiently cross-link to monomeric actin upon singlet oxygen generation by irradiation in the presence of a PS (Figure 1B), and the cross-link site in the T*β*4-actin covalent complex was characterized in detail (Miret-Casals et al., 2021a). In this case, we could firmly establish that lysine is a target for the furan warhead, and a potential chemical structure of the covalent adduct between lysine and the activated furan was put forward based on the MS data (Miret-Casals et al., 2021a). Even if these cross-linked residues were identified by MS, it cannot be excluded that other amino acids in Actin can also react but the cross-linked products resulting thereof were not detected due to a lack of knowledge on all potential furan-cross-link partners. In both studies, the furan moiety is used as a caged warhead and can be triggered to a keto-enal (electrophilic intermediate) by the production of singlet oxygen (Figures 1A,B). Earlier studies on the toxicity of furan derivatives resulting from furan metabolism *in vivo* describe both amine and thiol species as suitable target nucleophiles for activated furan (Peterson, 2013).

In order to further explore the scope and limitations of furan-oxidation-based cross-linking to proteins, we aimed at determining which amino acid other than lysine can be potentially targeted by a furan warhead and lead to a stable cross-linked adduct. For that purpose, we searched for a system able to mimic protein–protein interactions and suited for rational protein design. Coiled-coil systems are described by the interaction between two or more *α*-helices that coil around each other to form a supercoiled structure composed of identical or different helices, resulting in homomers or heteromers that can be arranged in a parallel or antiparallel manner (Lupas, 1996; Litowski and Hodges, 2002; Mason and Arndt, 2004; Liu et al., 2006; Grigoryan and Keating, 2008; Boyle and Woolfson, 2011; Woolfson et al., 2017; Lapenta et al., 2018).

**FIGURE 3:**
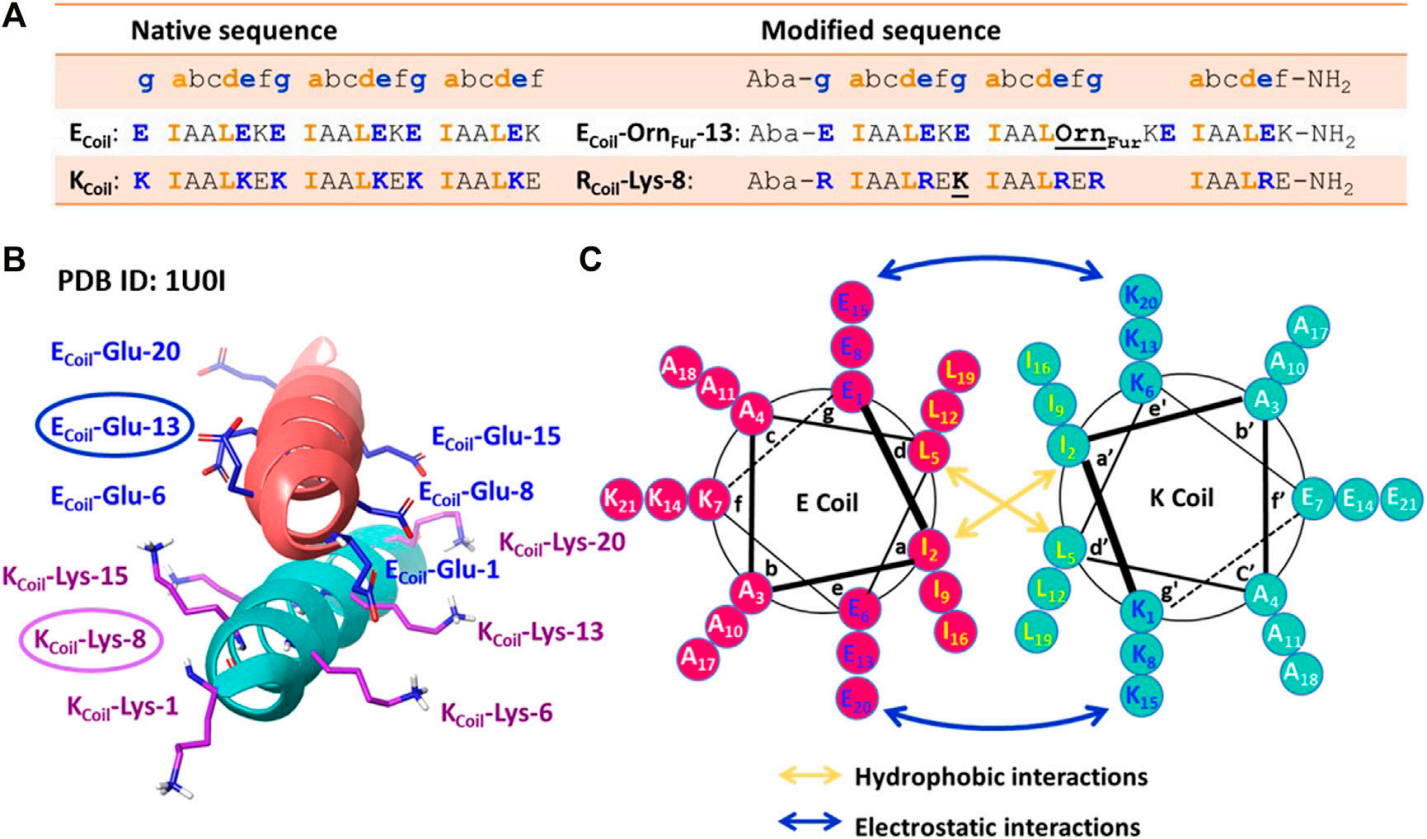
**(A)** Peptide sequences of the native [(EIAALEK)3/(KIAALKE)3] and modified coiled-coil complex. Peptide sequences are written N-terminus to C-terminus. The four amino acids (IAAL) in the peptide sequence correlate with the positions a, b, c, and d of the coiled-coil heptad repeat (abcdefg), and EKE (ECoil) or KEK/RER (K/RCoil) correlate with the e, f, and g positions. An Aba (aminobenzoic acid) group is introduced at the N-terminus and NH2 indicates the amide group at the C-terminus. **(B)** Crystal structure of the (EIAALEK)3/(KIAALKE)3 coiled-coil heterodimer in which the partner moieties of interest (Ecoil: Glu at position 13 and Kcoil: Lys at position 8) are indicated. PDB ID: 1U0I. **(C)** Helical wheel representation of the (EIAALEK)3/(KIAALKE)3 coiled-coil heterodimer viewed in cross-section. The interhelical hydrophobic interactions are denoted with orange arrows, and interhelical electrostatic interactions are denoted by the blue arrows.

Coiled-coil helical systems represent ideal scaffolds to bring potential peptide cross-link partners in close proximity and we decided to evaluate the propensity for site-specific cross-linking between two coils, one of which is equipped with a furan moiety (Figure 1C). In this work, the well-known heterodimeric E3/K3 coiled-coil (Litowski and Hodges, 2002) was used as a model system (Figure 1C), which is composed of a total of 42 residues (Figure 3A), comprising two complementary right-handed *α*-helices wrapped around each other in a left-handed fashion (Figure 3B and the helical wheel diagram displayed in Figure 3C). Each coil peptide contains three heptad repeats (3 × *gabcdef*) of seven amino acids with hydrophobic contacts at the a and d positions, ionic residues at the e and g positions, and generally polar residues at the surface-exposed positions b, c, and/or f to enhance solubility (Figure 3A). The residues at the e and g positions are crucial for ensuring either homo or hetero association in native coiled-coils (Lavigne et al., 1995; Kohn et al., 1998; Krylov et al., 1994), and this has been key to design heterodimeric coiled-coils (Zhou et al., 1994). The E3/K3 coiled-coil with the sequence (**E**IAAL**E**K)_3_/(**K**IAAL**K**E)_3_ contains a high number of glutamic acids (Glu, **E**) and lysines (Lys, **K**) at positions e and g to be complementary to one another, allowing 10 electrostatic interaction pairs at the interface between the two subunits to enhance heterodimer stability and destabilize homodimer formation (Litowski and Hodges, 2002) (Figure 3). Folding a sequence with this repeating pattern into an alpha-helical secondary structure causes the hydrophobic residues (isoleucine and leucine at positions a and d, respectively) to be presented in a way that allows interaction with the same residues of the complementary coil (Figure 3C). This characteristic “knobs-into-holes” manner of packing (Litowski and Hodges, 2002) establishes the hydrophobic core. The stability of such coiled-coils is directly proportional to the correct complementarity of both the electrostatic and hydrophobic interactions at the interface, resulting in a high-affinity binding with a dissociation constant of 70 nM (Litowski and Hodges, 2002). In the current work, we apply the furan technology for the first time in this coiled-coil peptide heterodimer as a model system to explore the reactivity of oxidized furan moieties towards different amino acid side chains (Figure 1C).

## Results and Discussion

### Coiled-Coil Structure and Design Rationale for the Current Study

Based on the published NMR structure of the E3/K3 coiled-coil (PBD 1U01) (Lindhout et al., 2004), we determined the optimal position to insert a furan-containing amino acid and the potential nucleophiles on the E3/K3_Coil_, respectively. In the first instance, we tried to reproduce the previously observed cross-linking [*vide supra* (Miret-Casals et al., 2021a)] between an activated furan moiety and a lysine residue located in close proximity in the current coiled-coil context. We envisioned that the replacement of a weak interhelical ionic bond (E_Coil_: Glu at position 13 and K_Coil_: Lys at position 8, Figure 3B) respectively with the two partner moieties able to engage in covalent bond formation would not interfere with the stability of the coiled-coil structure. The salt-bridging pairs with the shortest distances (Lindhout et al., 2004), essential for the coiled-coil formation, and the Glu and Lys residues positioned at the N/C terminus with higher flexibility were left untouched. To allow selective cross-linking and avoid problems with the formation of multiple adducts, the K_Coil_ was transformed to an R_Coil_ (Figure 1C), where all lysines, except for the Lys residue at position 8, were replaced by arginine (resulting in the sequence R_Coil_-Lys-8), which allows preserving the same number of positive charges in the K_Coil_ but eliminates potential competing nucleophiles (Figure 3A). Introduction of the furan moiety for cross-linking involves the precursor unnatural amino acid Orn (ornithine), which is coupled with 2-furanpropionic acid (Fur) through the amino group of the Orn side chain placed at position 13 on the E_Coil_ (Figure 3A, resulting in the sequence E_Coil_-Orn_Fur_-13). The ornithine residue, featuring three methylene spacers between the backbone and the amine attachment point for the furan moiety, was chosen to give enough flexibility to the Fur moiety to scan its R_Coil_ proximate surroundings for potential furan-cross-link partners. In addition, the peptides were additionally modified with an Aba (aminobenzoic acid) group on the N-terminus to increase the UV activity for HPLC detection and with an amide group on the C-terminus to minimize any repulsive electrostatic interactions between the E3/R3 coiled-coil (Figure 3A).

### The E3/R3 Coiled-Coil System is an ideal Model to Explore Furan Reactivity

To ensure that the chosen coiled-coil model effectively allows cross-linking of the furan warhead with lysine, we thus chemically synthesized E_Coil_-Orn_Fur_-13 and R_Coil_-Lys-8. We assumed that the modified coiled-coil peptides will have similar K_D_ as the native E3/K3 coiled-coil (Figure 3A, ESI 2.1-2.7, and ESI 3.2-3.3). Initially, both coils were pre-incubated to allow coiled-coil formation. At a concentration of 10 μM for each coil peptide, the monomer↔dimer equilibrium is shifted toward the formation of a coiled-coil dimer in 92.0% (see ESI 2.10). Then, the furan moiety can be selectively oxidized to a reactive keto-enal upon generation of singlet oxygen by light irradiation in the presence of a photosensitizer, such as Rose Bengal (RB) or Rhodamine B (Rhd B) (Miret-Casals et al., 2021a) (see ESI 2.8 and 2.9). A site-selective cross-link reaction can then occur between the oxidized furan moiety at the E_Coil_ and the amine group present in the R_Coil_ if proximate.

**FIGURE 4:**
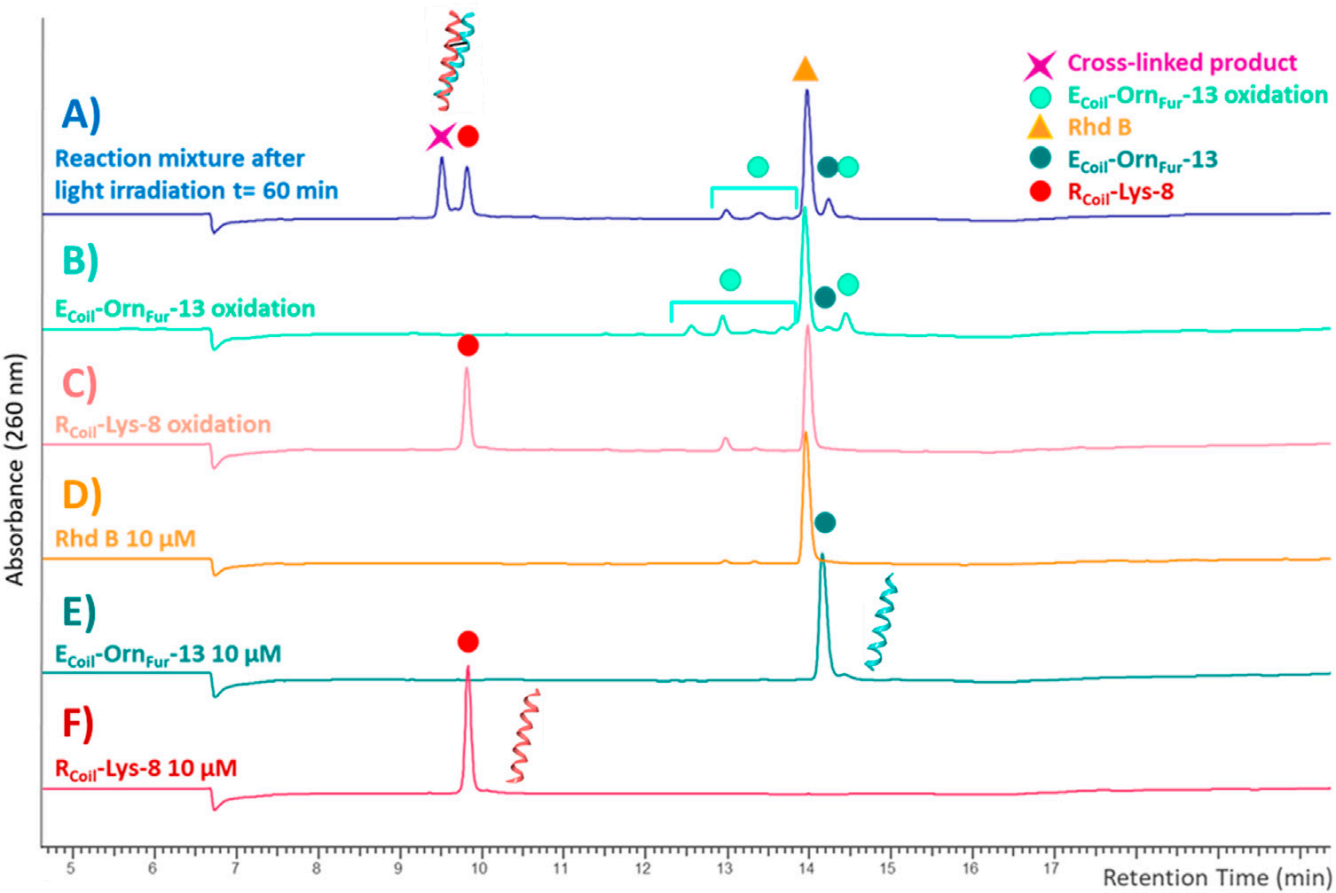
HPLC-UV chromatograms recorded at 260 nm with a XTerra® Shield RP18 column, 125 Å (5 μM 2.1 × 250 mm). The reaction mixture after light irradiation [blue trace **(A)**] is the cross-link reaction between R_Coil_-Lys-8 [red trace **(F)**] and E_Coil_-Orn_Fur_-13 [dark green trace **(E)**] after 60 min of light irradiation with Rhd B [orange trace **(D)**] at 10 μM. The R_Coil_-Lys-8 oxidation (light red) trace **(C)** and E_Coil_-Orn_Fur_-13 oxidation (green) trace **(B)** were generated by exposure to singlet oxygen by light irradiation in the presence of Rhd B at 10 μM for 60 min in absence of the other coil.

**FIGURE 5:**
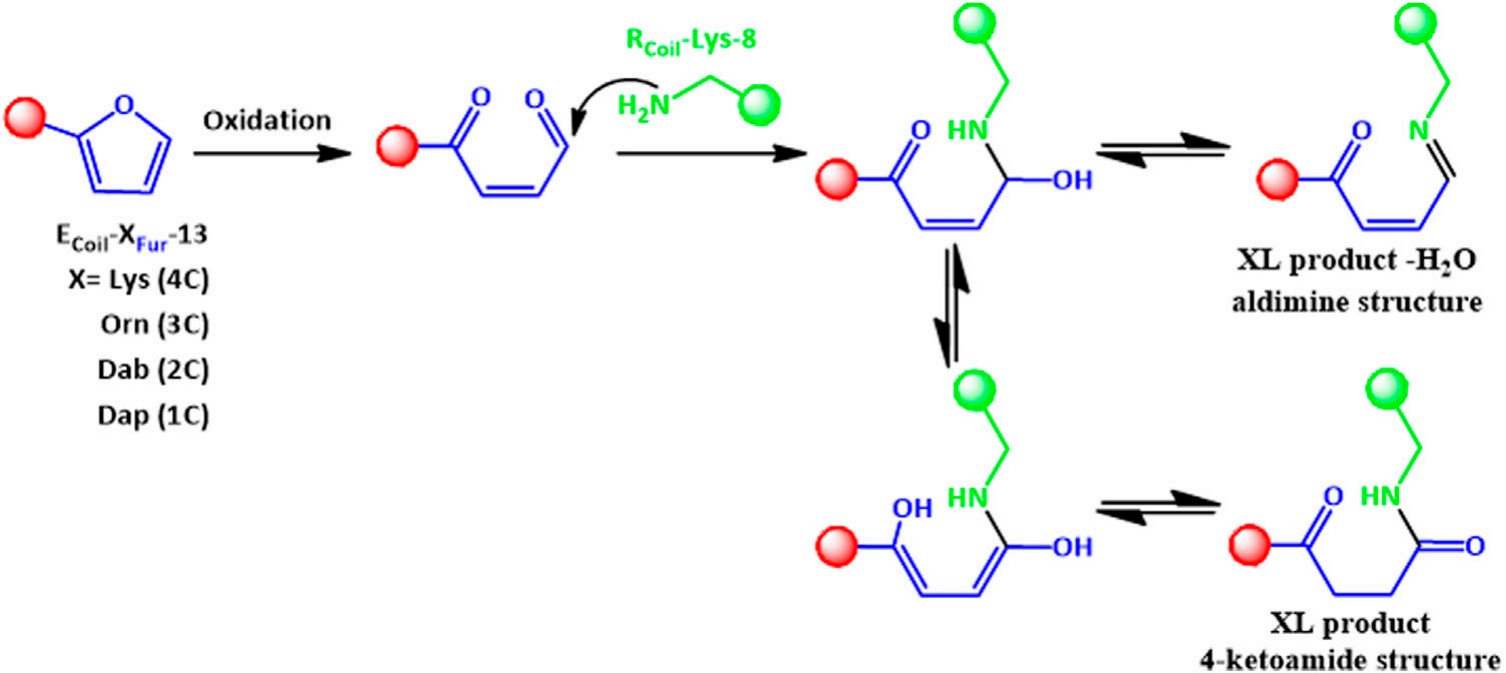
The 4-ketoamide structure is proposed as the end product of the reaction between E_Coil_-X_Fur_-13 with R_Coil_-Lys-8 after furan activation, with the same exact mass as the structure described previously in Miret-Casals et al. (2021b). An aldimine species (XL product—H_2_O) can also be formed. XL refers to cross-linked species.

**FIGURE 6:**
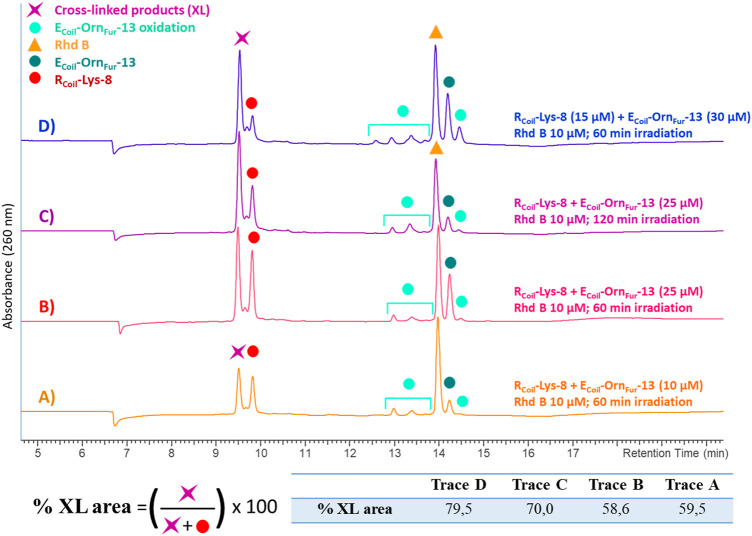
All the chromatograms show the cross-link reaction product (purple star) between R_Coil_-Lys-8 (red dot) and E_Coil_-Orn_Fur_-13 (dark green dot) after light irradiation with Rhd B (orange triangle) at 10 μM. The E_Coil_-Orn_Fur_-13 oxidation products (light green dots) are produced after activation of the furan moiety by singlet oxygen generation. **(A)** R_Coil_-Lys-8 (10 μM) and E_Coil_-Orn_Fur_-13 (10 μM) and 60 min of light irradiation. **(B)** R_Coil_-Lys-8 (25 μM) and E_Coil_-Orn_Fur_-13 (25 μM) and 60 min of light irradiation. **(C)** R_Coil_-Lys-8 (25 μM) and E_Coil_-Orn_Fur_-13 (25 μM) and 120 min of light irradiation. **(D)** R_Coil_-Lys-8 (15 μM) and E_Coil_-Orn_Fur_-13 (30 μM) and 60 min of light irradiation. The cross-link yield was quantified as the percentage of the cross-linked product area and the values are indicated in the table.

The reactivity of the oxidized furan moiety towards nucleophiles is followed by HPLC analysis of the corresponding reaction mixtures (Figure 4) after light irradiation (trace A) of R_Coil_-Lys-8 (trace F) and E_Coil_-Orn_Fur_-13 (trace E) and concurrent singlet oxygen production with Rhd B (D trace). The individual peptides were also exposed to singlet oxygen using the same conditions but in absence of the complementary coil (traces C and B, respectively). Note that the cross-linked product peak is generated (purple star in trace A) and the area of the signal corresponding to the R_Coil_-Lys-8 (red dot in trace A) has decreased compared to the red dot in trace C. Importantly, R_Coil_-Lys-8 does cross-link to activated E_Coil_-Orn_Fur_-13 using RB; however, different cross-linked products were observed (see ESI 4.1, Supplementary Figure S14). The Rhd B-irradiation protocol (10 μM; 60 min of light irradiation, Figure 4) generates one cross-linked product with the expected mass of the covalent product previously described in Miret-Casals et al. (2021a) and the dehydrated form (−18 Da, see ESI 4.2, Supplementary Figure S15). The oxidized covalent product (+16 Da) is also detected and becomes the main product when applying the RB-irradiation protocol (2.5 μM; 30 min of light irradiation, see ESI 4.1, Supplementary Figure S15 in comparison with Supplementary Figure S16 in 4.2). We have previously described that singlet oxygen produced by PS-irradiation protocols can produce oxidation-induced damage in several amino acids (such as Cys, Met, Tyr, His, and Trp) (Miret-Casals et al., 2021a). However, the E_Coil_-Orn_Fur_-13/R_Coil_-Lys-8 coiled-coil model system does not contain amino acids prone to oxidation. The fact that we observe an oxidized cross-link product in the current system thus indicates that the oxidation takes place at the level of the newly formed covalent adduct. Earlier work from Zhu and Sayre (2007) describes a 4-ketoamide as the end product of the reaction of a 4-oxo-2-enal species with a primary amine in pH 7.4 HEPES buffer. Such a 4-ketoamide structure results in the same exact mass as the structure described in Miret-Casals et al. (2021a) (Figure 5, XL product). The oxidized cross-linked product (+16 Da) can be explained by oxidation of the 4-ketoamide by oxygen-derived free radical reactions due to singlet oxygen production. In addition, R_Coil_-Lys-8 can react with the keto-enal-E_Coil_ forming an aldimine (cross-link product −18 Da, Figure 5). To further increase the level of cross-linking (ensuring more complete furan-activation) while minimizing oxidation of the cross-linked product, several conditions were tested using the Rhd B-irradiation protocol [i.e., time dependency of ^1^O_2_ production, dose dependency of the coiled-coil interaction, and E_Coil_:R_Coil_ ratio (Figure 6)]. To allow a more quantitative analysis of the cross-link yield and to compare between different experiments, the percentage of the cross-linked product was calculated integrating the different signals in the HPLC chromatograms taking into account that the ratio between photosensitizer concentration and coil peptide concentration is changing, as well as the E/R_Coil_ ratio (see ESI 5.1-5.2 for a more detailed explanation of how the respective yields were calculated). The concentration of both coils was increased from 10 to 25 μM (see Figures 6A,B, respectively) to study the influence of the coiled-coil interaction on the cross-link yield, but no differences were observed in terms of the cross-linked product area as indicated in the table of Figure 6. Note that cross-link efficiency at 10–25 μM is independent of concentration because there is almost complete association of monomers to the coiled-coil dimer (see ESI 2.10). Next, the ^1^O_2_ production time was increased up to 120 min, and the cross-link efficiency improved from 59% to 70% (Figure 6C). The highest level of cross-linked product (79.5%) was achieved when changing the E_Coil_:R_Coil_ ratio from 1:1 to 2:1 (Figures 6B,D, respectively); however, single and double oxidized covalent products (+16, +32 Da, respectively, which probably relate to free radical oxidation products of the 4-ketoamide) were also detected (Supplementary Figures S19–S20 in 5.4).

**FIGURE 7:**
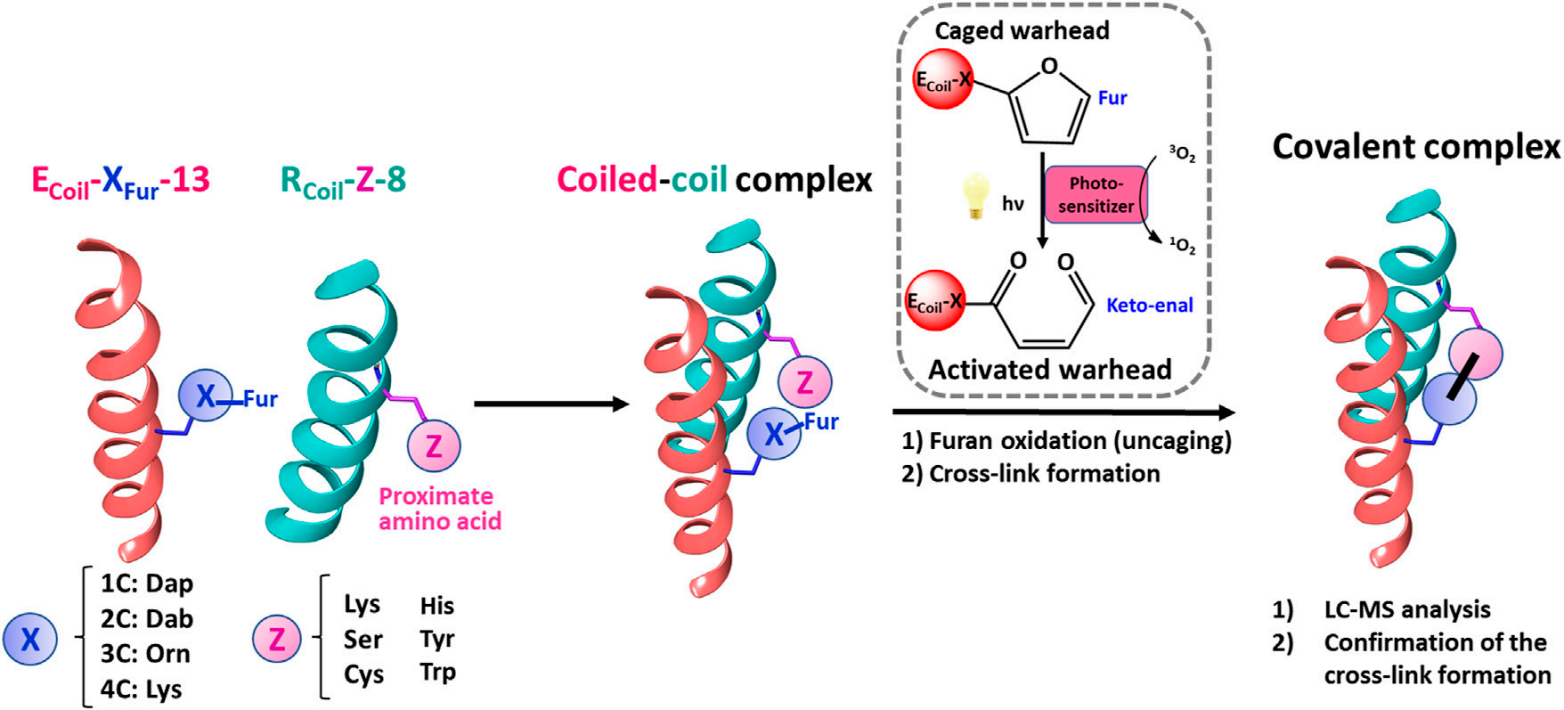
Schematic representation of the strategy followed to explore furan reactivity in an E3/R3 coiled-coil peptide dimer. To scan the furan warhead reactivity towards additional cross-link partners, E_Coil_-X_Fur_-13 and R_Coil_-Z-8 were chemically synthesized. Dap, Dab, Orn, and Lys were introduced at position 13 on the E_Coil_ as handles to introduce the Fur moiety. These four amino acids featuring varying lengths of methylene spacers were used to study the optimal distance between the furan moiety and the peptide backbone to increase the efficiency of cross-linking between the coiled-coil motifs. Ser, Cys, His, Tyr, and Trp, besides Lys, were chosen as potential new partners for the furan warhead and were placed at position 8 on the R_Coil_. All possible E/R coiled-coil partners were pre-incubated to allow coiled-coil complex formation, and then PSs (RB or Rhd B) were added and irradiated with light to afford covalently connected coiled-coil motifs upon furan activation.

To further evaluate the influence of chain length and positioning of the furan moiety on the cross-link reaction, Lys (4C chain), Dab (2,4-diaminobutyric acid, 3C chain), and Dap (2,4-diaminopropionic acid, 1C chain) were introduced at position 13 on the E_Coil_ as handles to couple the Fur moiety (Figure 7 and ESI 3.4-3.6). These three amino acids together with Orn, featuring varying lengths of methylene spacers between the terminal amino group of the side chain and the *α*-carbon atom, were used to study the optimal distance between the furan moiety and the peptide backbone. All HPLC and MS analyses of reaction mixtures between R_Coil_-Lys-8 and E_Coil_-X_Fur_-8 (with X = Lys, Orn, Dab, or Dap) have been included in Supplementary Materials (see ESI, section 5.3–5.6). In all cases, high cross-link levels were achieved although the ratio between the expected covalent product (4-ketoamide) and the −18 Da form (aldimine) differs, the last one becoming the main product when using E_Coil_-Dap_Fur_-13 (see ESI 5.6, Supplementary Figures S23–S24). It is important to mention that high levels of doubly oxidized covalent product (+32 Da) were observed when using E_Coil_-Lys_Fur_-13 and E_Coil_-Dab_Fur_-13 (see ESI 5.3 and 5.5, Supplementary Figures S18, S22); however, the oxidation level of the cross-linked product can be tuned by the type of PS and by the PS properties (i.e., dose and time dependency of ^1^O_2_ production). The secondary structure of the R_Coil_ and E_Coil_ peptides can be monitored experimentally by circular dichroism (CD). The CD spectra of the R_Coil_-Lys-8, E_Coil-_Dap_Fur_-13, and E_Coil-_Lys_Fur_-13, as well as 1:1 mixture of E and R coil were recorded (see ESI 2.10, 5.7 and 5.8, Supplementary Figures S25, S26). E_Coil-_Dap_Fur_-13 and E_Coil-_Lys_Fur_-13 with the shortest and longest furan-side chain length were chosen for CD analysis. The CD spectra are reported as the mean residue molar ellipticity ([θ]) and demonstrate that R_Coil_-Lys-8 forms a coiled-coil domain with both E_Coils_ with the characteristic minima at 208 and 222 nm as previously reported for the heterodimeric E3/K3 coiled-coil model system (Litowski and Hodges, 2002). The negative molar ellipticity ([θ]) at 222 nm is directly proportional to the amount of helical structure and the (θ)_222_/(θ)_208_ ratio is typically ˃1.0 for E3/K3 coiled-coil helical heterodimers and 0.66–0.72 for the single *α*-helices E3 and K3 coils as previously reported by Litowski and Hodges (2002) (see Supplementary Table S1 in ESI 2.10). The 1:1 mixture of both E_Coils_ with R_Coil_-Lys-8 had the largest (θ) at 222 nm (see ESI 5.7-5.8, tables in Supplementary Figures S25, S26), indicating that the heterodimeric coiled-coils have the maximum helical structure. Note that transformation of the K_Coil_ to an R_Coil_ as well as the introduction of the furan-side chain at position 13 (residue on position e, which is not important for the hydrophobic core) on the E_Coil_ do not interfere in coiled-coil formation. Furan cross-linking is described for the first time to constrain coiled-coil helical dimers and demonstrates that the E3/R3 coiled-coil system is an ideal model to explore furan reactivity.

### Scanning of Furan Warhead Towards Novel Cross-Link Partners in a Coiled-Coil Peptide Dimer

To evaluate whether other amino acids can cross-link with the furan warhead, three nucleophilic amino acids, cysteine (Cys), histidine (His), and serine (Ser), and two amino acids that can potentially engage in electrophilic aromatic substitution, tyrosine (Tyr) and tryptophan (Trp), were selected and incorporated at position 8 on the R_Coil_ (Figure 7). After chemical synthesis (see ESI 3.7-3.11), the secondary structure of the peptides was evaluated by CD spectroscopy for all R_Coils_ and E_Coil-_Lys_Fur_-13, as well as a mixture containing both (see ESI 6). All the R_Coils_ formed heterodimeric coiled-coils with E_Coil-_Lys_Fur_-13 with the characteristic minima at 208 and 222 nm and the largest [θ] at 222 nm (see ESI 6, Supplementary Figures S27–S31). Note that the introduction of different amino acids at position 8 (residue on position g) on the R_Coil_ does not alter the coiled-coil nature. Next, the efficiency of the E_Coil_-Orn_Fur_-13 to cross-link to the new R_Coils_ was examined using the two PS-irradiation protocols as described above, and the reaction mixtures after light irradiation were investigated by HPLC analysis (see ESI 7). Rhd B and RB-irradiation protocols show evidence of cross-linked product for Cys and Tyr-R_Coil_ to E_Coil_-Orn_Fur_-13 (see ESI 7, Supplementary Figures S33, S35, S38, S40). In addition, R_Coil_-Trp-8 seems to cross-link to E_Coil_-Orn_Fur_-13 using Rhd B albeit with a lower cross-link yield (see ESI 7.1.5, Supplementary Figure S36). Note that the GPR54/KP-10 homology model allowed visualization of GPR54-C62 and C277 in close proximity to KP-10-W3 residue (Figure 2A; Supplementary Figure S2 in ESI 1). In addition, several GPR54-Tyr residues, as well as several Actin-Tyr residues were observed in sufficient proximity of the KP-10-W3 (see Figure 2A; Supplementary Figure S2 in ESI 1) or T*β*4-E24 (Figure 2B), respectively. All these residues were identified as potential furan-cross-link partners when KP-10-W3 or T*β*4-E24 are replaced by Fua, and we indeed prove site-specific cross-linking of Cys and Tyr side chains towards the furan warhead after activation (see below).

**FIGURE 8:**
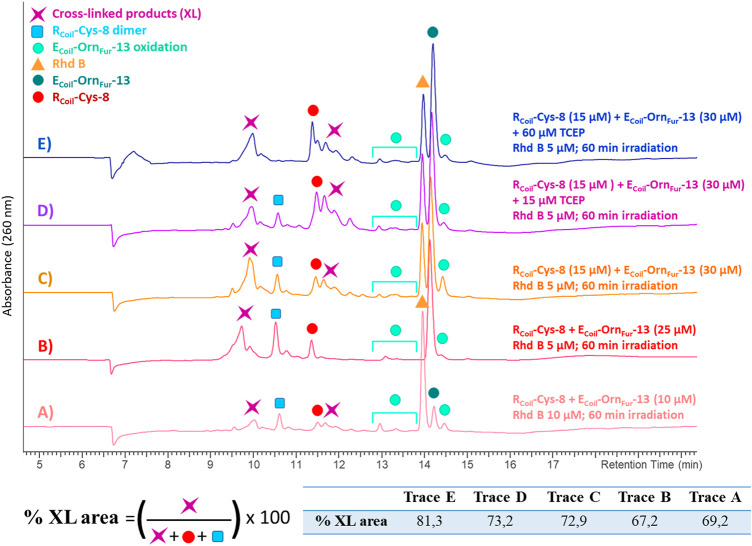
All the chromatograms show the cross-linking reaction product (purple star) between R_Coil_-Cys-8 (red dot) and E_Coil_-Orn_Fur_-13 (dark green dot) after light irradiation with Rhd B (orange triangle). The E_Coil_-Orn_Fur_-13 oxidation products (green light dots) are produced after activation of the furan moiety by singlet oxygen generation. **(A)** R_Coil_-Cys-8 (10 μM) and E_Coil_-Orn_Fur_-13 (10 μM), Rhd B 10 μM and 60 min of light irradiation. **(B)** R_Coil_-Cys-8 (25 μM) and E_Coil_-Orn_Fur_-13 (25 μM), Rhd B 5 μM and 60 min of light irradiation. **(C)** R_Coil_-Cys-8 (15 μM) and E_Coil_-Orn_Fur_-13 (30 μM), Rhd B 5 μM and 60 min of light irradiation. **(D)** R_Coil_-Cys-8 (15 μM) + 15 μM TCEP and E_Coil_-Orn_Fur_-13 (30 μM), Rhd B 5 μM and 60 min of light irradiation. **(E)** R_Coil_-Cys-8 (15 μM) + 60 μM TCEP and E_Coil_-Orn_Fur_-13 (30 μM), Rhd B 5 μM and 60 min of light irradiation. The cross-link yield was quantified as the percentage of the cross-linked product area and the values are indicated in the table.

Cys was the only nucleophilic amino acid, besides Lys, to cross-link with the Fur moiety. To further increase the level of cross-link efficiency between R_Coil_-Cys-8 and E_Coil_-Orn_Fur_-13 (ensuring furan-activation) while minimizing oxidation of the cross-linked products, Rhd B was chosen as a PS in view of the lower oxidation levels of R_Coil_-Cys-8 when ^1^O_2_ is produced in the absence of the Fur-E_Coil_ compared to RB (see ESI 7, Supplementary Figures S33, S38). The concentration of both coils was increased from 10 to 25 μM (Figures 8A,B, respectively) to study the dose dependency of the coiled-coil cross-link yield. A higher UV absorbance was observed in the chromatograms, related to the increase in E3/R3 coil concentration, but there were no differences in terms of cross-link yields (69.2% and 67.2%, respectively, see table in Figure 8; Supplementary Table S4 in ESI 8.2). This confirms the almost complete association of monomers to the coiled-coil dimer at 10–25 μM as previously described for R_Coil_-Lys-8 and E_Coil_-Orn_Fur_-13.

We note that the R_Coil_-Cys-8 cross-links by itself to give a disulfide bond dimer when singlet oxygen is produced (see ESI 8.3, Supplementary Figures S43, S44). Disulfides formed from oxidation of two Cys residues by singlet oxygen production *via* the thiyl radical intermediates and subsequent dimerization of these species has been previously reported (Schöneich, 2016; Trujillo et al., 2016). Moreover, the disulfides can further react with singlet oxygen to form zwitterionic peroxides, thiosulfinates, and thiosulfonates (Clennan et al., 1997; Clennan, 2001). We envisaged that by changing the E_Coil_:R_Coil_ ratio from 1:1 to 2:1 (Figures 8B,C, respectively), R_Coil_–R_Coil_ interactions would become less important, as well as the level of Cys-mediated dimerization, and the level of E3/R3 coiled-coil cross-linking would improve (Figure 8C). However, the excess of E_Coil_-Orn_Fur_-13 was not enough to completely prevent R_Coil_-Cys-8 dimerization. R_Coil_-Cys-8 was pre-treated with tris(2-carboxyethyl)phosphine (TCEP) in a 1:1 ratio for 10 min before adding E_Coil_-Orn_Fur_-13 and starting the Rhd B-light irradiation protocol (Figure 8D). TCEP is known as a reducing agent and is used to break the disulfide bonds and keep cysteines in their reduced form. A total of 4 equivalents (equiv.) of TCEP were needed to stop R_Coil_-Cys-8 dimerization and to obtain a high level of coiled-coil cross-linked product (81.3%, Figure 8E, Supplementary Table S4 in ESI 8.2, and ESI 8.5). The optimized protocol (RCoil: 15 μM + 60 μM TCEP, ECoil: 30 μM, Rhd B: 5 μM, and 60 min of light irradiation) was used to cross-link R_Coil_-Cys-8 with all E_Coil-_X_Fur-_13 and the reaction mixtures were investigated by HPLC and MS analysis (all chromatograms and spectra can be found in ESI 8.4-8.7). As previously reported for Lys, high cross-link levels were also achieved with R_Coil_-Cys-8 with all Fur-E_Coil_ (E_Coil-_X_Fur-_13, see ESI 3.1, Supplementary Table S2). The MS analyses (see ESI 8.4-8.7, Supplementary Figures S45, S47, S49, S51) show peaks at different retention times with the same cross-linked product mass. The mass of the cross-linked product corresponds to the Michael-type addition of the cysteine thiol group of R_Coil_-Cys-8 to the *α*,*β*-unsaturated double bond of the activated furan moiety (keto-enal-E_Coil_) after generation of singlet oxygen. The addition of the thiol may occur at the *α-* or *β*-position relative to the ketone group of the activated furan, and possibly all cross-linked products are mixtures of R and S enantiomers (see ESI 8.1, Supplementary Figure S42). That explains why various cross-linked product peaks are observed in the HPLC chromatograms. In addition to the Michael-type addition cross-linked product, the dehydrated and the oxidized form of the cross-linked product (–18 Da and +16 Da, respectively) can be observed in small quantities, as well as R_Coil_-Cys-8 dimer.

**FIGURE 9:**
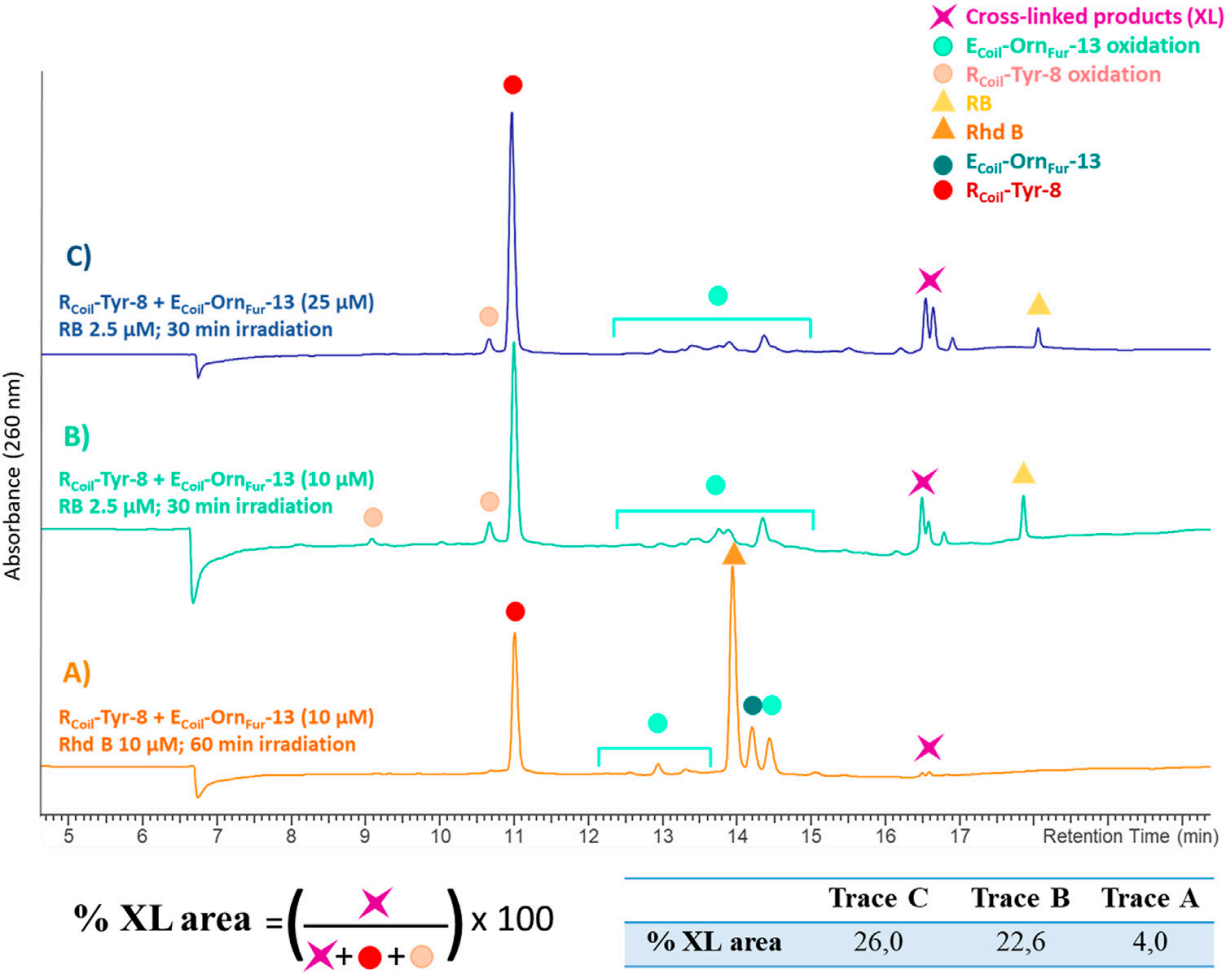
All chromatograms show the cross-link reaction product (purple star) between R_Coil_-Tyr-8 (red dot) and E_Coil_-Orn_Fur_-13 (dark green dot) after light irradiation with Rhd B (orange triangle) and RB (yellow triangle). The E_Coil_-Orn_Fur_-13 oxidation products (green light dots) are produced after activation of the furan moiety by singlet oxygen generation. **(A)** R_Coil_-Tyr-8 (10 μM) and E_Coil_-Orn_Fur_-13 (10 μM), Rhd B 10 μM and 60 min of light irradiation. **(B)** R_Coil_-Tyr-8 (10 μM) and E_Coil_-Orn_Fur_-13 (10 μM), RB 2.5 μM and 30 min of light irradiation. **(C)** R_Coil_-Tyr-8 (25 μM) and E_Coil_-Orn_Fur_-13 (25 μM), RB 2.5 μM and 30 min of light irradiation. The cross-link yield was quantified as the percentage of the cross-linked product area and the values are indicated in the table.

In addition, the activated Fur moiety cross-links with amino acids that can engage in electrophilic aromatic substitution: Tyr and Trp; however, the cross-linking efficiency for Trp (only observed when using Rhd B) is lower as compared to Tyr (cross-linked product is observed for both PS; however, cross-link yield is higher when using RB, see Figures 9A,B and ESI 7.1.4, Supplementary Figure S35 in comparison with 40 in 7.2.4). The level of cross-linking between R_Coil_-Tyr-8 and E_Coil_-Orn_Fur_-13 was slightly increased by increasing the concentration of both coils up to 25 μM using RB (from 22.6% to 26.0%, see Figures 9B,C and ESI 9.2). No differences were observed in terms of cross-link efficiency upon increasing the E_Coil_-to-R_Coil_ ratio from 1:1 to 2:1 and increasing the ^1^O_2_ production time (data not shown). Note that the RB-irradiation protocol produces oxidation-induced damage to Tyr, but cross-link yields are higher compared to Rhd B (See Figures 9A,B). E_Coil_-Dab_Fur_-13 (2 methylene spacers) and E_Coil-_Dap_Fur_-13 (1 methylene spacer) with a shorter side chain did not cross-link or cross-linked in much lower yields to R_Coil_-Tyr-8, respectively (see ESI 9.5-9.6, Supplementary Figures S58, S59). The E_Coil_-Dap_Fur_-13 mass analysis reveals a mini-peak with a high molecular mass that could correspond to a cross-linked product; however, more detailed characterization was not possible (see ESI 9.6, Supplementary Figure S60). E_Coil_-Lys_Fur_-13 cross-links to R_Coil_-Tyr-8 with similar efficiency to the E_Coil_-Orn_Fur_-13 (see ESI 9.3-9.4, Supplementary Figure S54 in comparison with Supplementary Figure S56). We propose that R_Coil_-Tyr-8 cross-links to E_Coil_-Lys_Fur_-13 through an electrophilic aromatic substitution, after which aromatic rearrangements and loss of water occur to give the final product (see ESI 9.1, Supplementary Figure S53 for details of the proposed structure). The calculated mass of this structure correlates with the found mass for the cross-linked product (see ESI 9.3, Supplementary Figure S55); however, when shortening the connecting chain to the furan (cfr. cross-link experiments of R_Coil_-Tyr-8 with E_Coil_-Orn_Fur_-13), the corresponding adduct could not be elucidated in detail. These results indicate that the longest chains (E_Coil_-Lys_Fur_-13 and E_Coil_-Orn_Fur_-13) with more flexibility can accommodate the cross-linked product with the most thermodynamically stable structure.

The R_Coil_-Trp-8 cross-links to E_Coil_-Orn_Fur_-13 with low cross-link yield. The best cross-link efficiency was achieved using an E_Coil_:R_Coil_ ratio of 1:1 with Rhd B at 10 μM and light irradiation for 120 min (see ESI 10.2, Supplementary Figures S62, S63). No cross-link product was observed for the other E_Coil_-X_Fur_-13 coil peptides (see ESI 10.1 and 10.3).

## Conclusion

In previous work, the furan-cross-link technology was applied to KP-10-GPR54 and T*β*4-Actin complex systems, and peptide–protein and protein–protein cross-link products were observed. The formed cross-link complex was shown to result from the reactivity of the furan warhead (incorporated in a peptide or protein) towards a Lys (in close proximity) in the target protein. However, the KP-10-GPR54 homology model as well as the crystal structure of Actin-T*β*4 (4PL7) (Figure 2) indicate that besides Lys, other side-chain amino acids can be located in close proximity to the activated furan moiety. The E3/R3 coiled-coil model system used here, now enabled firm identification of Cys and Tyr as new nucleophilic partners, able to react and form a covalent bond. At the same time, the system allowed to study the optimal distance between the furan moiety and the peptide backbone.

In conclusion, we have reported that the replacement of weak interhelical ionic contacts with suitable precursors for furan-oxidation-based cross-linking affords stable dimeric coiled-coil structures that can be covalently cross-linked upon visible light irradiation. We describe for the first time reaction of the oxidized furan moiety with cysteine and tyrosine residues in the target strand, in addition to the earlier reported lysine-oriented furan warhead (Miret-Casals et al., 2021a). It is important to note that unnatural furan-containing amino acids can be incorporated in peptides and proteins by either flexible *in vitro* translation (Decoene et al., 2018) or genetic encoding of noncanonical amino acids, in *Escherichia coli* (Schmidt and Summerer, 2013) and in human cells (Schmidt et al., 2014), respectively. This study suggests that furan warheads, when incorporated into peptides or proteins, can be used for oxidation-induced cross-linking to a variety of amino acid side chains in the target protein when spatial proximity is ensured to discover weak and/or transient protein–ligand and protein–protein interactions.

## Materials and Methods

### Synthesis, Purification, and Characterization of Coil Peptides

Chemicals, general procedures, solid phase peptide synthesis, purification, and characterization of the peptides are described in the Supplementary Materials section of this paper (see ESI 2 and 3).

### Circular Dichroism Spectroscopy

CD spectra of the E/R_Coils_ analogues, as well as a mixture containing both, were obtained using a JASCO J7100 instrument (Tokyo, Japan), equipped with a HAAKE cryostat temperature-controlled cell holder at 25°C. CD spectra are reported as the mean residue molar ellipticity ([θ]) with units of degrees square centimeter per decimole (deg x cm^2^/dmol), calculated by the equation:

$$\left[\theta \right]=\left(\theta_{\text{obs}}\times \text{MRW}\right)/\left(10lc\right)$$

where θ_obs_ is the ellipticity in millidegrees, MRW is the mean residue molecular weight (molecular weight of the peptide divided by the number of amino acid residues), l is the path length of the cuvette in centimeters, and c is the peptide concentration in milligrams per milliliter. The CD spectra were recorded at 50 nm/min scan rate, a bandwidth of 1 nm, a data pitch of 0.1 nm, a response of 0.5 s, a wavelength range of 200–260 nm, and a 1-cm path length cell. Each spectrum was an average of nine scans. Baselines were corrected by subtracting the solvent contribution [phosphate-buffered saline (PBS) 1 × buffer]. The CD spectra for all R_Coil_-Z-8 and E_Coil_-Lys_Fur_-13 were measured at 5 μM in PBS (pH 7.4) for each coil peptide, as well as a 1-to-1 mixture containing both; see Supplementary Materials for further details (see ESI 2.10).

### Cross-Linking Experiments and Singlet Oxygen Production

The concentrated R_Coil_-Z-8 and E_Coil_-X_Fur_-13 peptides (100 μM) were diluted in air-saturated PBS (pH 7.4). The used concentration of coil peptides is indicated in figures or figure legends. Cross-linking experiments took place in 2-ml Eppendorf vials in a total volume of 300 μl placed. R_Coil_-Z-8 and E_Coil_-X_Fur_-13 peptides were incubated for 5 min at room temperature (binding step), and then the photosensitizers, Rhodamine B or Rose Bengal, were added to the mixture to a final concentration of 10 or 2.5 μM, respectively (or as indicated in figures). The lamp was then placed on top of the Eppendorf and the samples were irradiated with a Euromex Illuminator Eκ-1 lamp (110 W, 12 V, halogen lamp LE.5210) coupled with an optical fiber arm (Euromex LE.5214 dual-arm light conductor) at room temperature for 60 and 30 min, respectively (or as indicated in figures). The light intensity of the lamp was kept in between 6.8 and 7 Klux. After irradiation, the reaction mixture was left to react for 1 h at 25°C, and the samples were submitted to HPLC-UV and HPLC-MS analysis; see Supplementary Materials for further details (see ESI 2.9).

## Data Availability

The original contributions presented in the study are included in the article/Supplementary Material. Further inquiries can be directed to the corresponding authors.
