# Supplementary material for: Equipping Coiled-Coil Peptide Dimers With Furan Warheads Reveals Novel Cross-Link Partners
Source: Front Chem. 2022 Feb 16;9:799706. doi: 10.3389/fchem.2021.799706 (PMC8888431; doi:10.3389/fchem.2021.799706)
Supplement: Supplementary file 1 [file DataSheet1.PDF]

Supplementary Material

Table of Contents

1 Homology modeling KISS1R (GPR54)....4

2 Materials and methods....6

2.1 Materials....6

2.2 Peptide synthesis....6

2.3 Semi-Preparative RP-HPLC purification....7

2.4 Reversed Phase High-Performance Liquid Chromatography....7

2.5 Freeze drying....7

2.6 MALDI-TOF Mass Spectrometry....7

2.7 Liquid Chromatography-Electrospray ionization-Mass Spectrometry (LC-ESI-MS)....8

2.8 Peptide concentration determination and storage....8

2.9 Cross-linking experiments....8

2.10 Circular dichroism (CD) spectroscopy....9

3 R_{Coil} and E_{Coil} peptides characterization....11

3.1 Table of the R_{Coil} and E_{Coil} synthesized peptides....11

3.2 R_{Coil}-Lys-8 peptide....12

3.3 E_{Coil}-Orn_{Fur}-13 peptide....13

3.4 E_{Coil}-Lys_{Fur}-13 peptide....14

3.5 E_{Coil}-Dab_{Fur}-13 peptide....15

3.6 E_{Coil}-Dap_{Fur}-13 peptide....16

3.7 R_{Coil}-Ser-8 peptide....17

3.8 R_{Coil}-Cys-8 peptide....18

3.9 R_{Coil}-His-8 peptide....19

3.10 R_{Coil}-Tyr-8 peptide....20

3.11 R_{Coil}-Trp-8 peptide....21

4 R_{Coil}-Lys-8 cross-links with E_{Coil}-Orn_{Fur}-13....22

4.1 RB as a photosensitizer....22

4.2 Rhd B as a photosensitizer....24

5 Optimization of cross-linking conditions for Lys....25

5.1 How to calculate the percentage of cross-linked product area....25

5.2 Quantitative analysis of the cross-link yield for Figure 5 in the manuscript 25
5.3 Cross-linked product of R_{Coil}-Lys-8 with E_{Coil}-Lys_{Fur}-13..26
5.4 Cross-linked product of R_{Coil}-Lys-8 with E_{Coil}-Orn_{Fur}-13 28
5.5 Cross-linked product of R_{Coil}-Lys-8 with E_{Coil}-Dab_{Fur}-13 30
5.6 Cross-linked product of R_{Coil}-Lys-8 with E_{Coil}-Dap_{Fur}-13 32
5.7 CD spectroscopy of R_{Coil}-Lys-8 and E_{Coil}-Dap_{Fur}-13...34
5.8 CD spectroscopy of R_{Coil}-Lys-8 and E_{Coil}-Lys_{Fur}-13 35

6 CD spectroscopy of R_{Coils} with E_{Coil}-Lys_{Fur}-13 peptide 36

6.1 CD spectroscopy of R_{Coil}-Ser-8 and E_{Coil}-Lys_{Fur}-13 36
6.2 CD spectroscopy of R_{Coil}-Cys-8 and E_{Coil}-Lys_{Fur}-13...37
6.3 CD spectroscopy of R_{Coil}-His-8 and E_{Coil}-Lys_{Fur}-13 38
6.4 CD spectroscopy of R_{Coil}-Tyr-8 and E_{Coil}-Lys_{Fur}-13 39
6.5 CD spectroscopy of R_{Coil}-Trp-8 and E_{Coil}-Lys_{Fur}-13...40

7 R_{Coil} scans with E_{Coil}-Orn_{Fur}-13 peptide... 41

7.1 Rhodamine B as a photosensitizer...41
7.1.1 R_{Coil}-Ser-8 scan with E_{Coil}-Orn_{Fur}-13...41
7.1.2 R_{Coil}-Cys-8 scan with E_{Coil}-Orn_{Fur}-13 42
7.1.3 R_{Coil}-His-8 scan with E_{Coil}-Orn_{Fur}-13...43
7.1.4 R_{Coil}-Tyr-8 scan with E_{Coil}-Orn_{Fur}-13...44
7.1.5 R_{Coil}-Trp-8 scan with E_{Coil}-Orn_{Fur}-13...45
7.2 Rose Bengal as a photosensitizer...46
7.2.1 R_{Coil}-Ser-8 scan with E_{Coil}-Orn_{Fur}-13...46
7.2.2 R_{Coil}-Cys-8 scan with E_{Coil}-Orn_{Fur}-13 47
7.2.3 R_{Coil}-His-8 scan with E_{Coil}-Orn_{Fur}-13...48
7.2.4 R_{Coil}-Tyr-8 scan with E_{Coil}-Orn_{Fur}-13...49
7.2.5 R_{Coil}-Trp-8 scan with E_{Coil}-Orn_{Fur}-13...50

8 Optimization of cross-linking conditions for Cys 51

8.1 Cross-link between R_{Coil}-Cys-8 and E_{Coil}-X_{Fur}-136...51
8.2 Quantitative analysis of the cross-link yield for Figure 7 in the manuscript 51
8.3 R_{Coil}-Cys-8 dimerization by irradiation with Rhodamine B...52
8.4 Cross-linked product of R_{Coil}-Cys-8 with E_{Coil}-Lys_{Fur}-13 54
8.5 Cross-linked product of R_{Coil}-Cys-8 with E_{Coil}-Orn_{Fur}-13 56
8.6 Cross-linked product of R_{Coil}-Cys-8 with E_{Coil}-Dab_{Fur}-13..58

8.7 Cross-linked product of R_{Coil}-Cys-8 with E_{Coil}-Dap_{Fur}-13 60

9 Optimization of cross-linking conditions for Tyr 62

9.1 Cross-link between R_{Coil}-Tyr-8 and E_{Coil}-Lys_{Fur}-13 62

9.2 Quantitative analysis of the cross-link yield for Figure 8 in the manuscript 63

9.3 Cross-linked product of R_{Coil}-Tyr-8 with E_{Coil}-Lys_{Fur}-13 64

9.4 Cross-linked product of R_{Coil}- Tyr-8 with E_{Coil}-Orn_{Fur}-13 66

9.5 Cross-linked product of R_{Coil}- Tyr-8 with E_{Coil}-Dab_{Fur}-13 68

9.6 Cross-linked product of R_{Coil}- Tyr-8 with E_{Coil}-Dap_{Fur}-13 69

10 Optimization of cross-linking conditions for Trp 71

10.1 Cross-linked product of R_{Coil}-Trp-8 with E_{Coil}-Lys_{Fur}-13 71

10.2 Cross-linked product of R_{Coil}- Trp-8 with E_{Coil}-Orn_{Fur}-13 72

10.3 Cross-linked product of R_{Coil}- Trp-8 with E_{Coil}-Dab_{Fur}-13 74

11 REFERENCES 75

Supplementary Material

1 Homology modeling KISS1R (GPR54)

KISS1R belongs to the A class of the GPCR family (transmembrane protein, Rhodopsin-like), but no structural data about this receptor is currently available. In order to create an homology model of KISS1R, we used the BLAST web server (v 2.3.0) to search about potential templates, and three GPCRs were identified: Nociceptin receptor (% identity: 32; % similarity: 52, PDB ID: 4EA3), delta-opioid receptor (% identity: 32; % similarity: 51, PDB ID: 4DJH), and kappa-opioid receptor (% identity: 31; % similarity: 50, PDB ID: 4N6H). All those structures are in the inactive state of KISS1R.

Sequence alignment was applied using the web server MAFFT version 7 [1] and visualized with Jalview version 2.10 [2]. The alignment was manually checked to verify the conserved structural motifs of class A GPCR: (GN motif on TM1, LXXXD motif on TM2, DRX motif, and cysteine involved in conserved disulphide bridge on TM3).

The alignment validates all the structural features of class A GPCR. The homology model of KISS1R was built using Modeller software version 9.13 [3]. Particular attention was devoted to the extracellular loop 1, associated to a potential gate of the receptor cavity, and to the intracellular loop 3, associated to the ionic lock driving the active and inactive states. A loop refinement was carried out using default parameters. 10 models were generated and model 7 presents the best Ramachandran plot. Procheck analysis was applied to validate this model [4]. The ionic lock, allowing the stability of the inactive state is defined by D139 and R258. Finally, W276 is the toggle switch residue, which provokes the activation of the receptor by its interaction with an agonist. Molecular dynamics (MD) simulation of 1 µs was applied with Amber16 [5] to relax the generated structure and one representative structure was extracted to finally propose two starting points for the prediction of the interactions between KP10 and GPR54.

<u>Prediction of KP10-GPR54 complex</u>

In this study, we used the Iterative Residue Docking and Linking method (IRDL), which has been developed for the aim of peptide-protein interaction prediction [6]. To summarize, the IRDL method aims at solving the docking problem of peptides by splitting them into fragments of 2 or 3 amino acids and linking them by creating a covalent peptide bond between two docked fragments. A classical docking method is applied to the first fragment in the binding cavity. Next, the docking of the second fragment is realized. Second, the ligation between the two fragments is performed to create a peptide bond. When the covalent bond is created, an energy minimization is realised on the atoms involved in the peptide bond. These steps are repeated until the full peptide is built in the binding cavity. Finally, a rescoring step is carried out in order to rank all the docking poses. In this study, the KP10 peptide was split in four fragments, as described in Sup. Fig. 1.

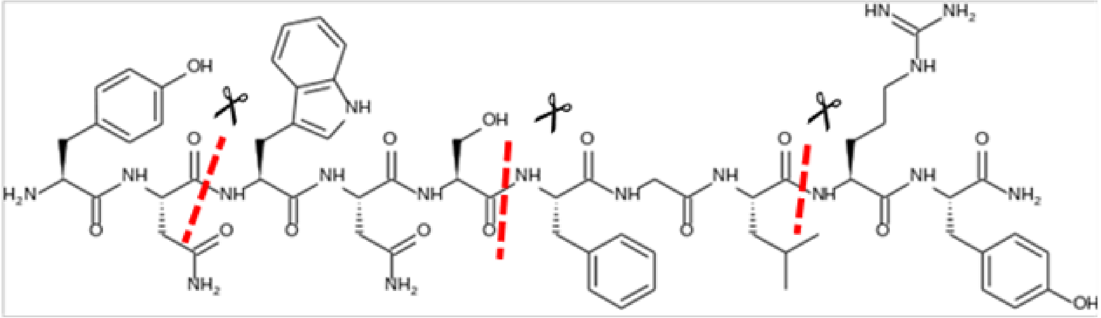

Supplementary Figure 1. Structure of the kisspeptin-10 (KP10) peptide. The red lines represent the cutting bonds used by IRDL.

Two conformations of the receptor were considered for the prediction: the original homology model and the relaxed structure extracted from molecular dynamics simulations. From those two structures, three steps of IRDL methodology were realised to propose a model of the KP10-GPR54 complex. The best model using the two conformations of the receptor (rigid and relaxed) was retained for analysis. The best predicted docking poses of the native kisspeptin-10 ligand into the GPR54 model receptor structure using the rigid (see Sup. Fig. 1A) and the relaxed (see Sup. Fig. 1B) conformation of the receptor show that several GPR54-tyrosine (Y) residues, as well as GPR54-tryptophan (W), -cysteine (C), and -lysine (K) are located in sufficient proximity of the KP-10-W3 residue (see Sup. Fig. 1) and could cross-link when W3 is replaced by Fua.

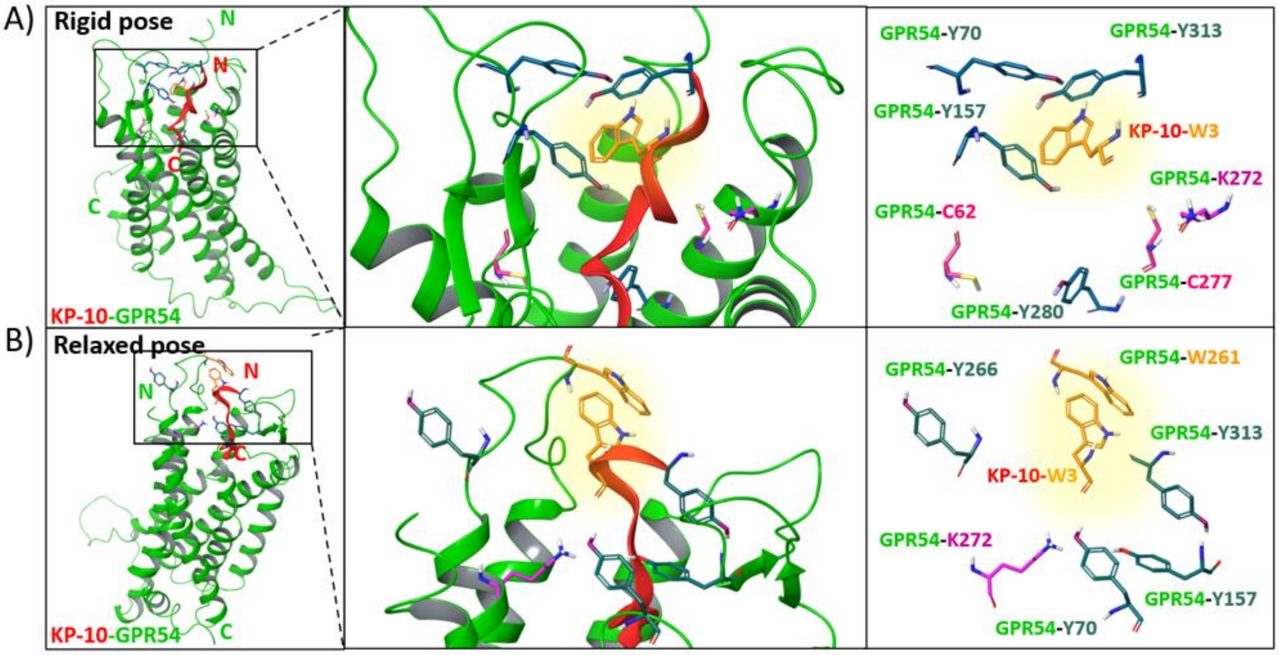

Supplementary Figure 2. A-B) The best predicted docking poses of the native kisspeptin-10 ligand into the GPR54 model receptor structure using two conformations of the receptor: the rigid pose and the relaxed pose state. Left panels: overview of the complex system; middle panels: zoom in on GPR54/ACTIN amino acids side chains (Lys, Cys, Tyr, and Trp) proximate to KP-10-W3; right panels: GPR54/ACTIN amino acids side chains close to W3 in KP-10 without the secondary structure. The

amino acid that is replaced by furylalanine (W3 in kisspeptin) is highlighted with a yellow background. *N, C = N-, C-termini.* Images prepared using MAESTRO.

2 Materials and methods

2.1 Materials

All-natural amino acids, coupling reagent 2-(1H-benzotriazol-1-yl)-1,1,3,3-tetramethyluronium hexafluorophosphate (HBTU), trifluoroacetic acid (TFA), and hexafluoroisopropanol (HFIP) were purchased from Iris Biotech GmbH. All peptides were synthesized on the ChemMatrix Rink Amide resin with a loading of 0.5 mmol/g (0.4-0.6). The resin was acquired from Thermo Scientific. Peptide synthesis grade dimethylformamide (DMF) was purchased from Biosolve. Dichloromethane (DCM), *N*,*N*-Diisopropylethylamine (DIPEA), *N*-methylmorpholine (NMM), triisopropylsilane (TIS), and 3-(2-Furyl)propionic acid were obtained from Sigma Aldrich.

2.2 Peptide synthesis

All peptides were synthesized on a SYRO Multiple Peptide Synthesizer (Multisyntech) or on a MultiPep RSi (Intavis) automated peptide synthesiser using the tBu/Fmoc strategy. The synthesis of the R_{Coil} and E_{Coil} peptides was performed using 100 mg of the Chemmatrix rink amide resin with a loading of 0.5 mmol/g (50 mmol scale). Synthesis with double coupling steps was performed as following: the resin was swollen in DMF for 20 minutes. A mixture of 5 equiv. amino acid in DMF (0.5 M), 5 equiv. HBTU in DMF (0.5 M) and 10 equiv. DIPEA in NMP (2 M) were added to the resin, with subsequent reaction for 40 minutes at room temperature. The reaction mixture was removed, and the resin was washed with DMF (4 x 30 sec.). Every coupling was repeated a second time. Fmoc group was removed using 40% piperidine in DMF. The 4-aminobenzoic acid (Aba) was coupled at the N-terminus after Fmoc-deprotection as described previously for standard amino acid coupling. To introduce the 3-(2-Furyl)propioinic acid (Fur) in the E_{Coil} peptides, Lys(Mtt), Orn(Mtt), Dab(Mtt), or Dap(Mtt) were incorporated at position 13 in the automated peptide synthesis. Then, the Mtt (4-methyltrityl) protecting group was removed manually by washing the resin with a solution of HFIP (Hexafluoro-2-propanol)/DCM 1:1 with 0.5% HOBt used as a scavenger for the Mtt cations. The resin was washed six times for three minutes with the deprotection cocktail. After each washing, the deprotection cocktail was filtered off and a few drops of TFA were added. Once the deprotection cocktail remained colourless after adding TFA, the Mtt deprotection was completed. Then, Fur was manually coupled at the free amine of the side chain of Lys/Orn/Dab and Dap using 4 equiv. Fur and HBTU, 8 equiv. of DIPEA, and the resin was shaken for two hours. The completion of the reaction was checked by the TNBS test. A few beads were added to a small test tube, followed by treatment with 10 µL 10% DIPEA/DMF and 10 µL TNBS at room temperature. Free amines were detected by a red colour change on the beads. Cleavage of the R_{Coil} peptides was performed during 1.5 h at room temperature using the following cleavage cocktail: 95% TFA, 2.5% TIS, and 2.5% H_2O. After reaction, the resin was removed by filtration and the majority of the cleavage cocktail was removed by nitrogen evaporation. Cold MTBE or Et_2O was added in excess to further precipitate the peptide, followed by sonication and centrifugation (5’, 7500 rpm at 4°C). The supernatant was discarded and a fresh volume of MTBE or Et_2O was added to repeat sonication and centrifugation. This process was repeated three

times. The residual R_{Coil} peptides were dried with a gentle stream of nitrogen and then dissolved in H_2O/ACN to be analyzed by reversed phase high-performance liquid chromatography (RP-HPLC). The furan moiety of the E_{Coil} peptides is more sensitive to degradation during the cleavage step. For this reason, an optimized cleavage cocktail was employed to cleave these peptides: 90% TFA with 10% m-cresol. 10 µM of resin (20 mg) was treated for 30 minutes with 500 µL cleavage cocktail, after which it was filtered off. This was done twice. The resulting 1 mL of cleavage cocktail containing the desired peptide was diluted with petroleum ether (9 mL) and placed in an ice bath. The petroleum ether was then evaporated using a N_2 stream. Two more times petroleum ether was added and evaporated to eliminate any traces of TFA.The R_{Coil} and E_{Coil} peptides were subsequently purified using semi-preparative-HPLC, see §1.3.

2.3 Semi-Preparative RP-HPLC purification

The purification of the R_{Coil} peptides was performed on an Agilent 218 SEMI-PREP system with a UV-VIS dual wavelength detector using a Prepak cartridge (Delta-pak C18 100A) using a two solvent system A (H_2O + 0,1% TFA) and B (ACN + 0,1% TFA) with a flow rate of 65 mL/min. The column was eluted starting with a gradient from 100% A to 20% B in 4 minutes, then up to 30% B in 5 minutes, and a linear gradient up to 70% B for 40 minutes. The fractions containing the R_{Coil} peptides (47% B, 26 minutes) were collected. The E_{Coil} peptides were cleaved from the resin in a smaller scale, and the purification was done on a smaller scale as well. This was performed on a Agilent 218 solvent delivery system with a UV-VIS dual wavelength detector using a Phenomenex column (AXIA packed Luna C18(2), 250 x 21.2 mm, 5 µm particle size, 35°C) with a flow rate of 17.5 mL/min. The E_{Coil} peptides were eluted with a gradient using a two solvent system A (0.1% TFA in H_2O) and B (0.1% TFA in CH_3CN). The used method has a linear gradient from 0 to 100% B in 30 minutes.

2.4 Reversed Phase High-Performance Liquid Chromatography

RP-HPLC purity and cross-linking analyses were performed on an Agilent 1100 Series HPLC instrument equipped with a XTerra® Shield RP18, 125Å column (5µM 2,1 x 250mm) using a two solvent system A (0.1% TFA in H_2O) and B (0.1% TFA in CH_3CN). UV detection was done at 254 nm and the system was run at a flow rate of 350 µL/min at 40 °C. The gradient starts with 100% A and goes up to 30% B in 2.5 minutes. Then, a linear gradient starts from 30% B up to 60% B in 10 minutes. The gradient starts at 2.5 minutes and reaches 60% B at 12.5 minutes, with a loop delay of 5 minutes. Most shown HPLC-UV chromatograms are shown from 5 to 20 minutes to improve the visibility of the peaks.

2.5 Freeze drying

The R_{Coil} and E_{Coil} peptides were lyophilized in a Heto Drywinner freeze dryer in combination with a Thermoelectron corporation Savat SPD111V Speedvac concentrator or in a RVC 2-18 CDplus (Christ) in combination with an Alpha 2-4 LDplus (Christ) lyophilisator.

2.6 MALDI-TOF Mass Spectrometry

MALDI-TOF-MS data was acquired on a Sciex/Applied Biosystems 4800plus MaldiTOF/TOF analyser equipped with a Nd-YAG solid state laser (355 nm) and a pulse frequency of 200 Hz. For 1 mL matrix, 20 mg of α-cyano-4-hydroxycinnamic acid (α-CHCA) were dissolved in a solution

containing acetonitrile and 5% formic acid (7:3). Depending on the type of sample, different spotting methods were used. For simple peptide mass analysis, 0.5 µL of the α-CHCA matrix was spotted on the MALDI plate with 0.5 µL of the sample spotted on top of it. The cross-linking samples were spotted using a sandwich method, where 0.75 µL sample was spotted in between 0.5 µL of the α-CHCA matrix.

2.7 Liquid Chromatography-Electrospray ionization-Mass Spectrometry (LC-ESI-MS)

RP-HPLC-MS analyses were performed on an Agilent 1100 Series instrument with diode array detector, equipped with a Phenomenex Kinetex EVO/Phenomenex Kinetex C18 100 Å (150 x 4.6 mm, 5 µm, at 35 °C), hyphenated to an Agilent ESI-single quadrupole MS detector type VL. Mass detection operated in the positive mode. A two solvent system was used: 0.1% HCOOH in H_2O (A) and CH_3CN (B). Samples were eluted using a gradient from 0% to 100% B over 15 minutes at a flow rate of 1.5 mL/min at 35 °C.

2.8 Peptide concentration determination and storage

To prepare the 100 µM E3/K3 coil peptide stock solutions, the concentration was determined at the Trinean DropSense96 UV/VIS droplet reader at λ 280 nm.

2.9 Cross-linking experiments

The concentrated R_{Coil}-Z-8 and E_{Coil}-X_{Fur}-13peptides (100 µM) were 10-fold diluted (or as indicated in figures) an air saturated phosphate buffered saline (PBS, pH 7.4) containing 137 mM NaCl, 2.7 mM KCl, 10 mM Na_2HPO_4, and 1.8 mM KH_2PO_4. Cross-linking experiments took place in 2 mL Eppendorf vials in a total volume of 300 µL placed in a Thermomixer Comfort. R_{Coil}-Z-8 and E_{Coil}-X_{Fur}-13 peptides (10 µM, or as indicated in figures) were incubated for 5 minutes at room temperature (binding step), then the photosensitizers, Rhodamine B or Rose Bengal, were added to the mixture to a final concentration of 10 or 2.5 µM, respectively (or as indicated in figures). The lamp was then placed on top of the Eppendorf vials and the samples were irradiated with a Euromex Illuminator Eκ-1 lamp (110 WATT, 12 VOLT, halogen lamp LE.5210) coupled with an optical fiber arm (Euromex LE.5214 dual arm light conductor) at room temperature for 60 and 30 minutes (or as indicated in figures), respectively (see Supplementary Figure 1). The power of the lamps was measured using a TES 1335 light meter, equipped with a custom fitting for the lamp bulbs. The light intensity was kept in between 6.8 and 7 KLux. This produces singlet oxygen that activates the furan moiety at the E_{Coil} and allows to evaluate whether other amino acids (Lys, Ser, Cys, His, Tyr, and Trp) at the R_{Coil} can cross-link with the furan warhead. After irradiation, the reaction mixture was left to react for 1 hour at 25°C, and the samples were submitted to HPLC-UV and HPLC-MS analysis, see § 3.1 and 3.2.

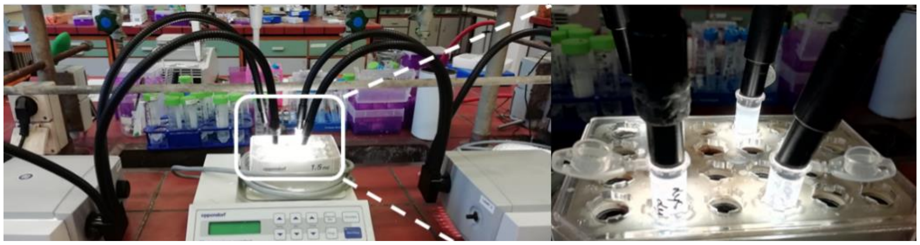

Supplementary Figure 3. The experimental setup for cross-links experiments.

2.10 Circular dichroism (CD) spectroscopy

CD spectra of the E/R$_{Coils}$, as well as a mixture containing both were obtained using a JASCO J7100 instrument (Tokyo, Japan), equipped with a HAAKE cryostat temperature-controlled cell holder at 25 °C. CD spectra are reported as the mean residue molar ellipticity ([θ]) with units of degrees square centimeter per decimole (deg x cm^2/dmol), calculated by the equation:

$$[\theta] = (\theta_{obs} \times MRW) / (10lc)$$

where θ_{obs} is the ellipticity in millidegrees, MRW is the mean residue molecular weight (molecular weight of the peptide divided by the number of amino acid residues), l is the path length of the cuvette in centimetres, and c is the peptide concentration in milligrams per milliliter. The negative molar ellipticity ([θ]) at 222 nm is directly proportional to the amount of helical structure and the $[\theta]_{222}/[\theta]_{208}$ ratio is typically >1.0 for coiled-coil helical dimers and 0.66-0.72 for the single α-helices E3 and K3 coils as previously reported by [7]. The CD spectra were recorded at 50 nm/min scan rate, a bandwidth of 1 nm, a data pitch of 0.1 nm, a response of 0.5 seconds, a wavelength range of 200-260 nm, and a 1 cm path length cell. Each spectrum was an average of nine scans. The final concentration of each peptide in solution was 5 µM in phosphate buffered saline (PBS, pH = 7.4). Baselines were corrected by subtracting the solvent contribution (PBS 1X buffer). The CD spectra for all R$_{Coil}$-Z-8 and E$_{Coil}$-Lys$_{Fur}$-13 were measured at 5 µM for each coil peptide, as well as a 1 to 1 mixture containing both. We decided to work with the minimum concentration that allowed clear visualization of the coiled-coil formation due to the huge amount of coil peptides consumed during CD analysis.

The negative molar ellipticity ([θ]) at 222 nm and the $[\theta]_{222}/[\theta]_{208}$ ratio of the single α-helices E3 (Ac-EIAALEKEIAALEKEIAALEK-NH2) and K3 (Ac-KIAALKEKIAALKEKIAALKE-NH2) coils, as well as a mixture containing both, as previously reported by [7] are below:

	**K$_{Coil}$**	**E$_{Coil}$**	**Coiled-coil**
**$[\theta]_{222}$**	-11320	-9810	-30290
**$[\theta]_{208}$**	-15722	-14864	-28848
**$[\theta]_{222}/[\theta]_{208}$ ratio**	**0.72**	**0,66**	**1,05**

Table 1. The ellipticity ([θ]) at 222 nm and the $[\theta]_{222}/[\theta]_{208}$ ratio of the single α-helices E3 and K3 coils, as well as a mixture containing both.

The E3/K3 coiled-coil complex was designed as a remarkably stable heterodimer with a dissociation constant of 70 nM [7].

If we assume that we have similar K_D values for all R$_{Coil}$-Z-8 with E$_{Coil}$-X$_{Fur}$-13, at a concentration of 5 µM for each coil peptide, the monomer↔dimer equilibrium is shifted toward the formation of coiled-coil dimer in an 88.8%. See calculations below:

In equilibrium:

(1) $[E_{Coil}][R_{Coil}] \leftrightarrow [\text{Coiled-coil}]$

Then the dissociation constant (k_D) is defined as:

(2) $k_D = ([E_{Coil}]\ [R_{Coil}])/[\text{Coiled-coil}]$

where $[E_{Coil}]$, $[R_{Coil}]$, and [Coiled-coil] are the concentrations at equilibrium. The initial concentrations of the reactants are:

(3) $[E_{Coil}]_i = [E_{Coil}] + [\text{Coiled-coil}]$ which can be rearranged as $[E_{Coil}] = [E_{Coil}]_i - [\text{Coiled-coil}]$
(4) $[R_{Coil}]_i = [R_{Coil}] + [\text{Coiled-coil}]$ which can be rearranged as $[R_{Coil}] = [R_{Coil}]_i - [\text{Coiled-coil}]$

Substitute equation 3 and 4 into equation 2:

(5) $k_D = ([E_{Coil}]_i - [\text{Coiled-coil}])\ ([R_{Coil}]_i - [\text{Coiled-coil}])/[\text{Coiled-coil}]$

Rearrange the equation:

(6) $[\text{Coiled-coil}] = (([E_{Coil}]_i - [\text{Coiled-coil}])\ ([R_{Coil}]_i - [\text{Coiled-coil}]))/ k_D$

If [Coiled-coil] is x and the initial concentrations are introduced, the equation is:

(7) $x= ((5-x)\ (5-x))/0.07$; $x= 4.44$ µM

Then the coiled-coil concentration is 4.44 µM, this means that the equilibrium is shifted toward 88.8% of coiled-coil dimer formation. If we work in a range of concentrations between 5 and 25 µM, the ellipticity of the coiled-coil will be very similar, almost independent of peptide concentration.

3 R_{Coil} and E_{Coil} peptides characterization

3.1 Table of the R_{Coil} and E_{Coil} synthesized peptides

Name	Peptide sequence
**R_{Coils}-Z-8**	
**R_{Coil}-Lys-8**	Aba-RIAALRE**K**IAALRERIAALRE-NH_2
**R_{Coil}-Ser-8**	Aba-RIAALRE**S**IAALRERIAALRE-NH_2
**R_{Coil}-Cys-8**	Aba-RIAALRE**C**IAALRERIAALRE-NH_2
**R_{Coil}-His-8**	Aba-RIAALRE**H**IAALRERIAALRE-NH_2
**R_{Coil}-Tyr-8**	Aba-RIAALRE**Y**IAALRERIAALRE-NH_2
**R_{Coil}-Trp-8**	Aba-RIAALRE**W**IAALRERIAALRE-NH_2
**E_{Coil}-X_{Fur}-13**	
**E_{Coil}-Lys_{FUR}-13**	Aba-EIAALEKEIAAL**Lys**$_{Fur}$KEIAALEK-NH_2
**E_{Coil}-Orn_{FUR}-13**	Aba-EIAALEKEIAAL**Orn**$_{Fur}$KEIAALEK-NH_2
**E_{Coil}-Dab_{FUR}-13**	Aba-EIAALEKEIAAL**Dab**$_{Fur}$KEIAALEK-NH_2
**E_{Coil}-Dap_{FUR}-13**	Aba-EIAALEKEIAAL**Dap**$_{Fur}$KEIAALEK-NH_2

Table 2. Peptide sequences of the peptides prepared by standard Fmoc-base solid-phase peptide synthesis. Peptide sequences are written *N*-terminus to *C-terminus.* 4-acetamidobenzoic acid (Aba), Lysine (Lys), Ornithine (Orn), 2,4-diaminobutyric acid (Dab), and 2,3-diaminopropionic acid (Dap).

3.2 R$_{Coil}$-Lys-8 peptide

R$_{Coil}$-Lys-8: Aba-RIAALRE**K**IAALRERIAALRE-NH_2

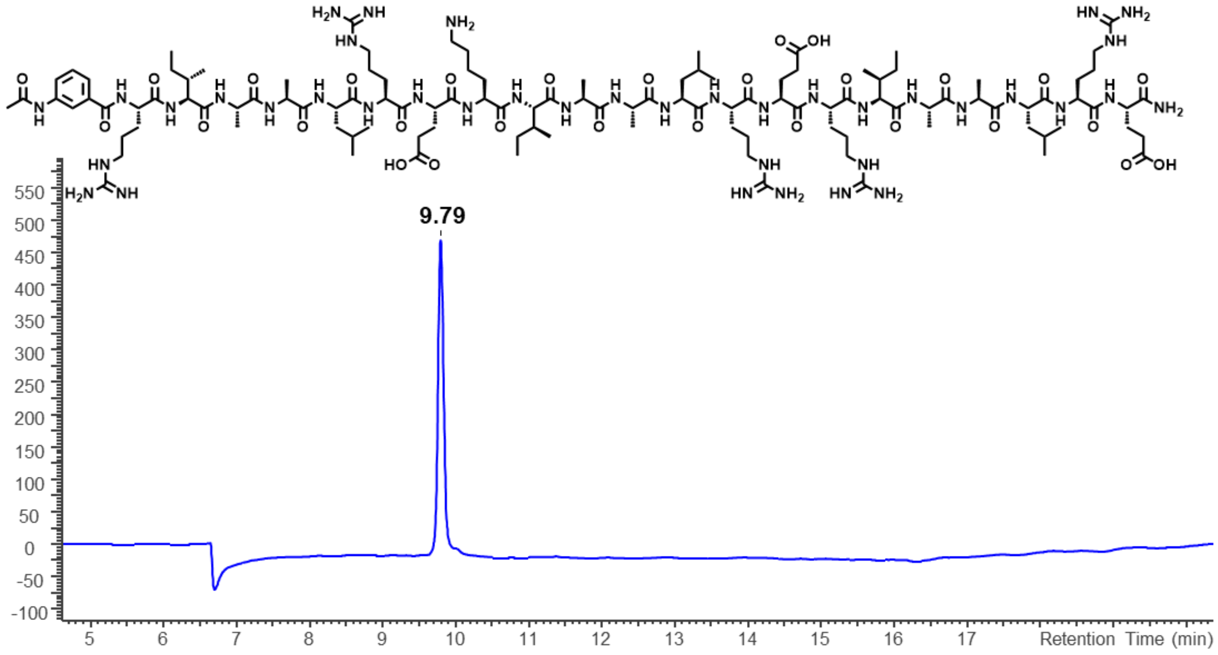

Column: XTerra® Shield RP18, 125Å (5 μM 2.1 x 250 mm).

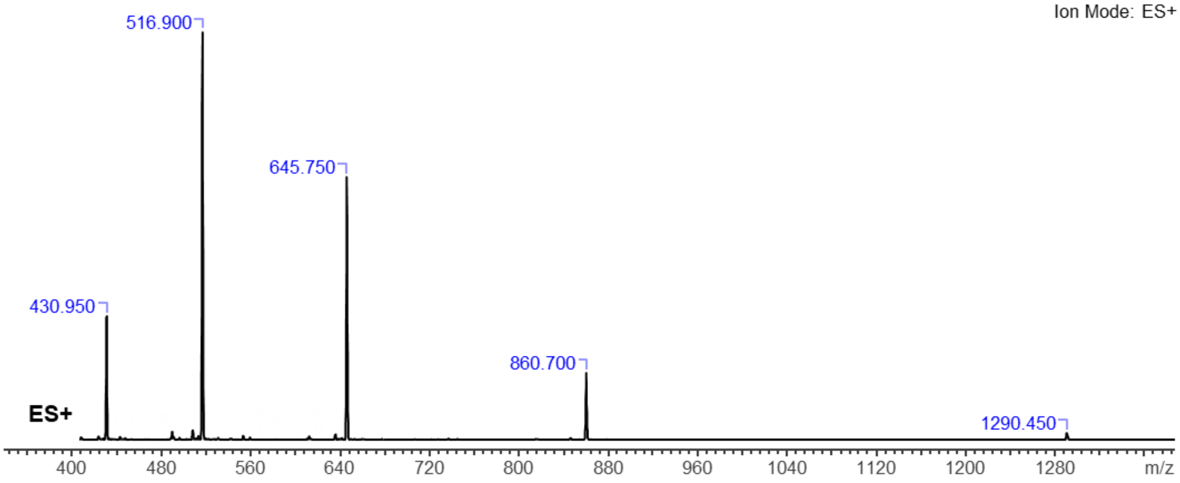

LC-ESI-MS m/z at t_r= 9.79: $[M+2H]^{2+}/2$= 1290.45; $[M+3H]^{3+}/3$= 860.70; $[M+4H]^{4+}/4$= 645.75; $[M+5H]^{5+}/5$= 516.90; $[M+6H]^{6+}/6$= 430.95.

m/z calculated for $C_{114}H_{199}N_{39}O_{29}$ $[M+1H]^{+}$: 2578.53 Da; found 2579.06 Da.

Supplementary Figure 4. HPLC-MS spectrum of R$_{Coil}$-Lys-8. HPLC-UV trace at 254 nm (top) and MS spectrum of the corresponding peak (bottom) with the calculated mass of the peptide.

3.3 E_{Coil}-Orn_{Fur}-13 peptide

E_{Coil}-Orn_{Fur}-13: Aba-EIAALEKEIAAL**<u>Orn</u>**$_{Fur}$KEIAALEK-NH_2

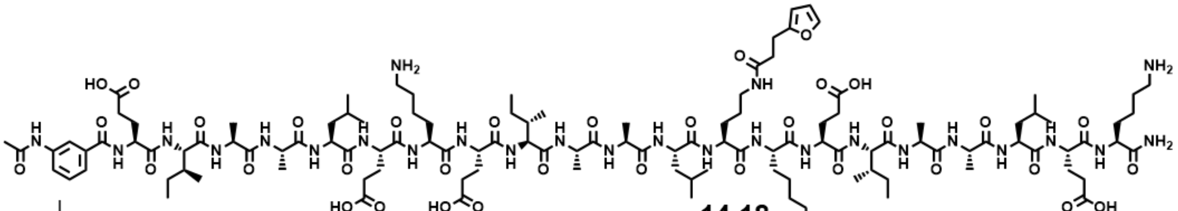

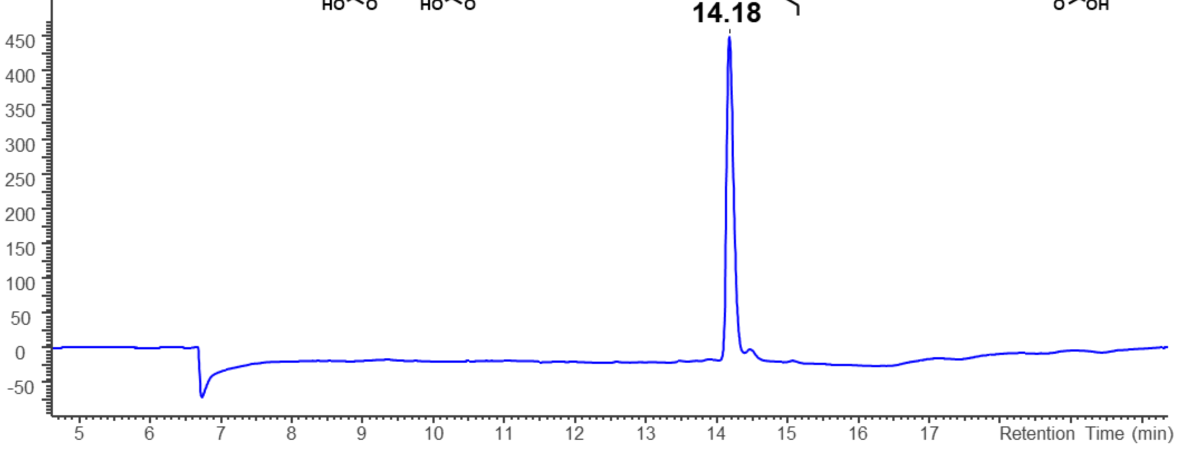

Column: XTerra® Shield RP18, 125Å (5 µM 2.1 x 250 mm).

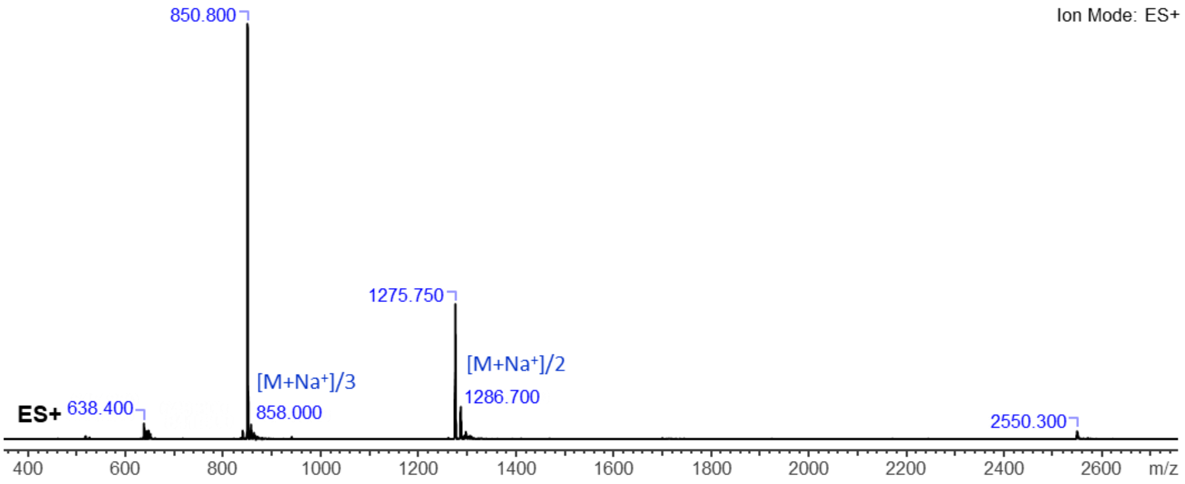

LC-ESI-MS m/z at t_r= 14.18: $[M+1H]^{1+}/1$= 2550.30; $[M+2H]^{2+}/2$= 1275.75; $[M+3H]^{3+}/3$= 850.80; $[M+4H]^{4+}/4$= 638.40.

m/z calculated for $C_{118}H_{193}N_{27}O_{35}$ $[M+1H]^+$: 2548.42 Da; found 2549.70 Da.

Supplementary Figure 5. HPLC-MS spectrum of E_{Coil}-Lys_{Fur}-13. HPLC-UV trace at 260 nm (top) and MS spectrum of the corresponding peak (bottom) with the calculated mass of the peptide.

3.4 E_{Coil}-Lys_{Fur}-13 peptide

E_{Coil}-Lys_{Fur}-13: Aba-EIAALEKEIAAL**<u>Lys</u>**$_{Fur}$KEIAALEK-NH_2

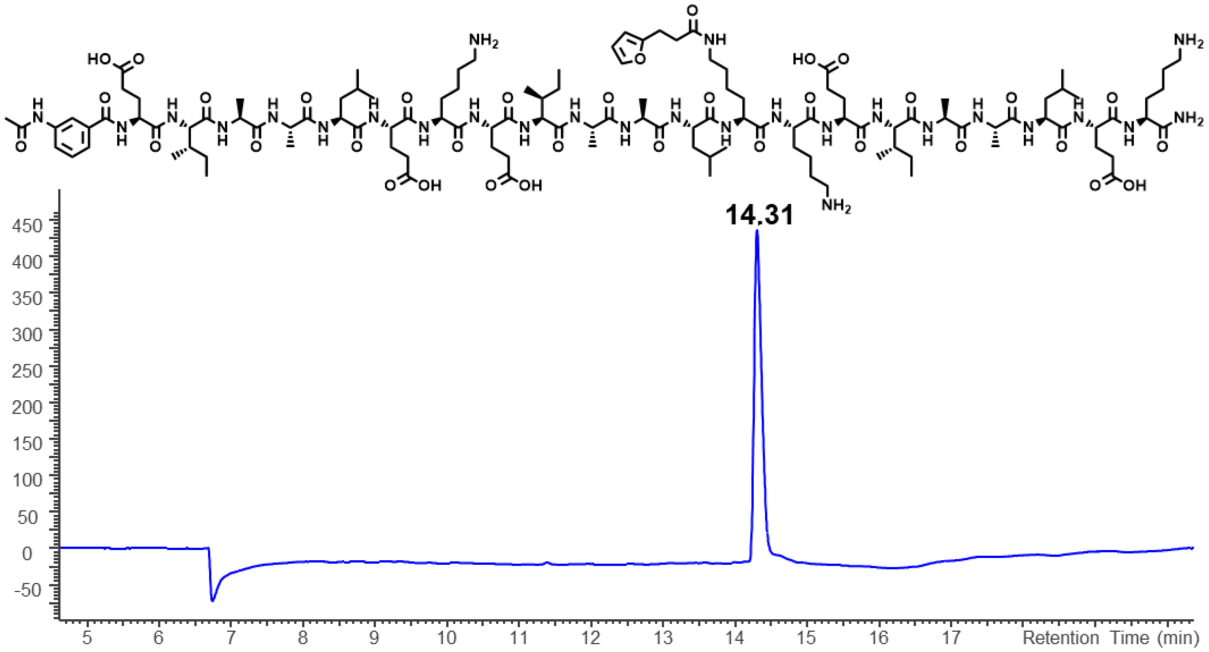

Column: XTerra® Shield RP18, 125Å (5 µM 2.1 x 250 mm).

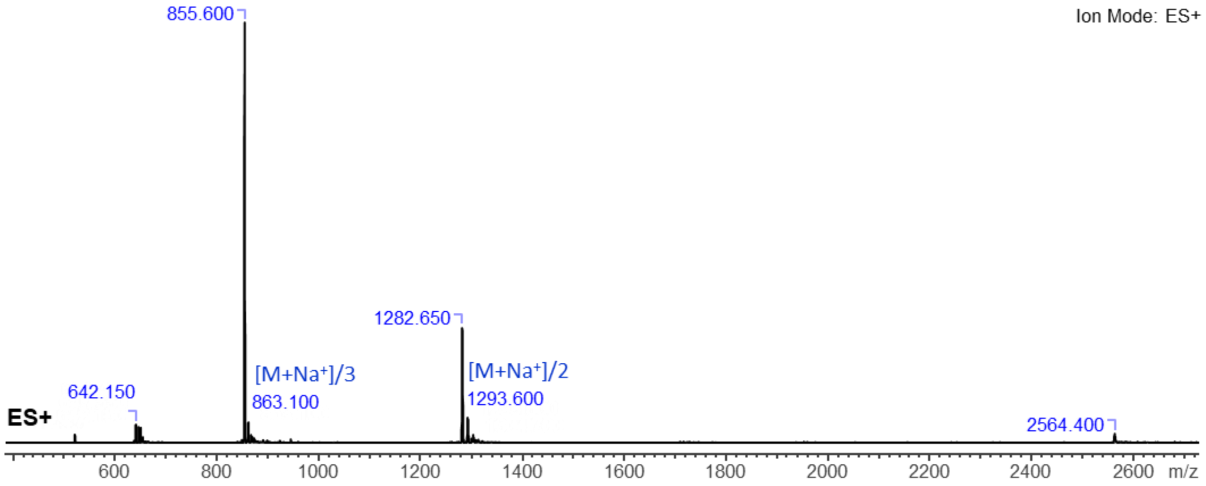

LC-ESI-MS m/z at t_r= 14.31: $[M+1H]^{1+}/1$= 2564.40; $[M+2H]^{2+}/2$= 1282.65; $[M+3H]^{3+}/3$= 855.60; $[M+4H]^{4+}/4$= 642.15.

m/z calculated for $C_{119}H_{195}N_{27}O_{35}$ $[M+1H]^+$: 2562.43 Da; found 2563.68 Da.

Supplementary Figure 6. HPLC-MS spectrum of E_{Coil}-Lys_{Fur}-13. HPLC-UV trace at 260 nm (top) and MS spectrum of the corresponding peak (bottom) with the calculated mass of the peptide.

3.5 E_{Coil}-Dab_{Fur}-13 peptide

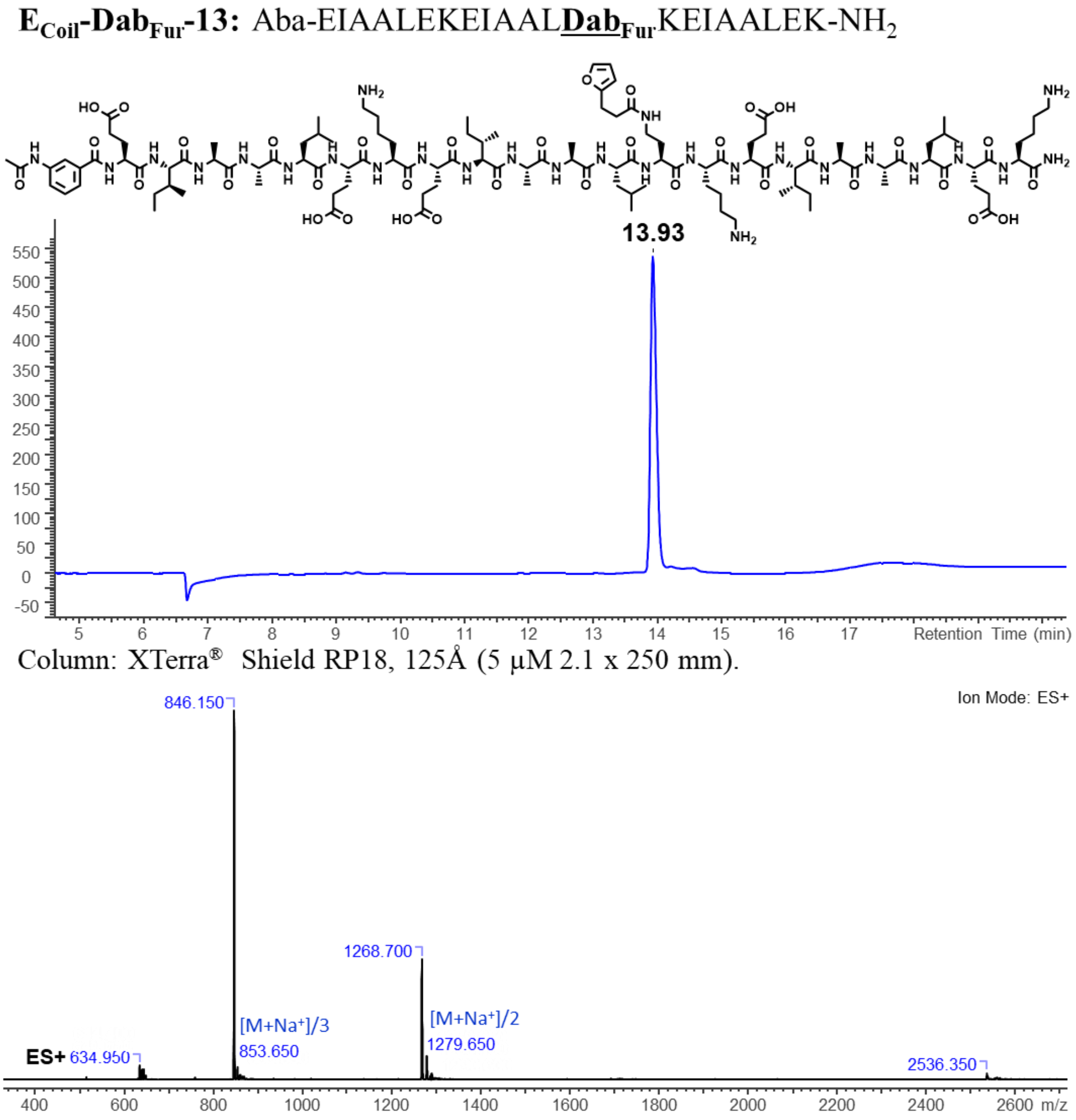

LC-ESI-MS m/z at t_r= 13.93: $[M+1H]^{1+}/1$= 2536.35; $[M+2H]^{2+}/2$= 1268.70; $[M+3H]^{3+}/3$= 846.15; $[M+4H]^{4+}/4$= 634.95.

m/z calculated for $C_{117}H_{191}N_{27}O_{35}$ $[M+1H]^{+}$: 2534.40 Da; found 2535.67 Da.

Supplementary Figure 7. HPLC-MS spectrum of ECoil-DabFur-13. HPLC-UV trace at 260 nm (top) and MS spectrum of the corresponding peak (bottom) with the calculated mass of the peptide.

3.6 E_{Coil}-Dap_{Fur}-13 peptide

E_{Coil}-Dap_{Fur}-13: Aba-EIAALEKEIAAL**<u>Dap</u>**$_{Fur}$KEIAALEK-NH_2

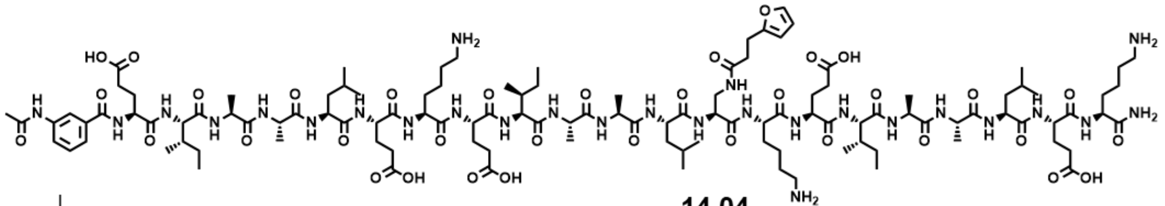

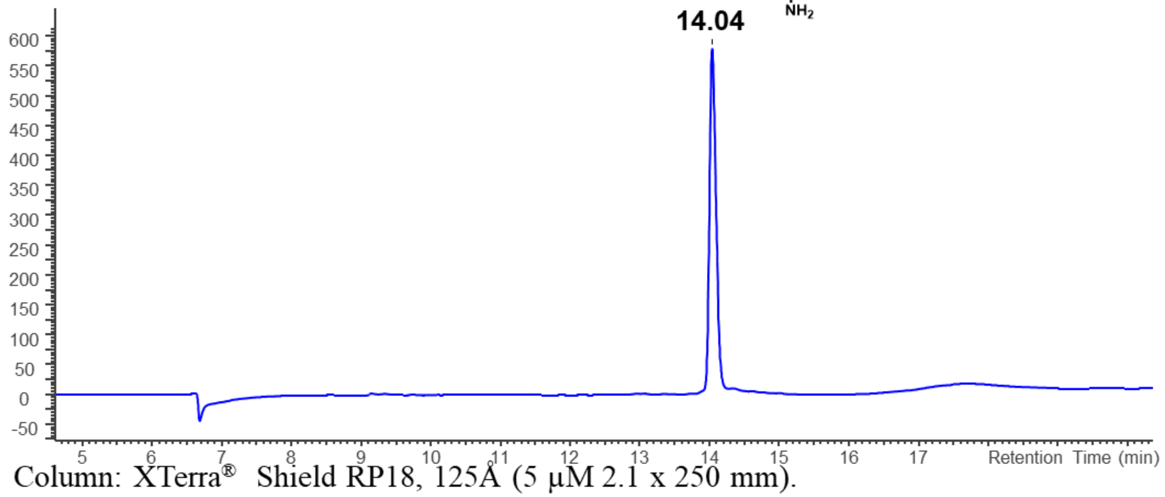

Column: XTerra® Shield RP18, 125Å (5 µM 2.1 x 250 mm).

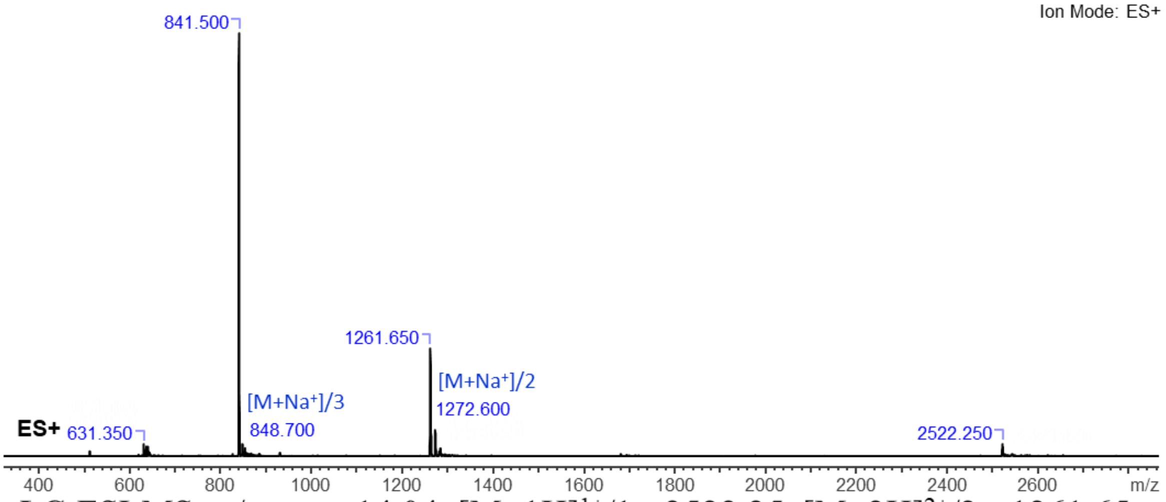

LC-ESI-MS m/z at t_r= 14.04: $[M+1H]^{1+}/1$= 2522.25; $[M+2H]^{2+}/2$= 1261.65; $[M+3H]^{3+}/3$= 841.50; $[M+4H]^{4+}/4$= 631.35.

m/z calculated for $C_{116}H_{189}N_{27}O_{35}$ $[M+1H]^{+}$: 2520.38 Da; found 2521.61 Da.

Supplementary Figure 8. HPLC-MS spectrum of E_{Coil}-Dap_{Fur}-13. HPLC-UV trace at 260 nm (top) and MS spectrum of the corresponding peak (bottom) with the calculated mass of the peptide.

3.7 R_{Coil}-Ser-8 peptide

R_{Coil}-Ser-8: Aba-RIAALRE<u>S</u>IAALRERIAALRE-NH_2

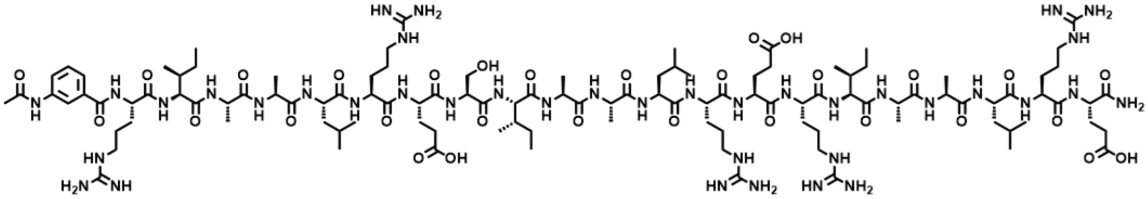

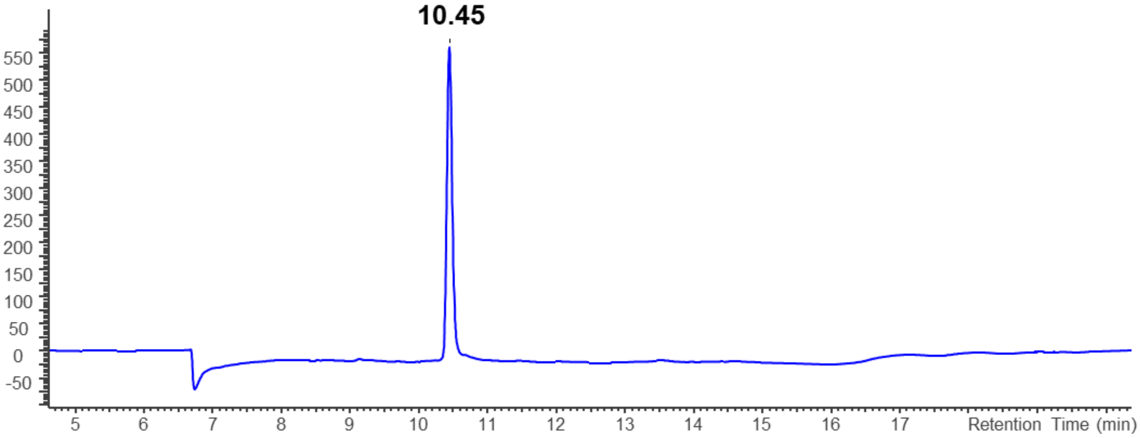

Column: XTerra® Shield RP18, 125Å (5 µM 2.1 x 250 mm).

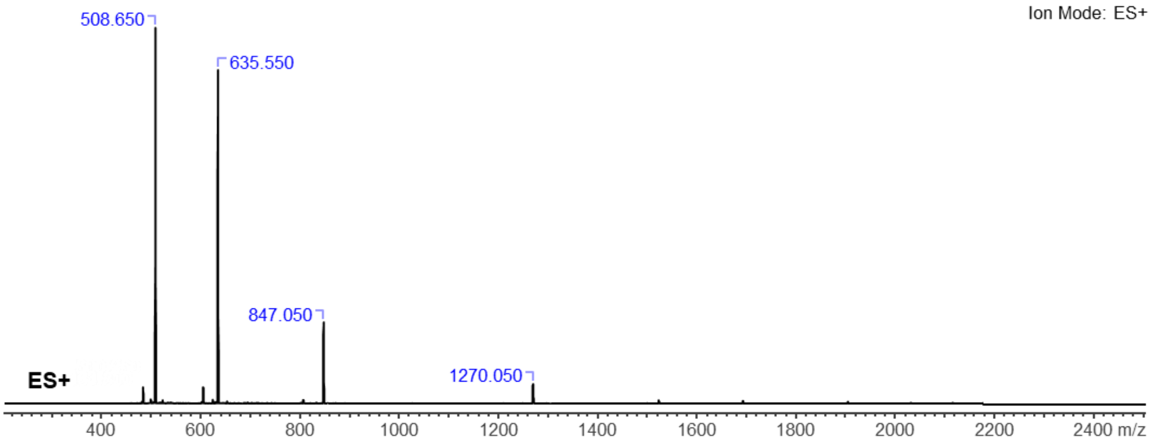

LC-ESI-MS m/z at t_r= 10.45: $[M+2H]^{2+}/2$= 1270.05; $[M+3H]^{3+}/3$= 847.05; $[M+4H]^{4+}/4$= 635.55; $[M+5H]^{5+}/5$= 508.65.

m/z calculated for $C_{111}H_{192}N_{38}O_{30}$ $[M+1H]^{+}$: 2537.47 Da; found 2539.06 Da.

Supplementary Figure 9. HPLC-MS spectrum of R_{Coil}-Ser-8. HPLC-UV trace at 260 nm (top) and MS spectrum of the corresponding peak (bottom) with the calculated mass of the peptide.

3.8 R$_{Coil}$-Cys-8 peptide

R$_{Coil}$-Cys-8: Aba-RIAALRE<u>C</u>IAALRERIAALRE-NH_2

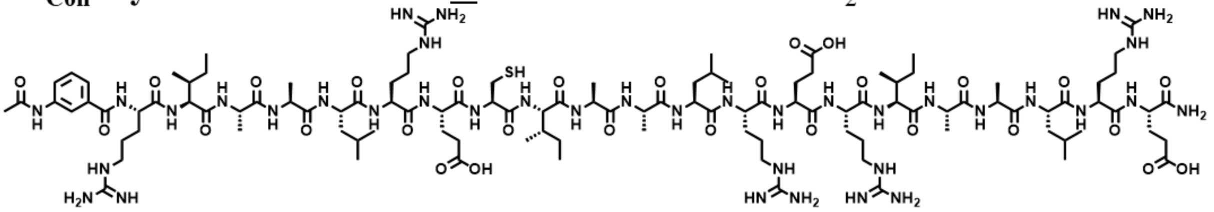

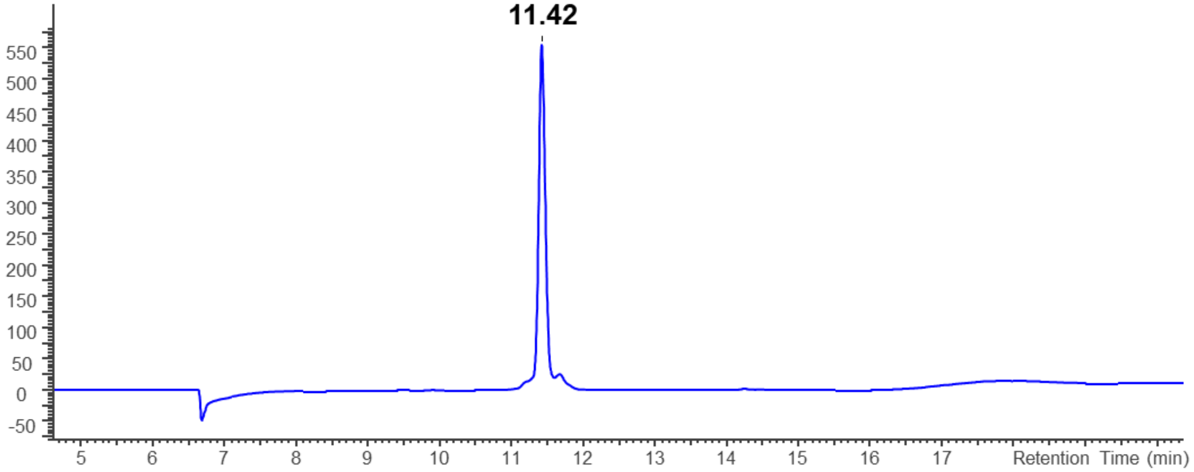

Column: XTerra® Shield RP18, 125Å (5 µM 2.1 x 250 mm).

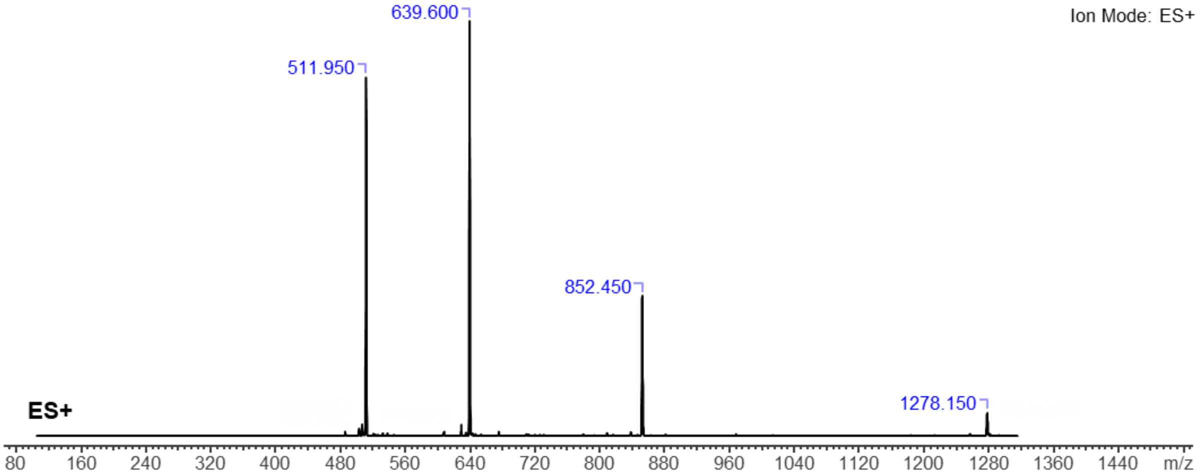

LC-ESI-MS m/z at t_r= 11.42: $[M+2H]^{2+}/2$= 1278.15; $[M+3H]^{3+}/3$= 852.45; $[M+4H]^{4+}/4$= 639.6; $[M+5H]^{5+}/5$= 511.95.

m/z calculated for $C_{111}H_{192}N_{38}O_{29}S$ $[M+1H]^+$: 2553.44 Da; found 2554.3 Da.

Supplementary Figure 10. HPLC-MS spectrum of R$_{Coil}$-Cys-8. HPLC-UV trace at 260 nm (top) and MS spectrum of the corresponding peak (bottom) with the calculated mass of the peptide**.**

3.9 R$_{Coil}$-His-8 peptide

R$_{Coil}$-His-8: Aba-RIAALRE**H**IAALRERIAALRE-NH_2

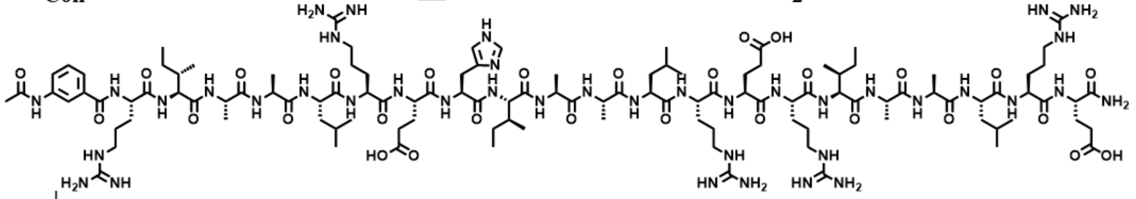

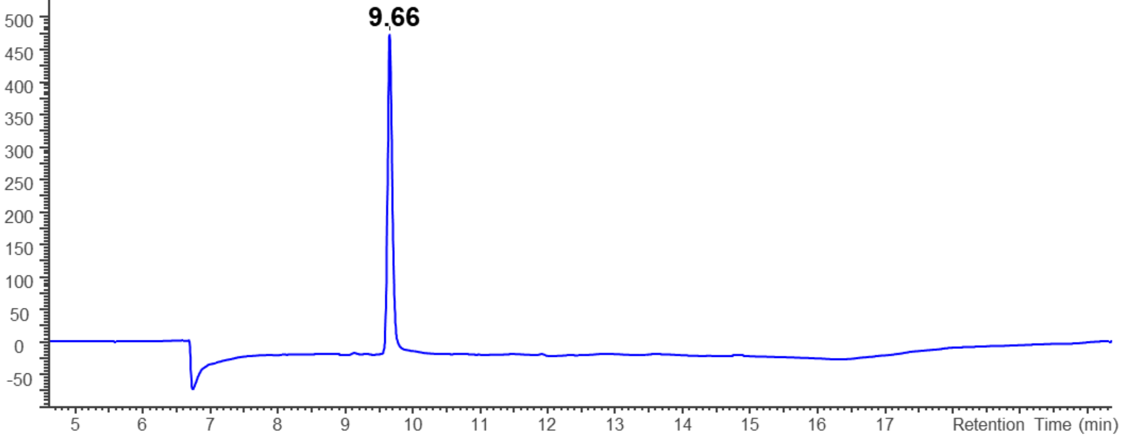

Column: XTerra® Shield RP18, 125Å (5 µM 2.1 x 250 mm).

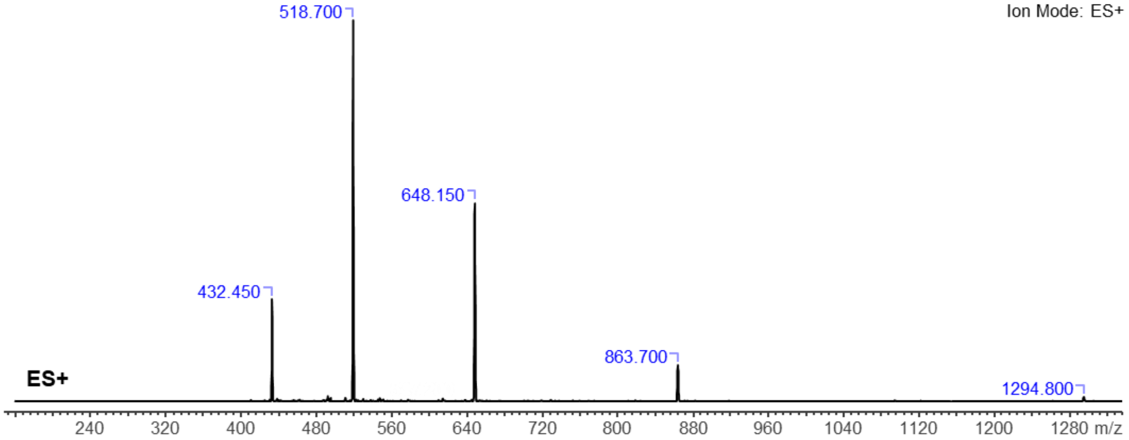

LC-ESI-MS m/z at t_r= 9.66: $[M+2H]^{2+}/2$= 1294.80; $[M+3H]^{3+}/3$= 863.70; $[M+4H]^{4+}/4$= 648.15; $[M+5H]^{5+}/5$= 518.70; $[M+6H]^{6+}/6$= 432.45.

m/z calculated for $C_{114}H_{194}N_{40}O_{29}$ $[M+1H]^{+}$: 2587.49 Da; found 2589.09 Da.

Supplementary Figure 11. HPLC-MS spectrum of R$_{Coil}$-His-8. HPLC-UV trace at 260 nm (top) and MS spectrum of the corresponding peak (bottom) with the calculated mass of the peptide.

3.10 R_{Coil}-Tyr-8 peptide

R_{Coil}-Tyr-8: Aba-RIAALRE<u>Y</u>IAALRERIAALRE-NH_2

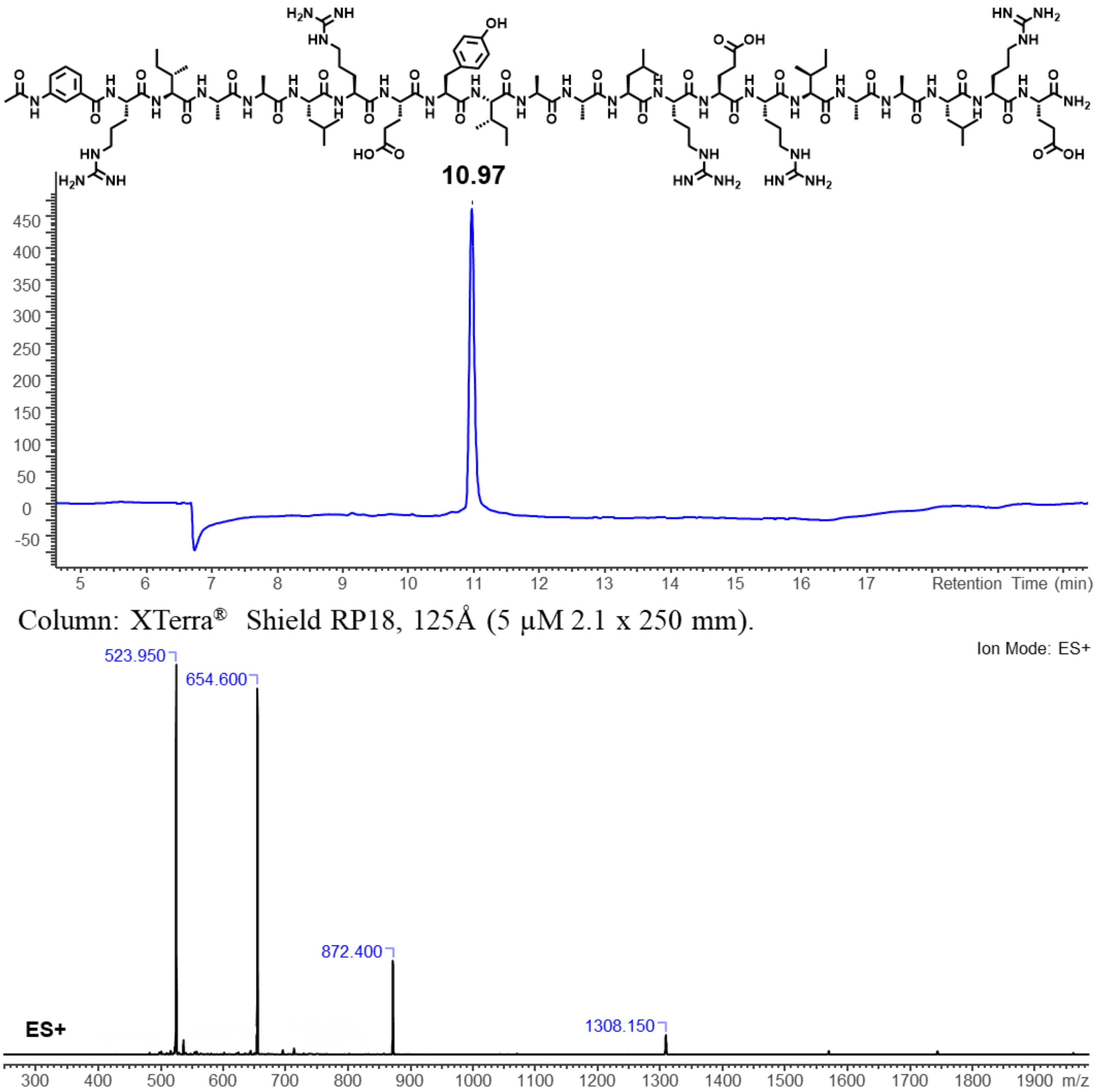

LC-ESI-MS m/z at t_r= 10.97: $[M+2H]^{2+}/2$= 1308.15; $[M+3H]^{3+}/3$= 872.40; $[M+4H]^{4+}/4$= 654.60; $[M+5H]^{5+}/5$= 523.95.

m/z calculated for $C_{117}H_{196}N_{38}O_{30}$ $[M+1H]^+$: 2613.50 Da; found 2615.18. Da.

Supplementary Figure 12. HPLC-MS spectrum of R_{Coil}-Tyr-8. HPLC-UV trace at 260 nm (top) and MS spectrum of the corresponding peak (bottom) with the calculated mass of the peptide.

3.11 R_{Coil}-Trp-8 peptide

R_{Coil}-Trp-8: Aba-RIAALRE<u>**W**</u>IAALRERIAALRE-NH_2

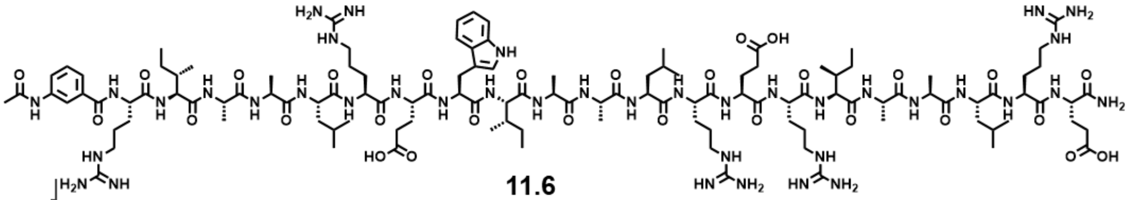

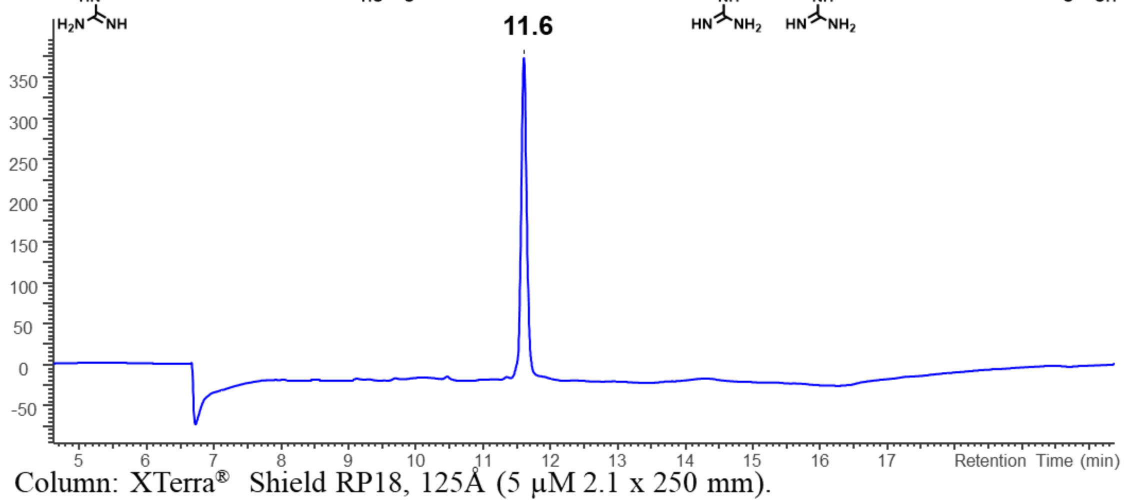

Column: XTerra® Shield RP18, 125Å (5 µM 2.1 x 250 mm).

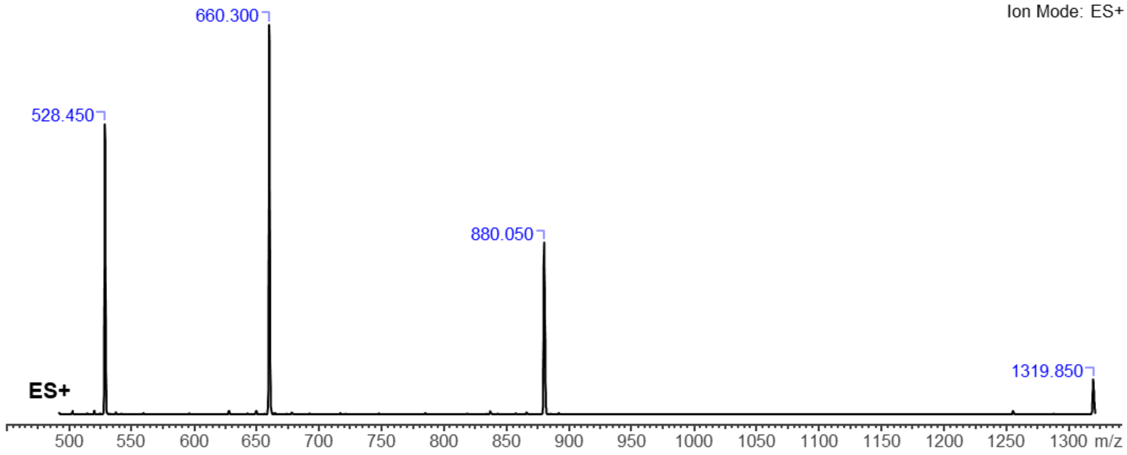

LC-ESI-MS m/z at t_r= 11.6: $[M+2H]^{2+}/2$= 1319.85; $[M+3H]^{3+}/3$= 880.05; $[M+4H]^{4+}/4$= 660.30; $[M+5H]^{5+}/5$= 528.45.

m/z calculated for $C_{119}H_{197}N_{39}O_{29}$ $[M+1H]^{+}$: 2636.51 Da; found 2638.09 Da.

Supplementary Figure 13. HPLC-MS spectrum of R_{Coil}-Trp-8. HPLC-UV trace at 260 nm (top) and MS spectrum of the corresponding peak (bottom) with the calculated mass of the peptide

4 R_{Coil}-Lys-8 cross-links with E_{Coil}-Orn_{Fur}-13

4.1 RB as a photosensitizer

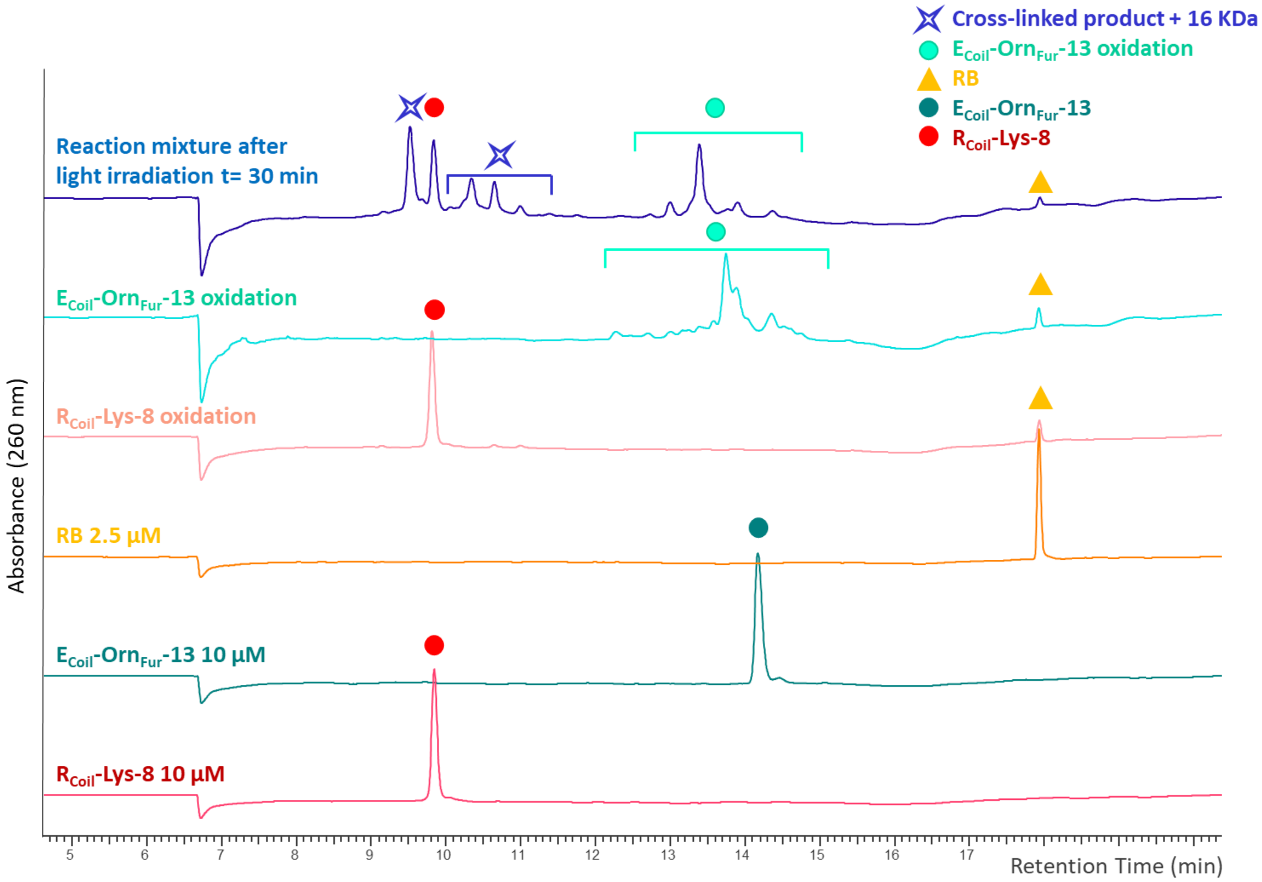

Supplementary Figure 14. HPLC-UV chromatograms recorded at 260 nm with a XTerra® Shield RP18 column, 125Å (5µM 2,1 x 250mm). The reaction mixture after light irradiation (blue trace) is the cross-link reaction between R_{Coil}-Lys-8 (red trace) and E_{Coil}-Orn_{Fur}-13 (dark green trace) after 30 minutes of light irradiation with RB (orange trace) at 2.5 µM. The R_{Coil}-Lys-8 oxidation (light red) trace and E_{Coil}-Orn_{Fur}-13 oxidation (green) trace were generated by exposure to singlet oxygen by light irradiation in the presence of RB at 2.5 µM for 30 minutes in absence of the other Coil

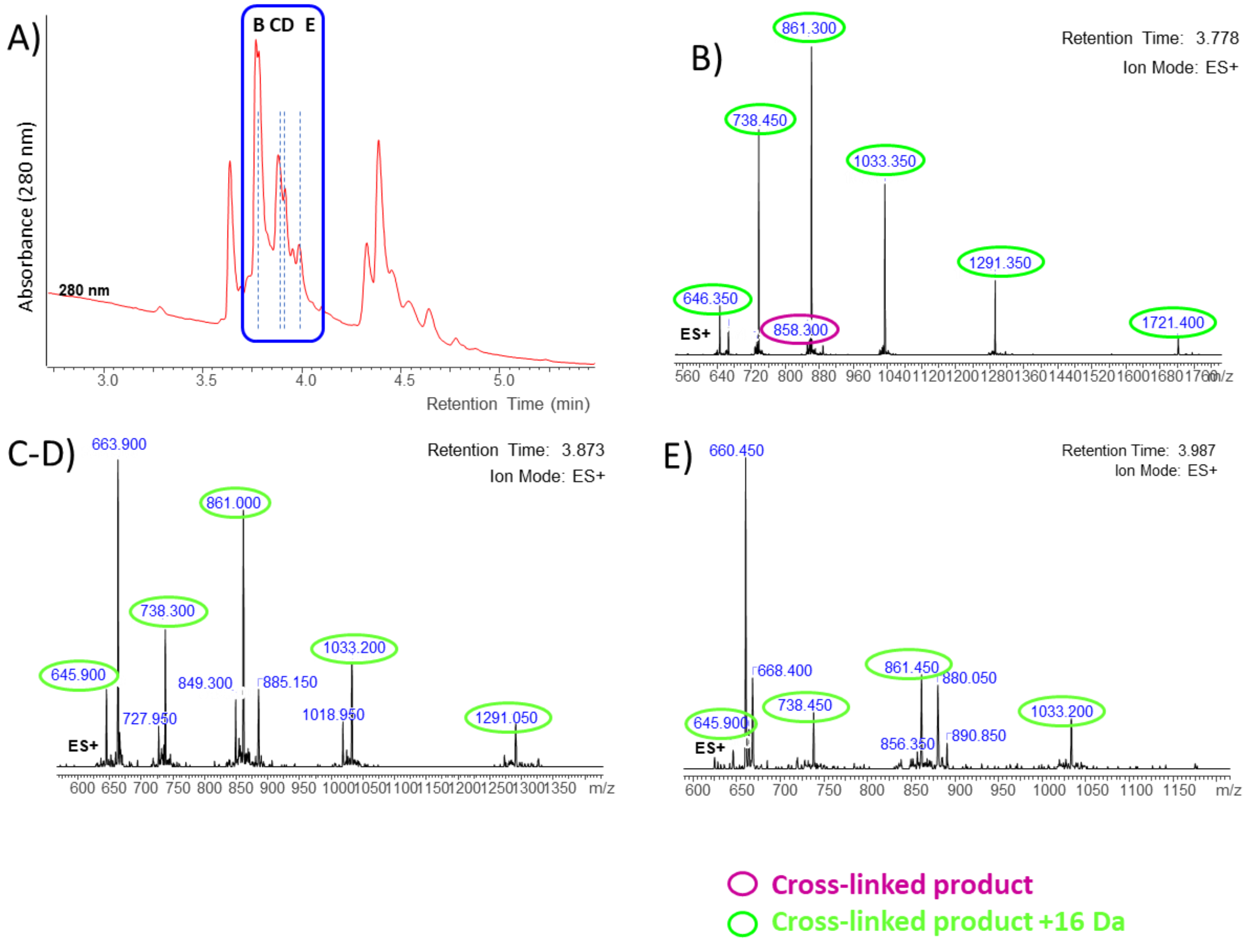

Supplementary Figure 15. A) HPLC-UV chromatogram recorded at 280 nm with the column Phenomenex Kinetex EVO C18 100 Å (150 x 4.6 mm, 5 µm, at 35 °C). B-E) ESI-MS spectrum of the peaks at 3.778, 3.873, and 3.987 minutes corresponding to the cross-linked products formed between R_{Coil}-Lys-8 and E_{Coil}-Orn_{Fur}-13 (blue rectangle in A). The green circles correspond to the mass-to-charge ratio of ions of the oxidation of the cross-linked product (+16 Da): $[M+3H]^{3+}/3$= 1721.4; $[M+4H]^{4+}/4$= 1291.4; $[M+5H]^{5+}/5$= 1033.4; $[M+6H]^{6+}/6$= 861.3; $[M+7H]^{7+}/7$= 738.5, $[M+7H]^{7+}/7$= 646.4. Exact mass (m/z) and molecular weight (MW) calculated for the oxidation of the cross-linked product $C_{232}H_{392}N_{66}O_{66}$ $[M+1H]^{+}$: 5158.9 Da and 5162.0; found 5161.7 Da.

4.2 Rhd B as a photosensitizer

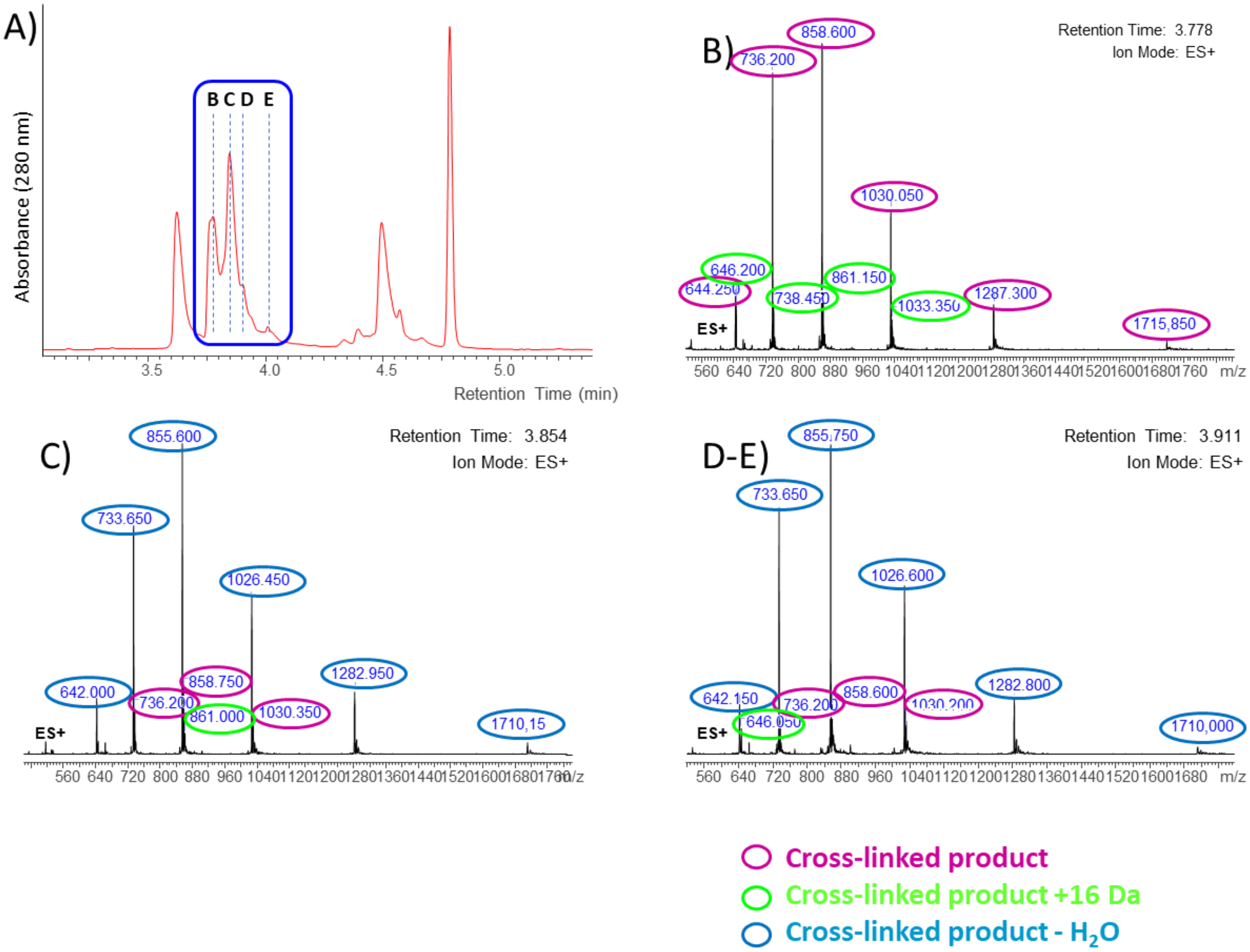

Supplementary Figure 16. A) HPLC-UV chromatogram recorded at 280 nm with the column Phenomenex Kinetex EVO C18 100 Å (150 x 4.6 mm, 5 µm, at 35 °C). B-E) ESI-MS spectrum of the peaks at 3.778, 3.854, and 3.911 minutes corresponding to the cross-linked products formed between R_{Coil}-Lys-8 and E_{Coil}-Orn$_{Fur}$-13 (blue rectangle in A). The purple circles correspond to the mass-to-charge ratio of ions of the cross-linked product: $[M+3H]^{3+}/3$= 1715.9; $[M+4H]^{4+}/4$= 1287.3; $[M+5H]^{5+}/5$= 1030.1; $[M+6H]^{6+}/6$= 858.6; $[M+7H]^{7+}/7$= 736.2, $[M+7H]^{7+}/7$= 644.2. Exact mass (m/z) and molecular weight (MW) calculated for $C_{232}H_{392}N_{66}O_{65}$ $[M+1H]^{+}$: 5142.9 Da and 5146.0; found 5145.9 Da. The blue circles correspond to the dehydrated (-18 Da) form of the cross-linked product: $[M+3H]^{3+}/3$= 1710.1; $[M+4H]^{4+}/4$= 1282.9; $[M+5H]^{5+}/5$= 1026.5; $[M+6H]^{6+}/6$= 855.6; $[M+7H]^{7+}/7$= 733.7, $[M+7H]^{7+}/7$= 642.2. Exact mass (m/z) and molecular weight (MW) calculated for $C_{232}H_{390}N_{66}O_{64}$ $[M+1H]^{+}$: 5124.9 Da and 5128.0; found 5128.1 Da. The green circles correspond to the oxidation of the cross-linked product (+16 Da respectively).

5 Optimization of cross-linking conditions for Lys

5.1 How to calculate the percentage of cross-linked product area

To perform quantitative analysis of the cross-link yield to be able to compare different experiments, since the ratio between photosensitizer concentration and coil peptide concentration differs between experiments, as well as the ratio between E_{Coil} and R_{Coil} peptide concentration, we decided to calculate the percentage of cross-linked product area base on the area under the peak of the R_{Coil} (marked by a red dot on the HPLC chromatogram) and the area under the cross-linked products peaks (marked by a purple star on the HPLC chromatogram). The total area considered is the area of the R_{Coil} peak plus the area of the cross-linked product. The ratio between the cross-linked product (XL) area and the total area, multiplied by 100, will provide us with a percentage of the cross-linked product area. The area of the R_{Coil} oxidation peak (marked by a light red dot on the HPLC chromatogram) and the area of the R_{Coil}-Cys-8 dimerization peak (marked by a blue square on the HPLC chromatogram) were also included in the total area calculation for cross-linking experiments with R_{Coil}-Tyr-8 and R_{Coil}-Cys-8, respectively.

5.2 Quantitative analysis of the cross-link yield for Figure 5 in the manuscript

	Peak Area (Y units/ms)	% XL area
**Trace D**		
R_{Coil}-Lys-8	826629	
**Cross-linked product**	3215234	**79,5**
Total area	4041863	
**Trace C**		
R_{Coil}-Lys-8	2183440	
**Cross-linked product**	5102676	**70,0**
Total area	7286116	
**Trace B**		
R_{Coil}-Lys-8	1784653	
**Cross-linked product**	2521354	**58,6**
Total area	4306008	
**Trace A**		
R_{Coil}-Lys-8	1227447	
**Cross-linked product**	1800289	**59,5**
Total area	3027735	

Table 3. The peak area was calculated integrating the peak in the HPLC chromatogram. The percentage of the cross-linked product (XL) area is the ratio between the XL area and the total area, multiplied by 100. The total area considered is the area of the R_{Coil}-Lys-8 peak plus the area of the cross-linked product.

5.3 Cross-linked product of R_{Coil}-Lys-8 with E_{Coil}-Lys_{Fur}-13

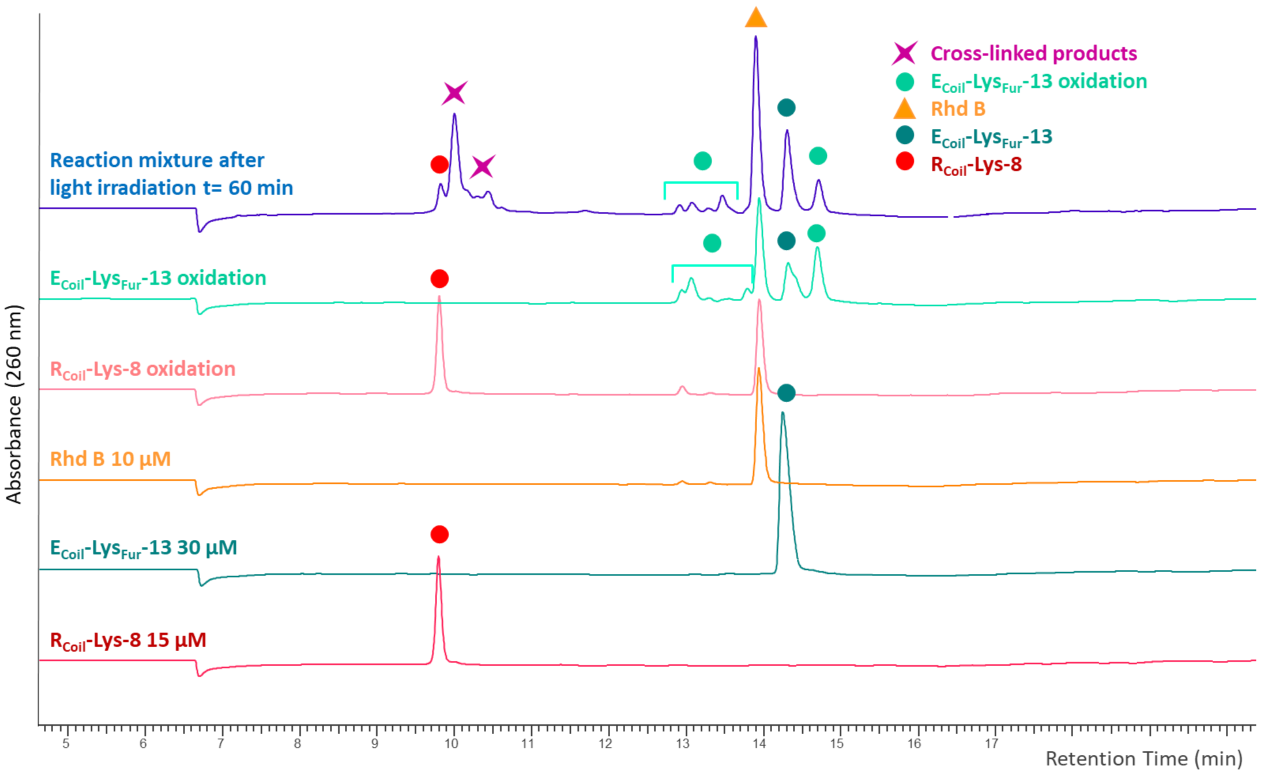

	Peak Area (Y units/ms)	*% XL area*
R_{Coil}-Lys-8	532143	
Cross-linked product	3609519	**87,2**
Total area	4141661	

Supplementary Figure 17. HPLC-UV chromatograms recorded at 260 nm with a XTerra® Shield RP18 column, 125Å (5µM 2,1 x 250mm). The reaction mixture after light irradiation (blue trace) is the cross-link reaction between R_{Coil}-Lys-8 (red trace) and E_{Coil}-Lys_{Fur}-13 (dark green trace) after 60 minutes of light irradiation with Rhd B (orange trace) at 10 µM. The R_{Coil}-Lys-8 oxidation (light red) trace and E_{Coil}-Lys_{Fur}-13 oxidation (green) trace were generated by exposure to singlet oxygen by light irradiation in the presence of Rhd B at 10 µM for 60 minutes in absence of the other Coil. The cross-link yield was quantified as a percentage of the cross-linked product area and the values are indicated in the Table.

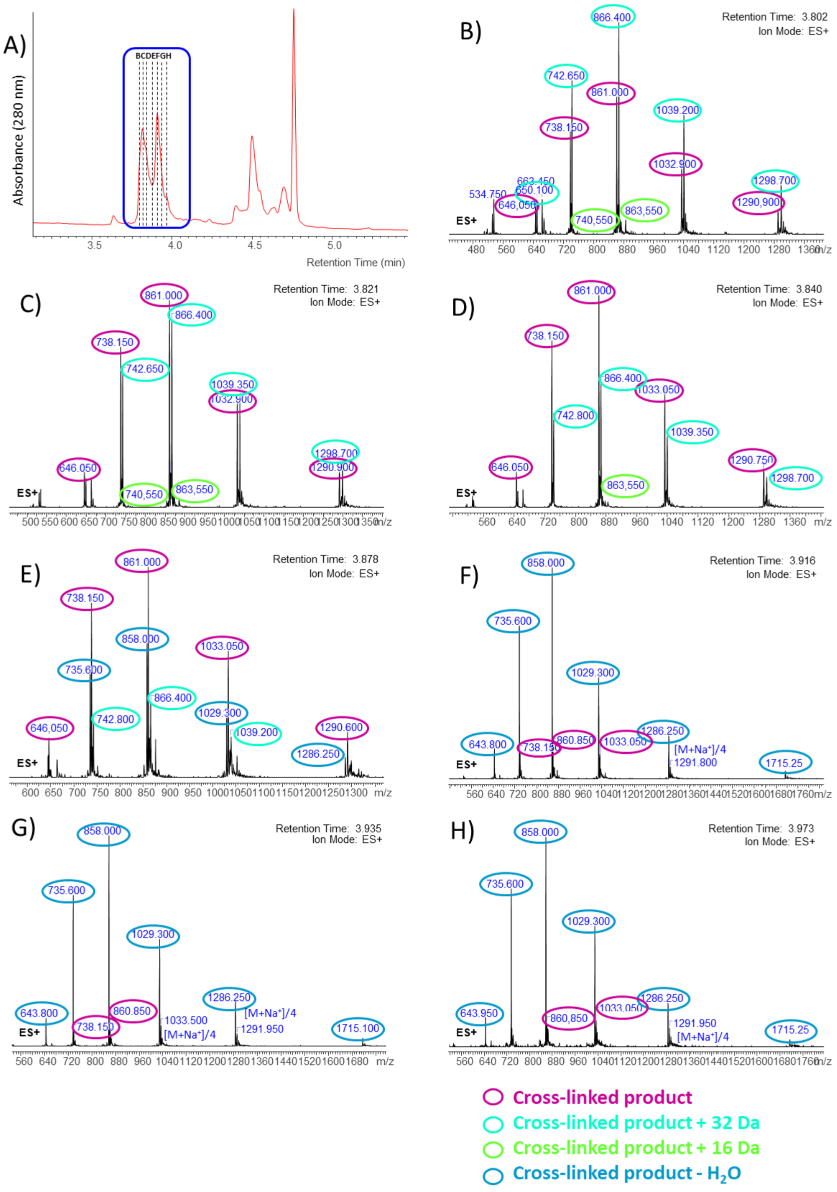

Supplementary Figure 18. A) HPLC-UV chromatogram recorded at 280 nm with the column Phenomenex Kinetex EVO C18 100 Å (150 x 4.6 mm, 5 µm, at 35 °C). B-E) ESI-MS spectrum of the peaks at 3.802, 3.821, 3.840, 3.878, 3.916, 3.935, and 3.973 minutes corresponding to the cross-linked products formed between R_{Coil}-Lys-8 and E_{Coil}-Lys_{Fur}-13 (blue rectangle in A). The purple circles correspond to the mass-to-charge ratio of ions of the cross-linked product: $[M+4H]^{4+}/4$= 1290.9; $[M+5H]^{5+}/5$= 1032.9; $[M+6H]^{6+}/6$= 861.0; $[M+7H]^{7+}/7$= 738.2, $[M+7H]^{7+}/7$= 646.1. Exact mass (m/z) and molecular weight (MW) calculated for $C_{233}H_{394}N_{66}O_{65}$ $[M+1H]^{+}$: 5156.9 Da and 5160.0; found 5159.9 Da. The blue circles correspond to the dehydrated (-18 Da) form of the cross-linked product: $[M+3H]^{3+}/3$= 1715.1; $[M+4H]^{4+}/4$= 1286.3; $[M+5H]^{5+}/5$= 1029.3; $[M+6H]^{6+}/6$= 858.0; $[M+7H]^{7+}/7$= 735.6, $[M+7H]^{7+}/7$= 643.8. Exact mass (m/z) and molecular weight (MW) calculated for $C_{233}H_{392}N_{66}O_{64}$ $[M+1H]^{+}$: 5138.9 Da and 5142.0; found 5141.9 Da. The green and the light blue circles correspond to the oxidation of the cross-linked product (+16 and +32 Da, respectively).

5.4 Cross-linked product of R_{Coil}-Lys-8 with E_{Coil}-Orn$_{Fur}$-13

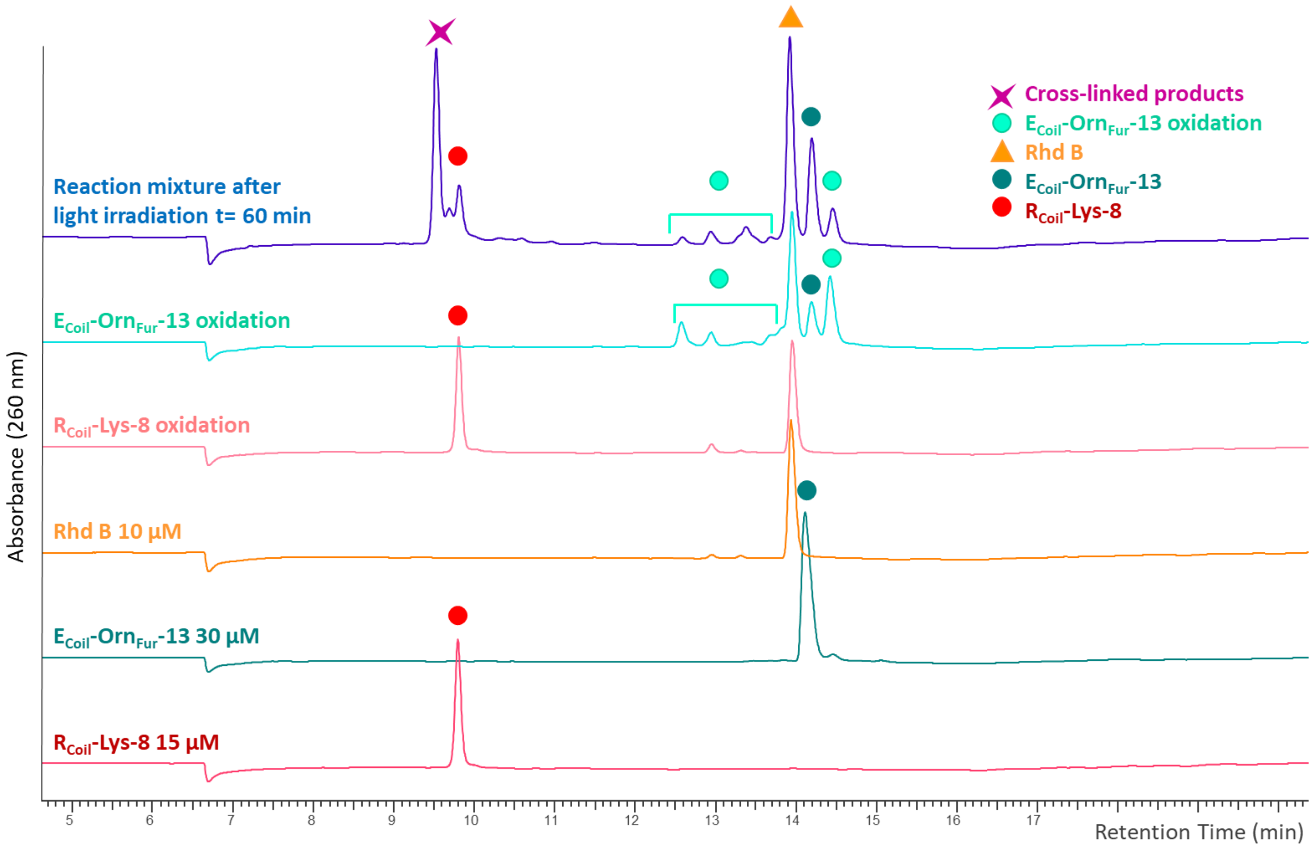

	Peak Area (Y units/ms)	*% XL area*
R_{Coil}-Lys-8	826629	
Cross-linked product	3215234	**79.5**
Total area	4041863	

Supplementary Figure 19. HPLC-UV chromatograms recorded at 260 nm with a XTerra® Shield RP18 column, 125Å (5µM 2,1 x 250mm). The reaction mixture after light irradiation (blue trace) is the cross-link reaction between R_{Coil}-Lys-8 (red trace) and E_{Coil}-Orn$_{Fur}$-13 (dark green trace) after 60 minutes of light irradiation with Rhd B (orange trace) at 10 µM. The R_{Coil}-Lys-8 oxidation (light red) trace and E_{Coil}-Orn$_{Fur}$-13 oxidation (green) trace were generated by exposure to singlet oxygen by light irradiation in the presence of Rhd B at 10 µM for 60 minutes in absence of the other Coil. The cross-link yield was quantified as a percentage of the cross-linked product area and the values are indicated in the Table.

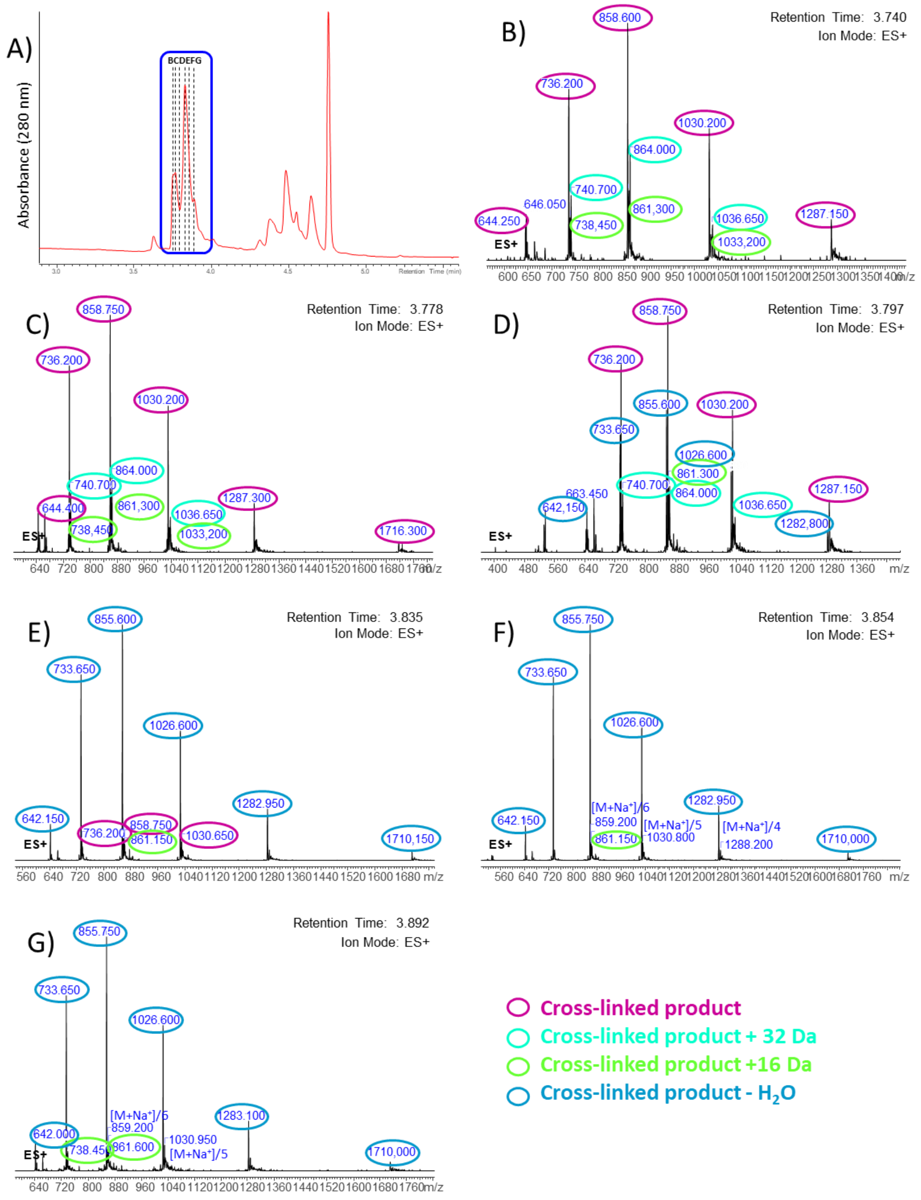

Supplementary Figure 20. A) HPLC-UV chromatogram recorded at 280 nm with the column Phenomenex Kinetex EVO C18 100 Å (150 x 4.6 mm, 5 µm, at 35 °C). B-E) ESI-MS spectrum of the peaks at 3.740, 3.778, 3.797, 3.835, 3.854, and 3.892 minutes corresponding to the cross-linked products formed between R_{Coil}-Lys-8 and E_{Coil}-Orn_{Fur}-13 (blue rectangle in A). The purple circles correspond to the mass-to-charge ratio of ions of the cross-linked product: $[M+3H]^{3+}$/= 1716.3, $[M+4H]^{4+}$/4= 1287.3; $[M+5H]^{5+}$/5= 1030.2; $[M+6H]^{6+}$/6= 858.6; $[M+7H]^{7+}$/7= 736.2, $[M+7H]^{7+}$/7= 644.2. Exact mass (m/z) and molecular weight (MW) calculated for $C_{232}H_{392}N_{66}O_{65}$ $[M+1H]^{+}$: 5142.9 Da and 5146.0; found 5145.9 Da. The blue circles correspond to the dehydrated (-18 Da) form of the cross-linked product: $[M+3H]^{3+}$/3= 1710.1; $[M+4H]^{4+}$/4= 1282.9; $[M+5H]^{5+}$/5= 1026.6; $[M+6H]^{6+}$/6= 855.6; $[M+7H]^{7+}$/7= 733.7, $[M+7H]^{7+}$/7= 642.2. Exact mass (m/z) and molecular weight (MW) calculated for $C_{232}H_{390}N_{66}O_{64}$ $[M+1H]^{+}$: 5124.9 Da and 5128.0; found 5128.1 Da. The green and light blue circles correspond to the oxidation of the cross-linked product (+16, +32 Da respectively).

5.5 Cross-linked product of R_{Coil}-Lys-8 with E_{Coil}-Dab_{Fur}-13

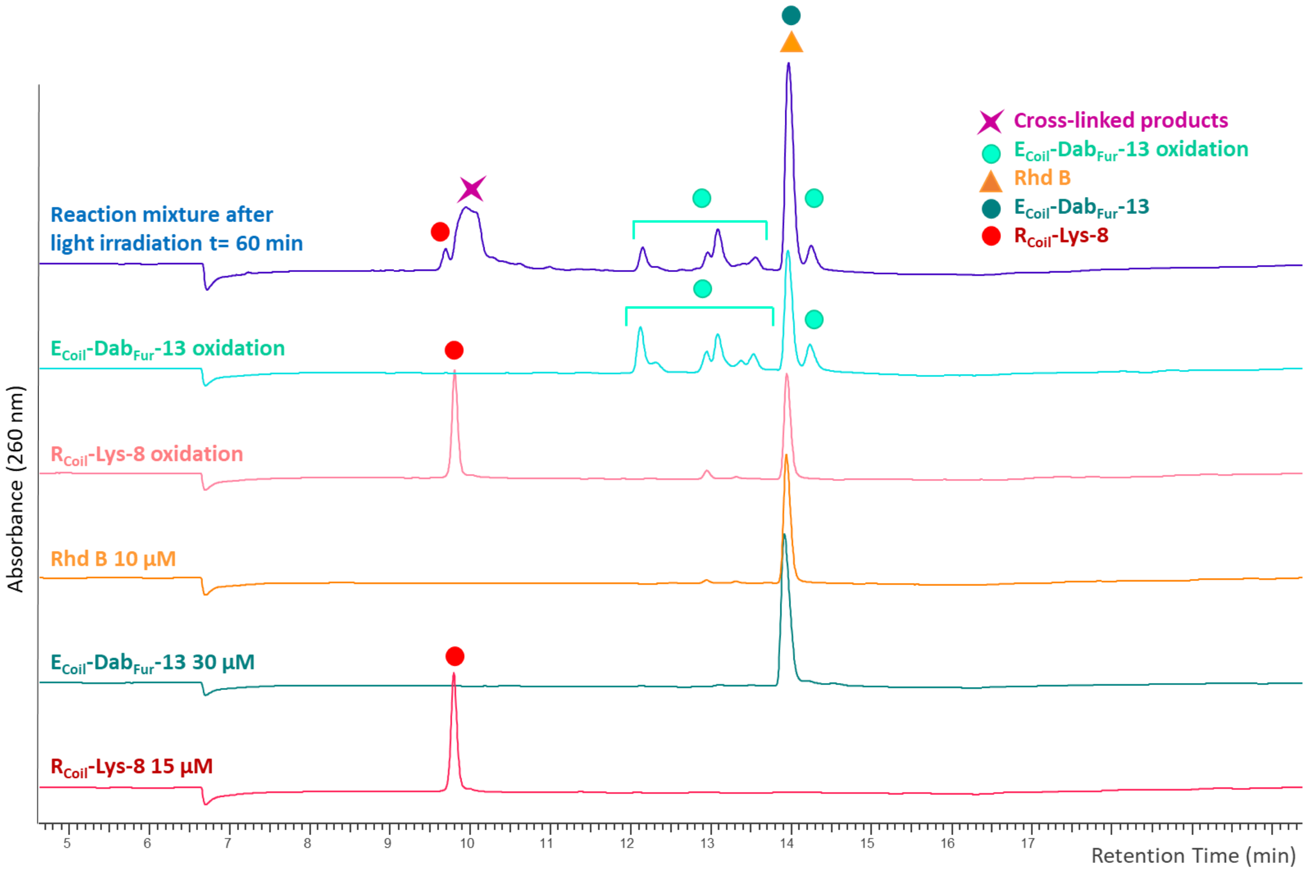

	Peak Area (Y units/ms)	*% XL area*
R_{Coil}-Lys-8	370293	
Cross-linked product	4311979	**92.1**
Total area	4682272	

Supplementary Figure 21. HPLC-UV chromatograms recorded at 260 nm with a XTerra® Shield RP18 column, 125Å (5µM 2,1 x 250mm). The reaction mixture after light irradiation (blue trace) is the cross-link reaction between R_{Coil}-Lys-8 (red trace) and E_{Coil}-Dab_{Fur}-13 (dark green trace) after 60 minutes of light irradiation with Rhd B (orange trace) at 10 µM. The R_{Coil}-Lys-8 oxidation (light red) trace and E_{Coil}-Dab_{Fur}-13 oxidation (green) trace were generated by exposure to singlet oxygen by light irradiation in the presence of Rhd B at 10 µM for 60 minutes in absence of the other Coil. The cross-link yield was quantified as a percentage of the cross-linked product area and the values are indicated in the Table.

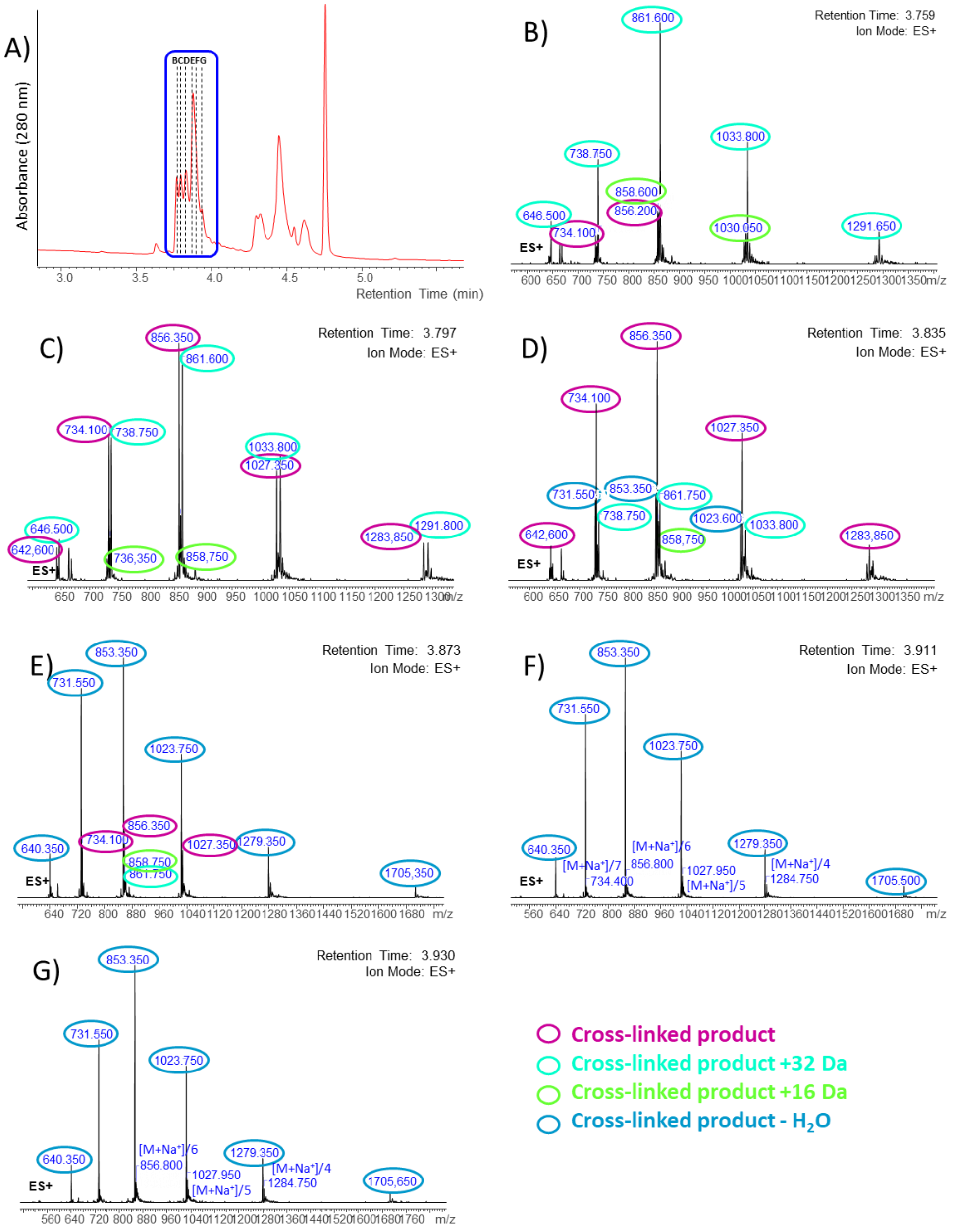

Supplementary Figure 22. A) HPLC-UV chromatogram recorded at 280 nm with the column Phenomenex Kinetex EVO C18 100 Å (150 x 4.6 mm, 5 µm, at 35 °C). B-E) ESI-MS spectrum of the peaks at 3.759, 3.797, 3.835, 3.873, 3.911, and 3.930 minutes corresponding to the cross-linked products formed between R_{Coil}-Lys-8 and E_{Coil}-Dab_{Fur}-13 (blue rectangle in A). The purple circles correspond to the mass-to-charge ratio of ions of the cross-linked product: $[M+4H]^{4+}/4$= 1283.8; $[M+5H]^{5+}/5$= 1027.3; $[M+6H]^{6+}/6$= 856.3; $[M+7H]^{7+}/7$= 734.1, $[M+7H]^{7+}/7$= 642.6. Exact mass (m/z) and molecular weight (MW) calculated for $C_{231}H_{390}N_{66}O_{65}$ $[M+1H]^{+}$: 5128.9 Da and 5131.9; found 5131.5 Da. The blue circles correspond to the dehydrated (-18 Da) form of the cross-linked product: $[M+3H]^{3+}/3$= 1705.5; $[M+4H]^{4+}/4$= 1279.3; $[M+5H]^{5+}/5$= 1023.8; $[M+6H]^{6+}/6$= 853.3; $[M+7H]^{7+}/7$= 731.5, $[M+7H]^{7+}/7$= 640.3. Exact mass (m/z) and molecular weight (MW) calculated for $C_{231}H_{388}N_{66}O_{64}$ $[M+1H]^{+}$: 5110.9 Da and 5113.9; found 5113.9 Da. The green and light blue circles correspond to the oxidation of the cross-linked product (+16 and +32 Da, respectively).

5.6 Cross-linked product of R$_{Coil}$-Lys-8 with E$_{Coil}$-Dap$_{Fur}$-13

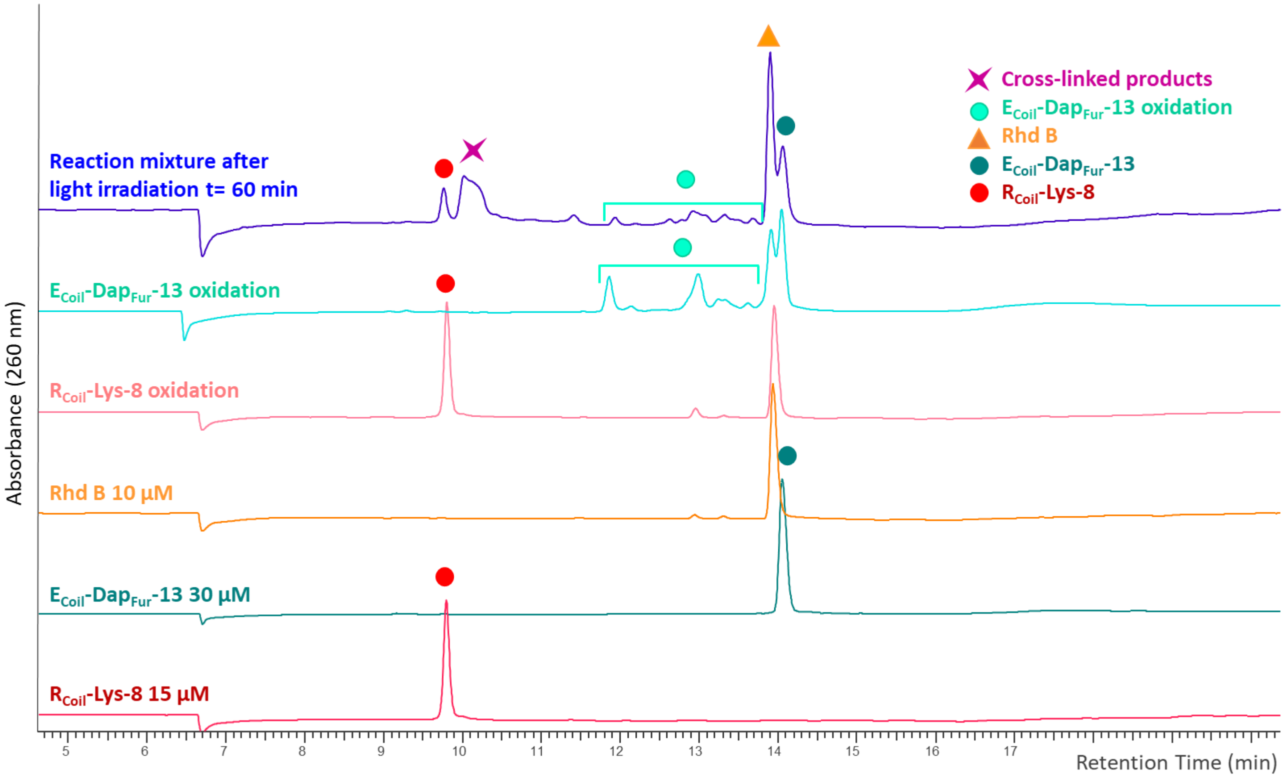

	Peak Area (Y units/ms)	*Area %*
R$_{Coil}$-Lys-8	293155	
Cross-linked product	1364747	**82.3**
Total area	1657902	

Supplementary Figure 23. HPLC-UV chromatograms recorded at 260 nm with a XTerra® Shield RP18 column, 125Å (5µM 2,1 x 250mm). The reaction mixture after light irradiation (blue trace) is the cross-link reaction between R$_{Coil}$-Lys-8 (red trace) and E$_{Coil}$-Dap$_{Fur}$-13 (dark green trace) after 60 minutes of light irradiation with Rhd B (orange trace) at 10 µM. The R$_{Coil}$-Lys-8 oxidation (light red) trace and E$_{Coil}$-Dap$_{Fur}$-13 oxidation (green) trace were generated by exposure to singlet oxygen by light irradiation in the presence of Rhd B at 10 µM for 60 minutes in absence of the other Coil. The cross-link yield was quantified as a percentage of the cross-linked product area and the values are indicated in the Table.

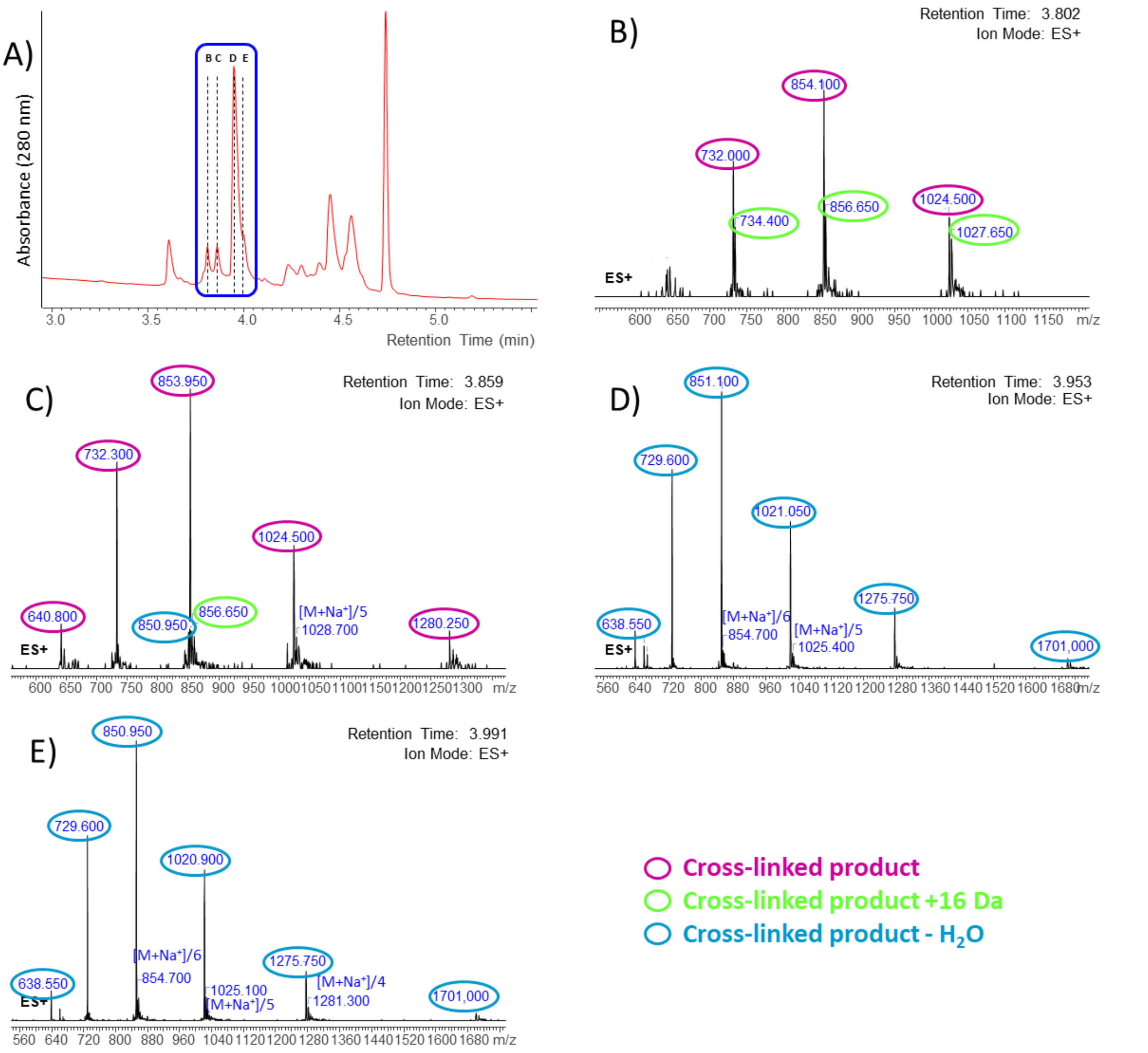

Supplementary Figure 24. A) HPLC-UV chromatogram recorded at 280 nm with the column Phenomenex Kinetex EVO C18 100 Å (150 x 4.6 mm, 5 µm, at 35 °C). B-E) ESI-MS spectrum of the peaks at 3.802, 3.859, 3.953, and 3.911 minutes corresponding to the cross-linked products formed between R_{Coil}-Lys-8 and E_{Coil}-Dap_{Fur}-13 (blue rectangle in A). The purple circles correspond to the mass-to-charge ratio of ions of the cross-linked product: $[M+4H]^{4+}/4$= 1280.2; $[M+5H]^{5+}/5$= 1024.5; $[M+6H]^{6+}/6$= 854.1; $[M+7H]^{7+}/7$= 732.2, $[M+7H]^{7+}/7$= 640.8. Exact mass (m/z) and molecular weight (MW) calculated for $C_{230}H_{388}N_{66}O_{65}$ $[M+1H]^{+}$: 5114.9 Da and 5117.9; found 5118.0 Da. The blue circles correspond to the dehydrated (-18 Da) form of the cross-linked product: $[M+4H]^{4+}/4$= 1275.8; $[M+5H]^{5+}/5$= 1021.0; $[M+6H]^{6+}/6$= 851.1; $[M+7H]^{7+}/7$= 729.6, $[M+7H]^{7+}/7$= 638.5. Exact mass (m/z) and molecular weight (MW) calculated for $C_{230}H_{386}N_{66}O_{64}$ $[M+1H]^{+}$: 5096.9 Da and 5099.9; found 5100.0 Da. The green circles correspond to the oxidation of the cross-linked product (+16 Da).

5.7 CD spectroscopy of R_{Coil}-Lys-8 and E_{Coil}-Dap_{Fur}-13

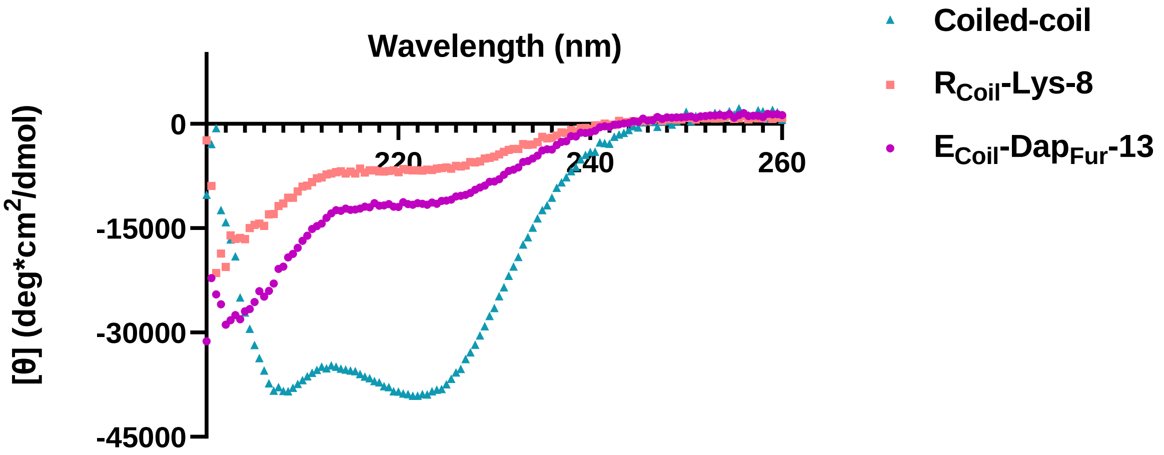

	R_{Coil}-Lys-8	E_{Coil}-Dap_{Fur}-13	Coiled-coil
$[\theta]_{222}$	-12204,27	-11427,43	-39089,62
$[\theta]_{208}$	-16766,48	-20562,57	-38439,24
**$[\theta]_{222}/[\theta]_{208}$ ratio**	**0,73**	**0,56**	**1,02**

Supplementary Figure 25. CD spectra obtained for the R_{Coil}-Lys-8 (light red), the E_{Coil}-Dap_{Fur}-13 (purple), and the E/R coiled-coil equimolar mixture (green) in PBS at 5 µM**.**

5.8 CD spectroscopy of R_{Coil}-Lys-8 and E_{Coil}-Lys_{Fur}-13

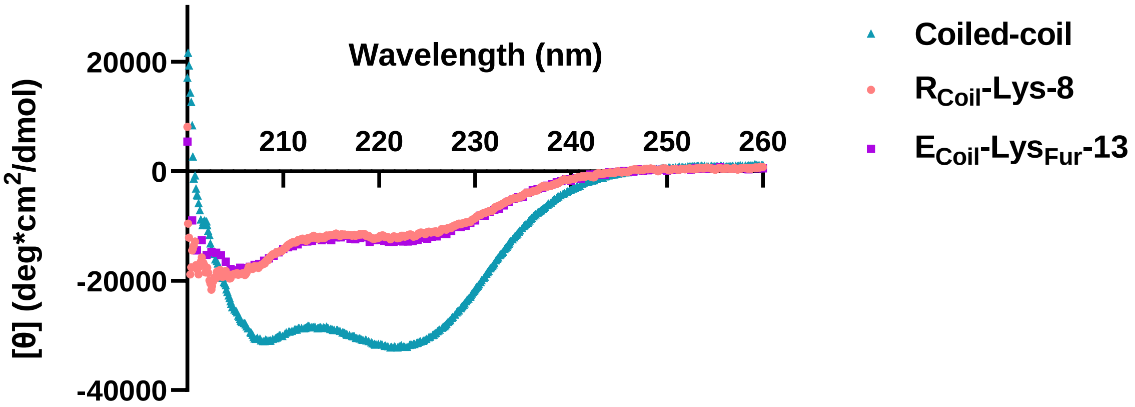

	R_{Coil}-Lys-8	E_{Coil}-Lys_{Fur}-13	Coiled-coil
**$[\theta]_{222}$**	-12204,27	-12733,78	-32107,04
**$[\theta]_{208}$**	-16766,48	-16355,18	-31080,48
**$[\theta]_{222}/[\theta]_{208}$ ratio**	**0,73**	**0,78**	**1,03**

Supplementary Figure 26. CD spectra obtained for the R_{Coil}-Lys-8 (light red), the E_{Coil}-Lys_{Fur}-13 (purple), and the E/R coiled-coil equimolar mixture (green) in PBS at 5 µM**.**

6 CD spectroscopy of R_{Coils} with E_{Coil}-Lys_{Fur}-13 peptide

6.1 CD spectroscopy of R_{Coil}-Ser-8 and E_{Coil}-Lys_{Fur}-13

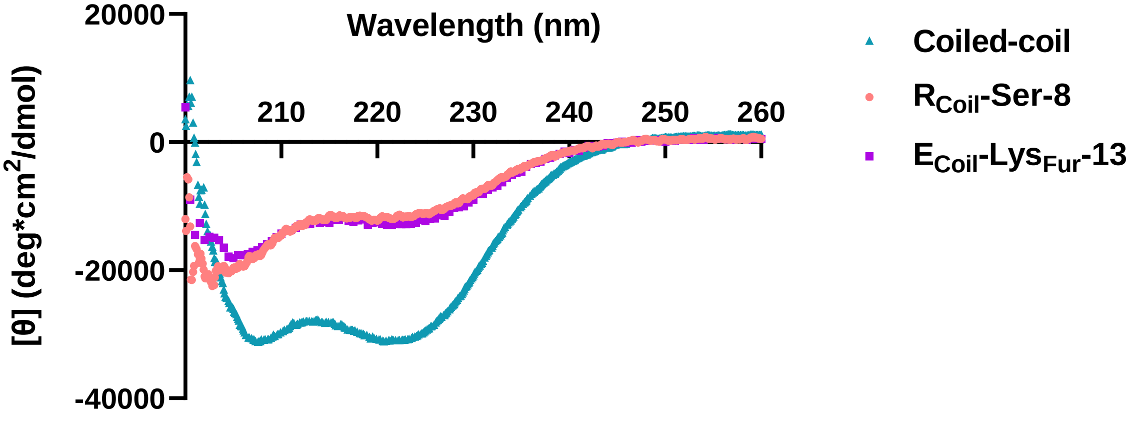

	R_{Coil}-Ser-8	E_{Coil}-Lys_{Fur}-13	Coiled-coil
$[\theta]_{222}$	-11680,46	-12733,78	-30886,28
$[\theta]_{208}$	-17299,43	-16355,18	-30972,72
**$[\theta]_{222}/[\theta]_{208}$ ratio**	**0,68**	**0,78**	**0,997**

Supplementary Figure 27. CD spectra obtained of the R_{Coil}-Ser-8 (light red), E_{Coil}-Lys_{Fur}-13 (purple), and the E/R coiled-coil equimolar mixture of both (green) in PBS at 5 µM.

6.2 CD spectroscopy of R_{Coil}-Cys-8 and E_{Coil}-Lys_{Fur}-13

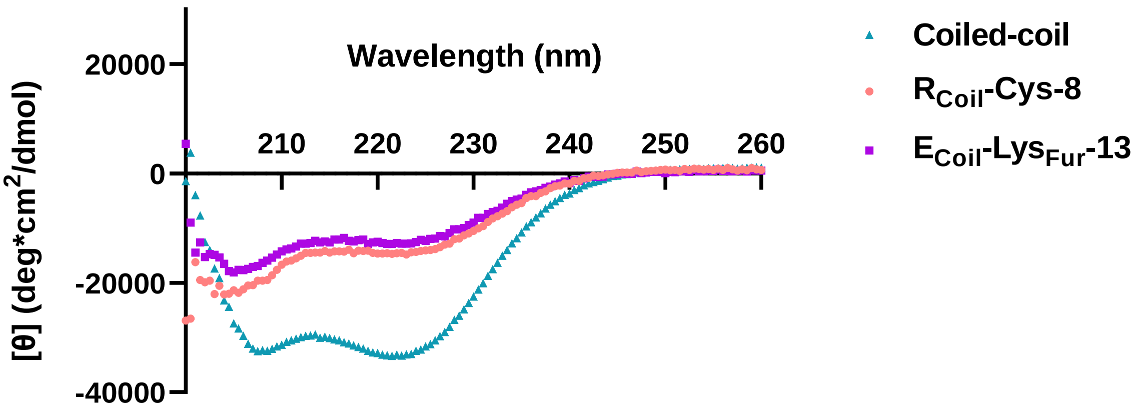

	R_{Coil}-Cys-8	E_{Coil}-Lys_{Fur}-13	Coiled-coil
$[\theta]_{222}$	-14571,59	-12733,78	-33136,65
$[\theta]_{208}$	-19621,08	-16355,18	-32355,26
**$[\theta]_{222}/[\theta]_{208}$ ratio**	**0,743**	**0,78**	**1,024**

Supplementtray Figure 28. CD spectra obtained of the R_{Coil}-Cys-8 (light red), E_{Coil}-Lys_{Fur}-13 (purple), and the E/R coiled-coil equimolar mixture of both (green) in PBS at 5 µM.

6.3 CD spectroscopy of R$_{Coil}$-His-8 and E$_{Coil}$-Lys$_{Fur}$-13

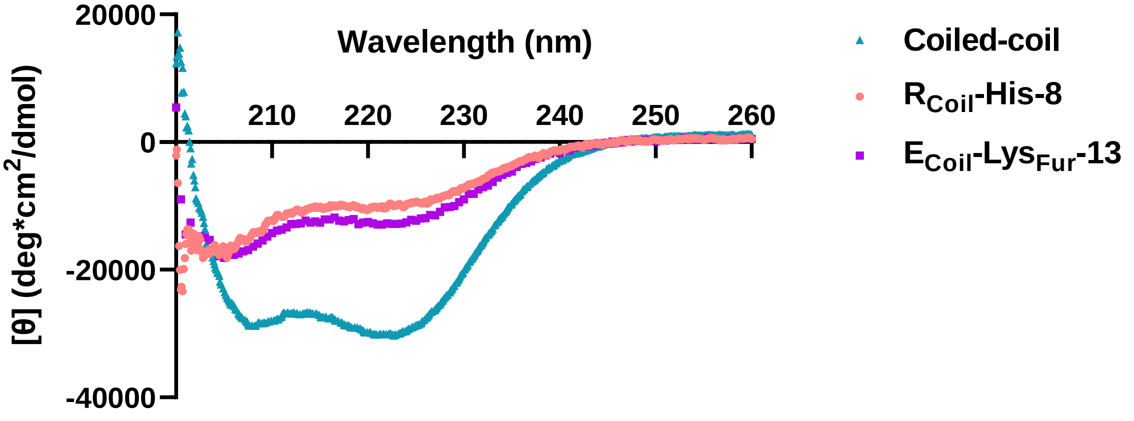

	R$_{Coil}$-His-8	E$_{Coil}$-Lys$_{Fur}$-13	Coiled-coil
**[θ]$_{222}$**	-10085,79	-12733,78	-30033,95
**[θ]$_{208}$**	-14173,71	-16355,18	-28663,20
**[θ]$_{222}$/[θ]$_{208}$ ratio**	**0,71**	**0,78**	**1,05**

Supplementary Figure 29. CD spectra obtained of the R$_{Coil}$-His-8 (light red), E$_{Coil}$-Lys$_{Fur}$-13 (purple), and the E/R coiled-coil equimolar mixture of both (green) in PBS at 5 µM.

6.4 CD spectroscopy of R_{Coil}-Tyr-8 and E_{Coil}-Lys_{Fur}-13

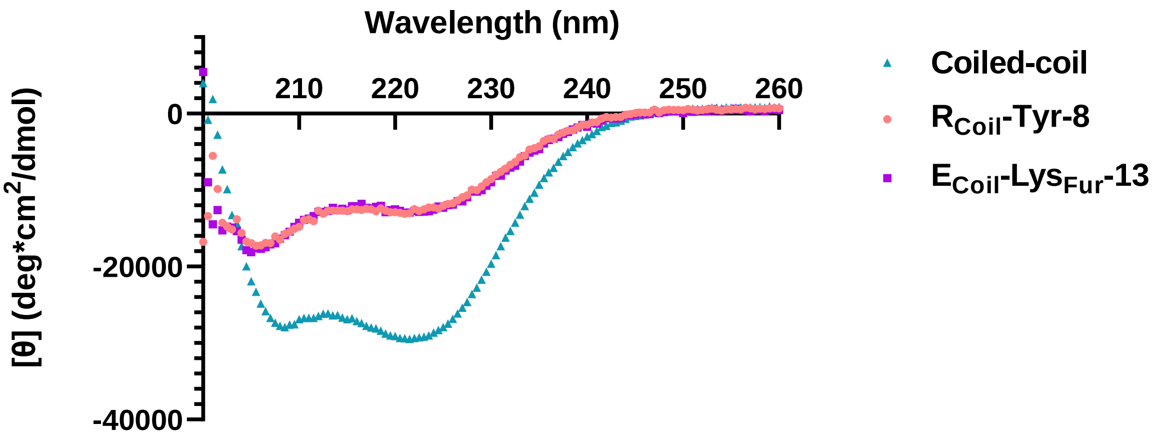

	R_{Coil}-Tyr-8	E_{Coil}-Lys_{Fur}-13	Coiled-coil
**$[\theta]_{222}$**	-12485,11	-12733,78	-29374,79
**$[\theta]_{208}$**	-16471,08	-16355,18	-27784,21
**$[\theta]_{222}/[\theta]_{208}$ ratio**	**0,76**	**0,78**	**1,06**

Supplementary Figure 30. CD spectra obtained of the R_{Coil}-Tyr-8 (light red), E_{Coil}-Lys_{Fur}-13 (purple), and the E/R coiled-coil equimolar mixture of both (green) in PBS at 5 µM.

6.5 CD spectroscopy of R_{Coil}-Trp-8 and E_{Coil}-Lys_{Fur}-13

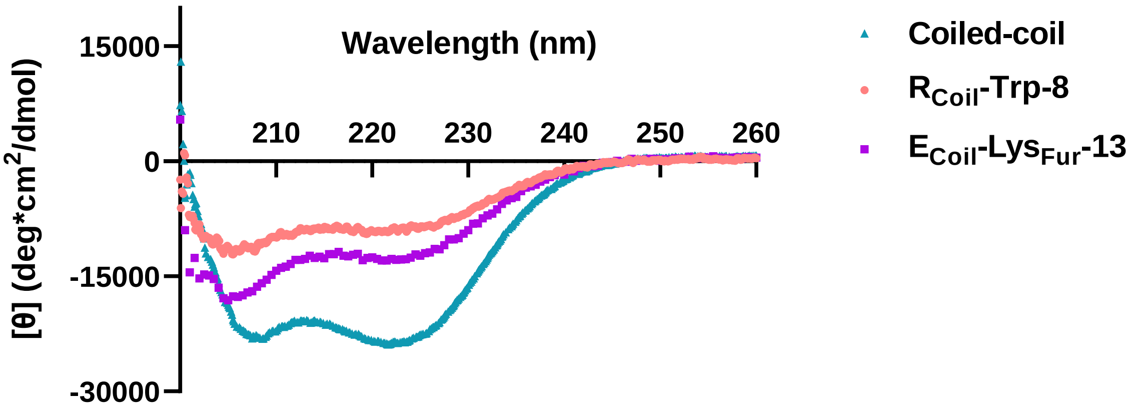

	R_{Coil}-Trp-8	E_{Coil}-Lys_{Fur}-13	Coiled-coil
$[\theta]_{222}$	-8846,93	-12733,78	-23677,99
$[\theta]_{208}$	-11225,24	-16355,18	-22816,67
**$[\theta]_{222}/[\theta]_{208}$ ratio**	**0,79**	**0,78**	**1,038**

Supplementary Figure 31. CD spectra obtained of the R_{Coil}-Trp-8 (light red), E_{Coil}-Lys_{Fur}-13 (purple), and the E/R coiled-coil equimolar mixture of both (green) in PBS at 5 µM.

7 RCoil scans with ECoil-OrnFur-13 peptide

7.1 Rhodamine B as a photosensitizer

7.1.1 RCoil-Ser-8 scan with ECoil-OrnFur-13

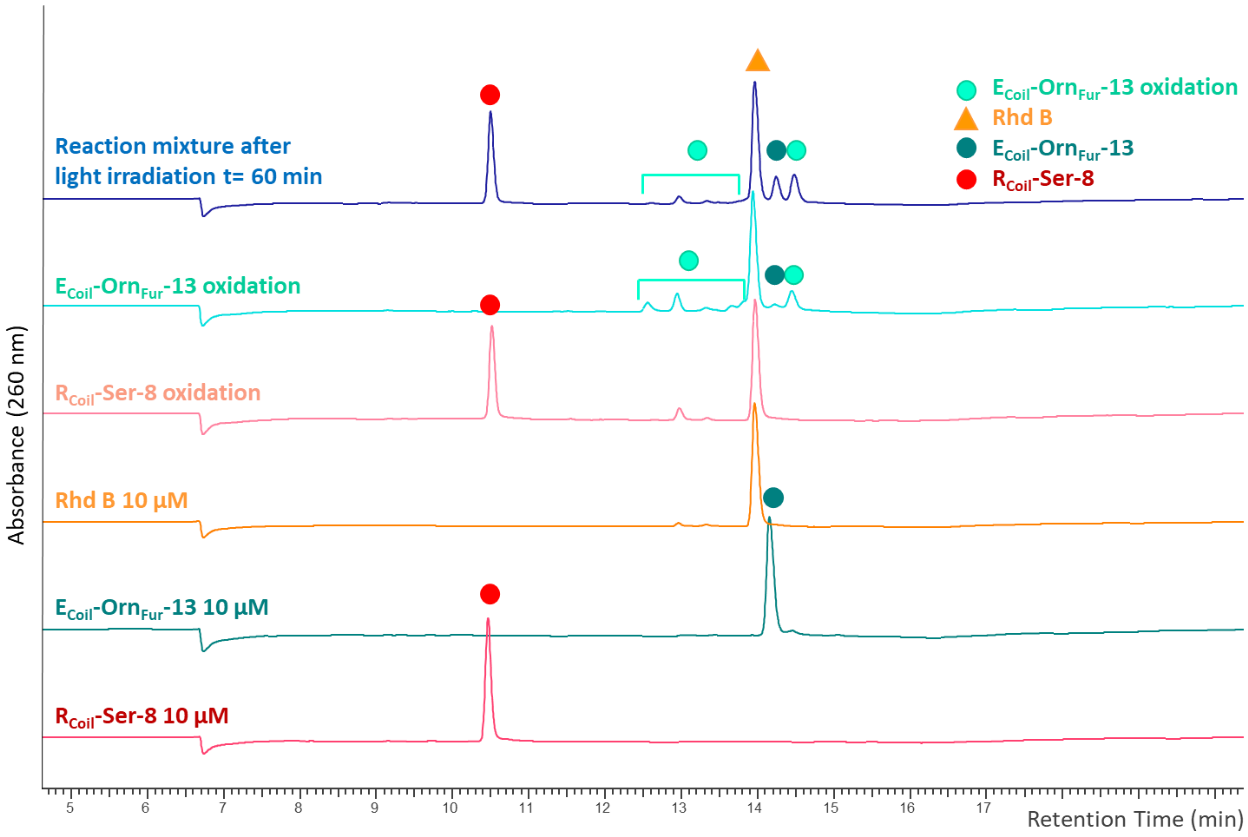

Supplementary Figure 32. HPLC-UV chromatograms recorded at 260 nm with a XTerra® Shield RP18 column, 125Å (5µM 2,1 x 250mm). The reaction mixture after light irradiation (blue trace) is the cross-link reaction between RCoil-Ser-8 (red trace) and ECoil-OrnFur-13 (dark green trace) after 60 minutes of light irradiation with Rhd B (orange trace) at 10 µM. The RCoil-Ser-8 oxidation (light red) trace and ECoil-OrnFur-13 oxidation (green) trace were generated by exposure to singlet oxygen by light irradiation in the presence of Rhd B at 10 µM for 60 minutes in absence of the other Coil.

7.1.2 R_{Coil}-Cys-8 scan with E_{Coil}-Orn$_{Fur}$-13

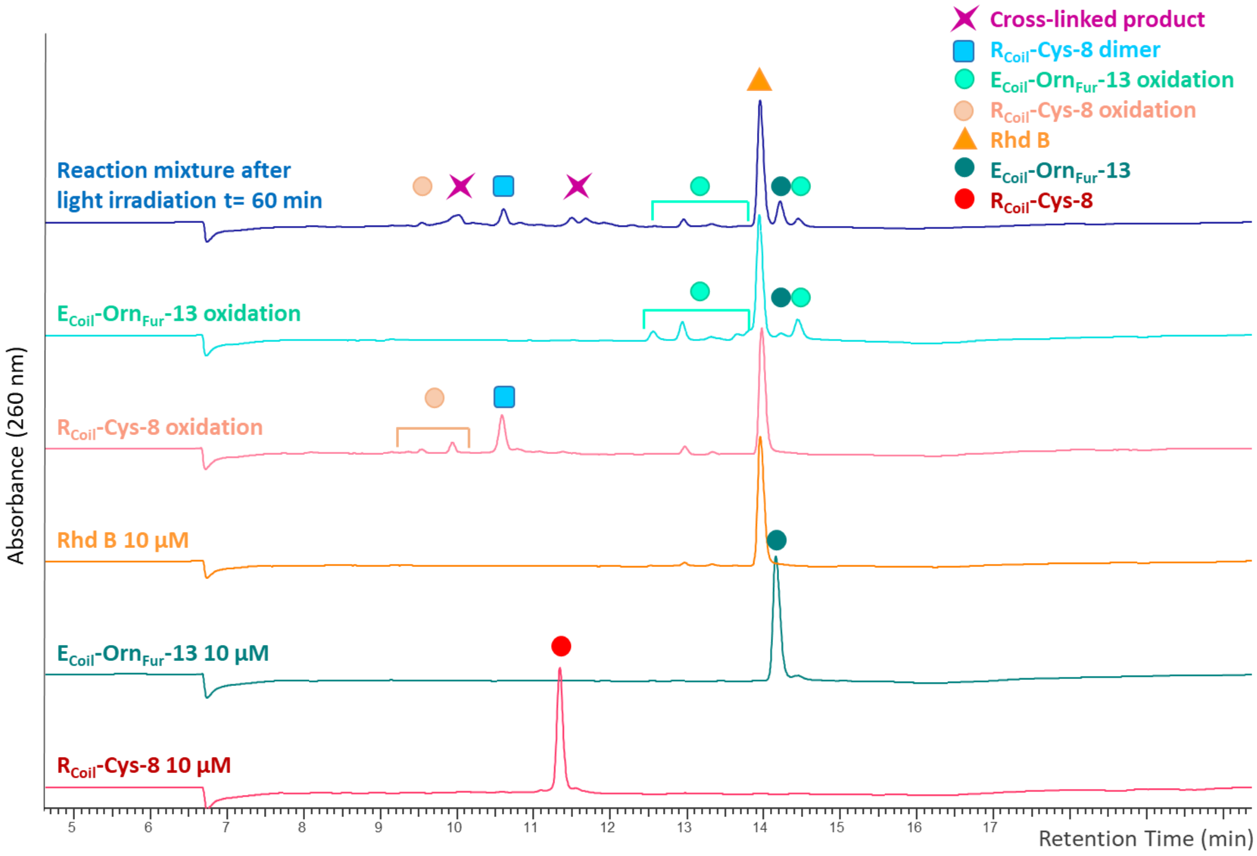

Supplementary Figure 33. HPLC-UV chromatograms recorded at 260 nm with a XTerra® Shield RP18 column, 125Å (5µM 2,1 x 250mm). The reaction mixture after light irradiation (blue trace) is the cross-link reaction between R_{Coil}-Cys-8 (red trace) and E_{Coil}-Orn$_{Fur}$-13 (dark green trace) after 60 minutes of light irradiation with Rhd B (orange trace) at 10 µM. The R_{Coil}-Cys-8 oxidation (light red) trace and E_{Coil}-Orn$_{Fur}$-13 oxidation (green) trace were generated by exposure to singlet oxygen by light irradiation in the presence of Rhd B at 10 µM for 60 minutes in absence of the other Coil.

7.1.3 R_{Coil}-His-8 scan with E_{Coil}-Orn_{Fur}-13

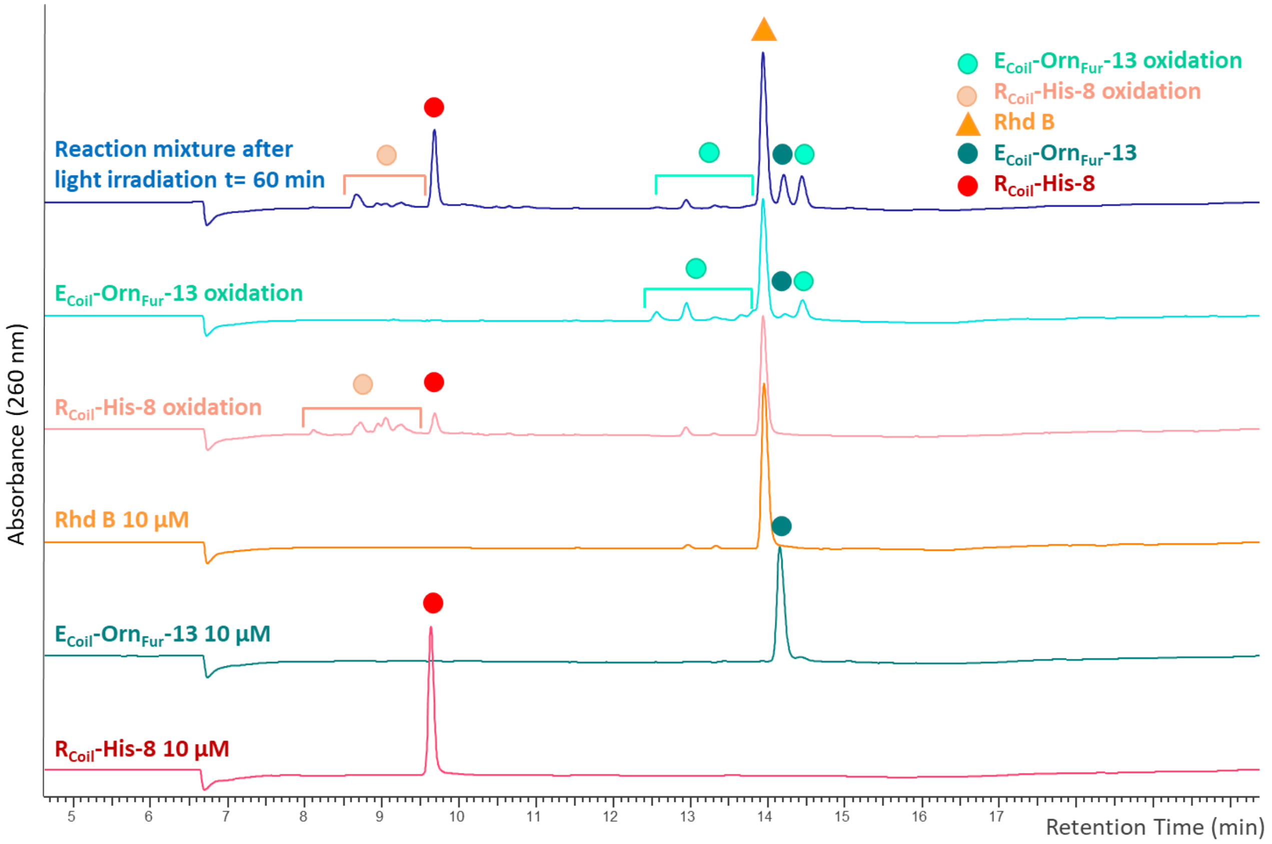

Supplementary Figure 34. HPLC-UV chromatograms recorded at 260 nm with a XTerra® Shield RP18 column, 125Å (5µM 2,1 x 250mm). The reaction mixture after light irradiation (blue trace) is the cross-link reaction between R_{Coil}-His-8 (red trace) and E_{Coil}-Orn_{Fur}-13 (dark green trace) after 60 minutes of light irradiation with Rhd B (orange trace) at 10 µM. The R_{Coil}-His-8 oxidation (light red) trace and E_{Coil}-Orn_{Fur}-13 oxidation (green) trace were generated by exposure to singlet oxygen by light irradiation in the presence of Rhd B at 10 µM for 60 minutes in absence of the other Coil

7.1.4 R_{Coil}-Tyr-8 scan with E_{Coil}-Orn_{Fur}-13

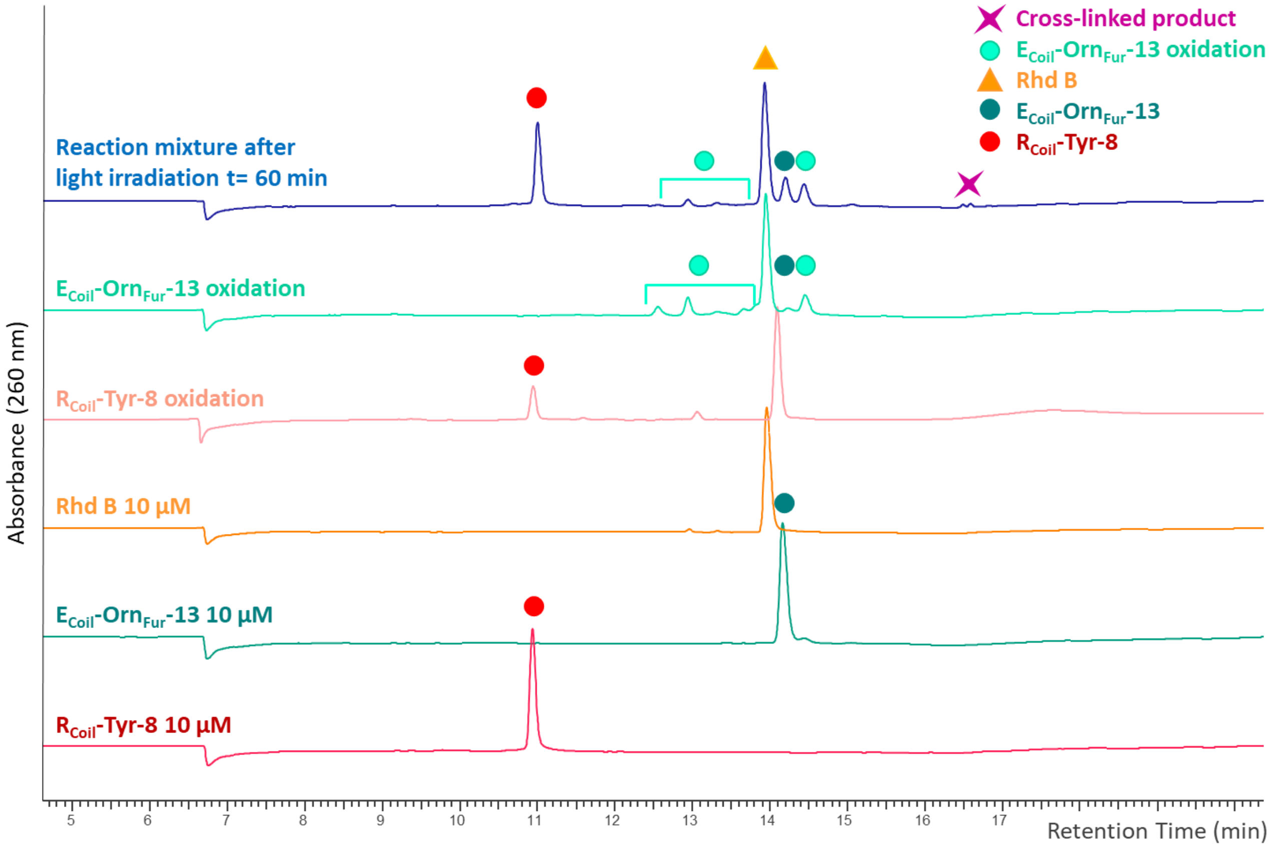

Supplementary Figure 35. HPLC-UV chromatograms recorded at 260 nm with a XTerra® Shield RP18 column, 125Å (5µM 2,1 x 250mm). The reaction mixture after light irradiation (blue trace) is the cross-link reaction between R_{Coil}-Tyr-8 (red trace) and E_{Coil}-Orn_{Fur}-13 (dark green trace) after 60 minutes of light irradiation with Rhd B (orange trace) at 10 µM. The R_{Coil}-Tyr-8 oxidation (light red) trace and E_{Coil}-Orn_{Fur}-13 oxidation (green) trace were generated by exposure to singlet oxygen by light irradiation in the presence of Rhd B at 10 µM for 60 minutes in absence of the other Coil.

7.1.5 R_{Coil}-Trp-8 scan with E_{Coil}-Orn_{Fur}-13

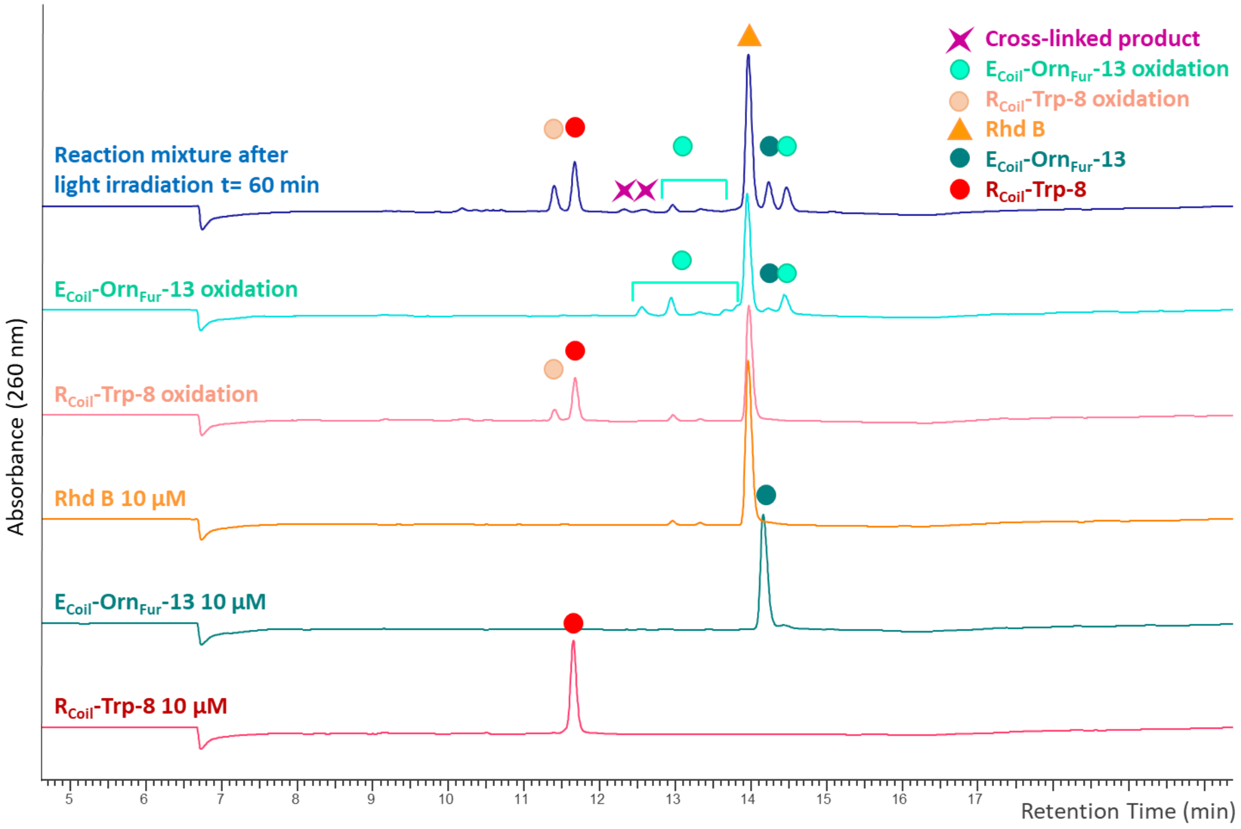

Supplementary Figure 36. HPLC-UV chromatograms recorded at 260 nm with a XTerra® Shield RP18 column, 125Å (5µM 2,1 x 250mm). The reaction mixture after light irradiation (blue trace) is the cross-link reaction between R_{Coil}-Trp-8 (red trace) and E_{Coil}-Orn_{Fur}-13 (dark green trace) after 60 minutes of light irradiation with Rhd B (orange trace) at 10 µM. The R_{Coil}-Trp-8 oxidation (light red) trace and E_{Coil}-Orn_{Fur}-13 oxidation (green) trace were generated by exposure to singlet oxygen by light irradiation in the presence of Rhd B at 10 µM for 60 minutes in absence of the other Coil.

7.2 Rose Bengal as a photosensitizer

7.2.1 R_{Coil}-Ser-8 scan with E_{Coil}-Orn_{Fur}-13

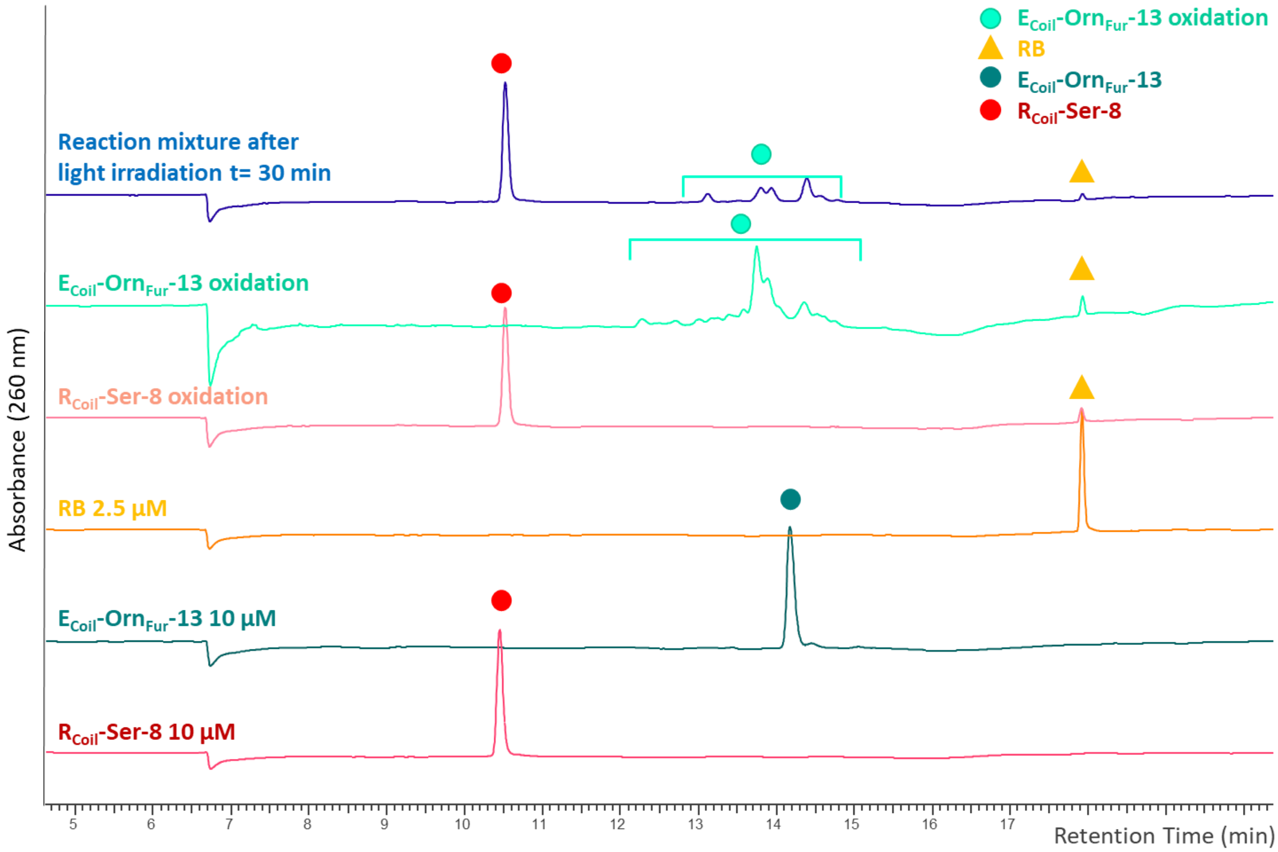

Supplementary Figure 37. HPLC-UV chromatograms recorded at 260 nm with a XTerra® Shield RP18 column, 125Å (5µM 2,1 x 250mm). The reaction mixture after light irradiation (blue trace) is the cross-link reaction between R_{Coil}-Ser-8 (red trace) and E_{Coil}-Orn_{Fur}-13 (dark green trace) after 30 minutes of light irradiation with RB (orange trace) at 2.5 µM. The R_{Coil}-Ser-8 oxidation (light red) trace and E_{Coil}-Orn_{Fur}-13 oxidation (green) trace were generated by exposure to singlet oxygen by light irradiation in the presence of RB at 2.5 µM for 30 minutes in absence of the other Coil.

7.2.2 R_{Coil}-Cys-8 scan with E_{Coil}-Orn_{Fur}-13

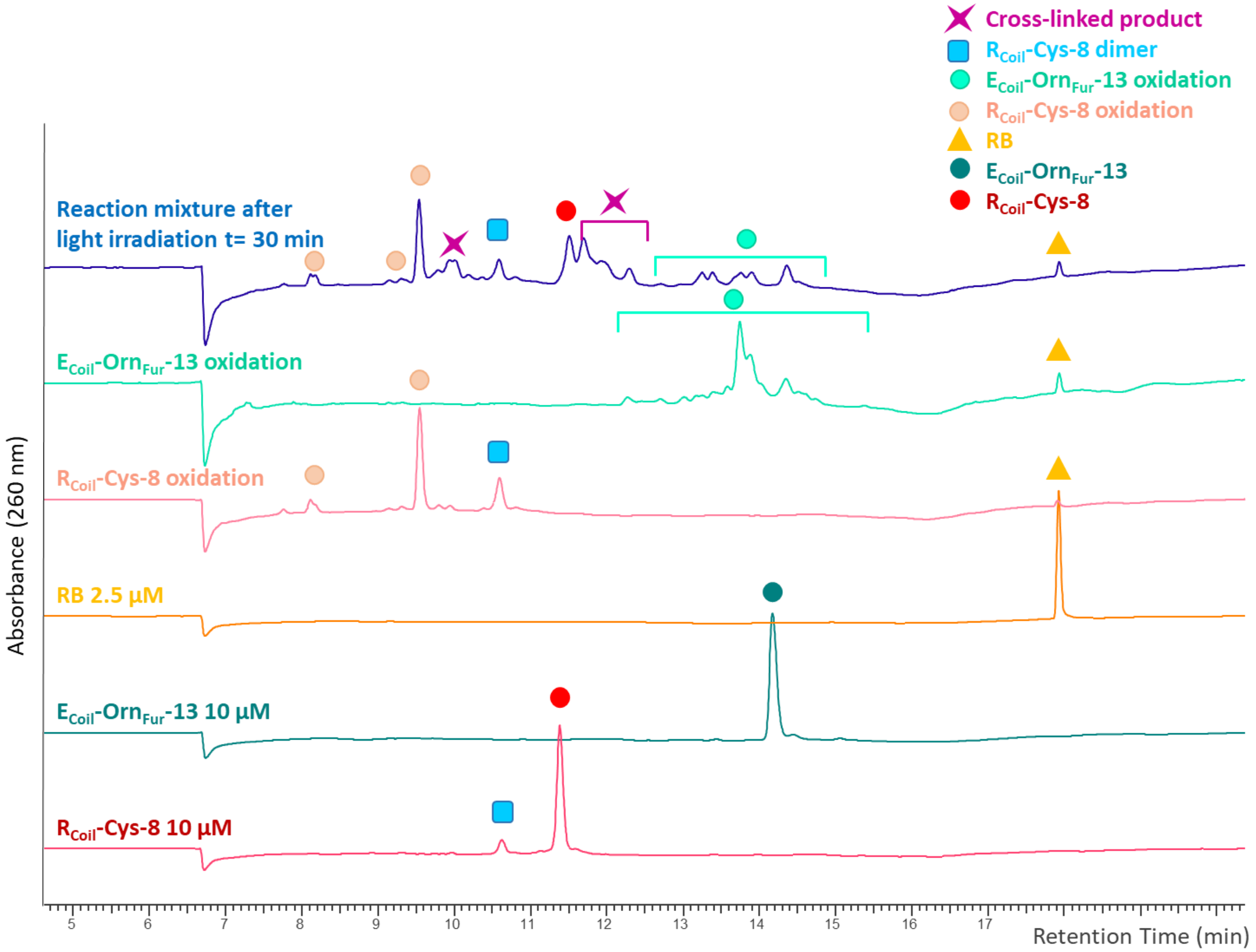

Supplementary Figure 38. HPLC-UV chromatograms recorded at 260 nm with a XTerra® Shield RP18 column, 125Å (5µM 2,1 x 250mm). The reaction mixture after light irradiation (blue trace) is the cross-link reaction between R_{Coil}-Cys-8 (red trace) and E_{Coil}-Orn_{Fur}-13 (dark green trace) after 30 minutes of light irradiation with RB (orange trace) at 2.5 µM. The R_{Coil}-Cys-8 oxidation (light red) trace and E_{Coil}-Orn_{Fur}-13 oxidation (green) trace were generated by exposure to singlet oxygen by light irradiation in the presence of RB at 2.5 µM for 30 minutes in absence of the other Coil.

7.2.3 R$_{Coil}$-His-8 scan with E$_{Coil}$-Orn$_{Fur}$-13

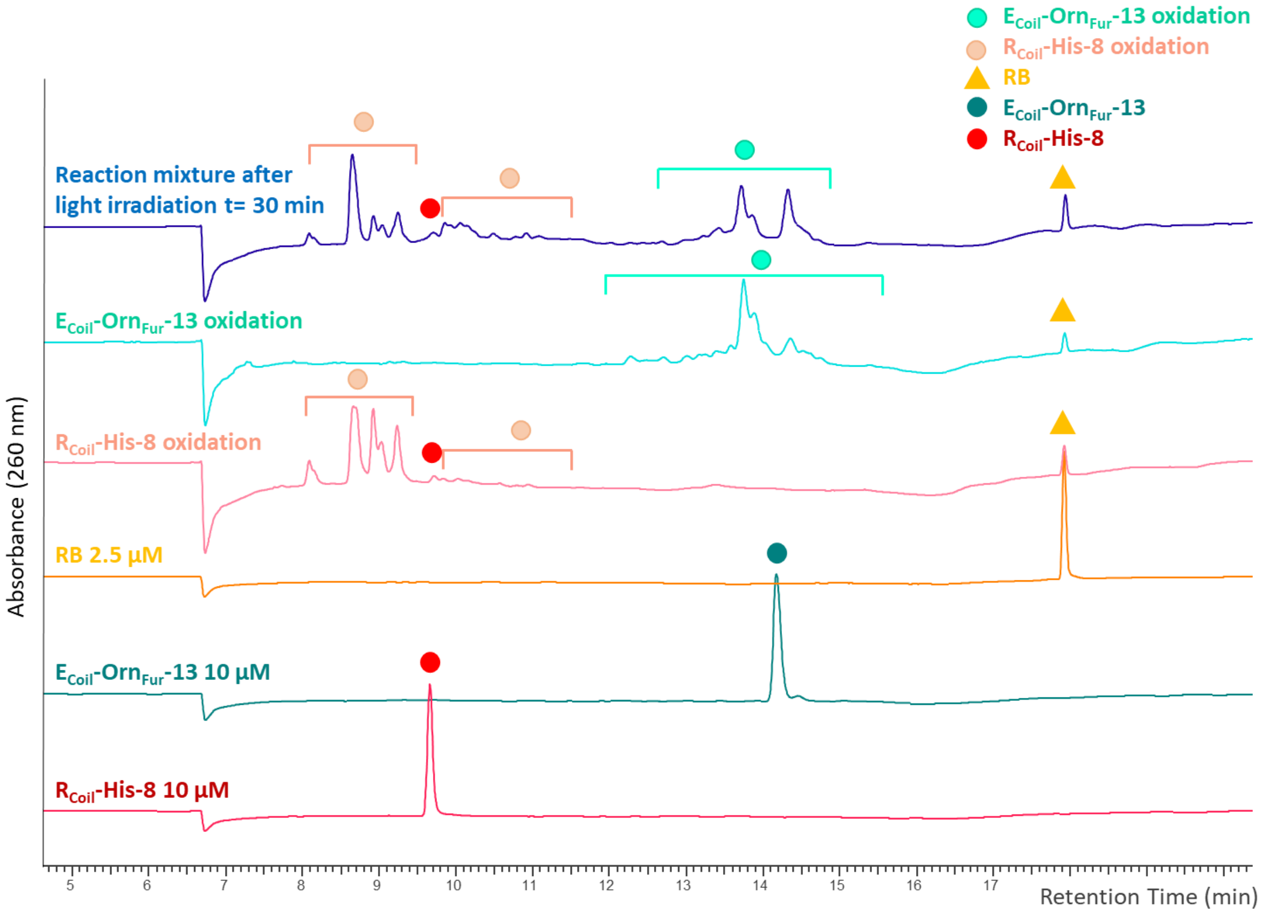

Supplementary Figure 39. HPLC-UV chromatograms recorded at 260 nm with a XTerra® Shield RP18 column, 125Å (5µM 2,1 x 250mm). The reaction mixture after light irradiation (blue trace) is the cross-link reaction between R$_{Coil}$-His-8 (red trace) and E$_{Coil}$-Orn$_{Fur}$-13 (dark green trace) after 30 minutes of light irradiation with RB (orange trace) at 2.5 µM. The R$_{Coil}$-His-8 oxidation (light red) trace and E$_{Coil}$-Orn$_{Fur}$-13 oxidation (green) trace were generated by exposure to singlet oxygen by light irradiation in the presence of RB at 2.5 µM for 30 minutes in absence of the other Coil.

7.2.4 R_{Coil}-Tyr-8 scan with E_{Coil}-Orn_{Fur}-13

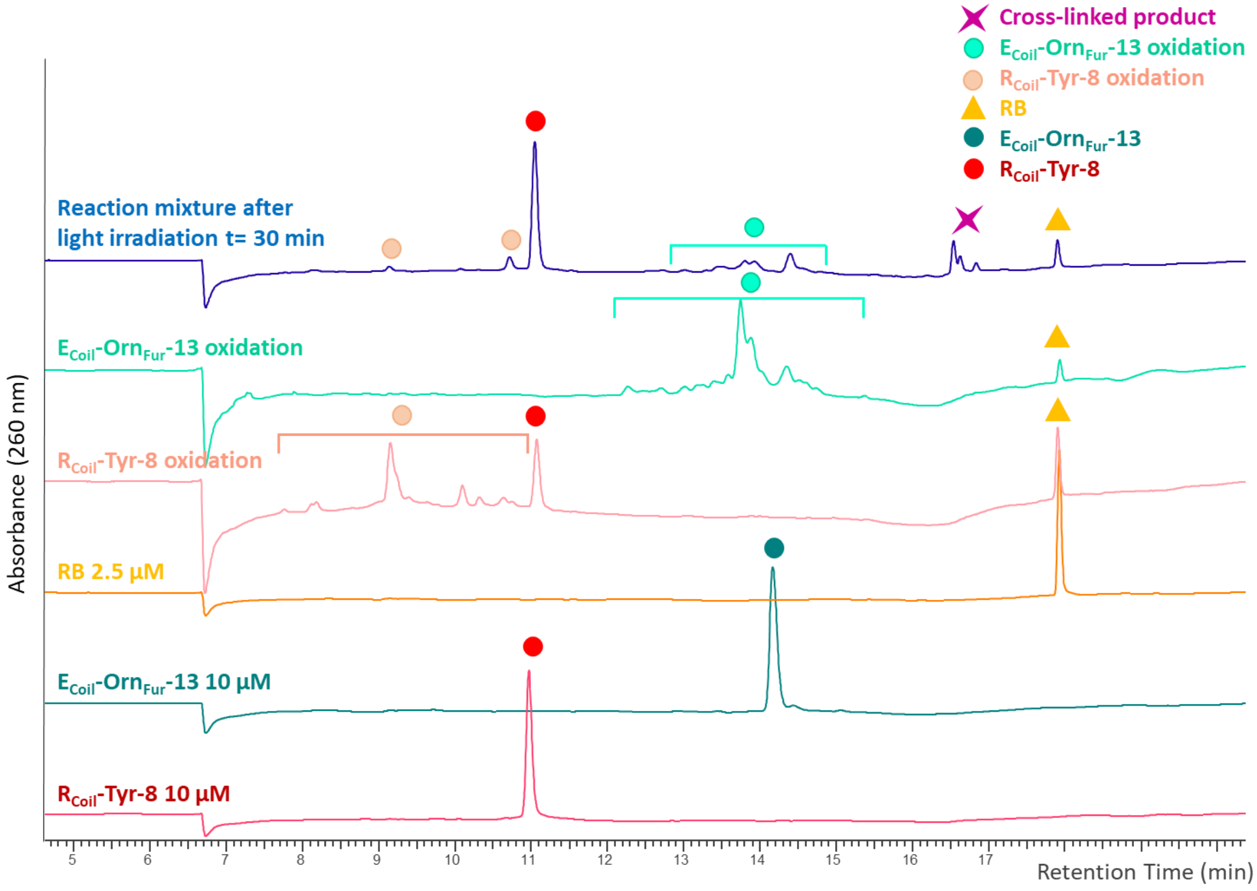

Supplementary Figure 40. HPLC-UV chromatograms recorded at 260 nm with a XTerra® Shield RP18 column, 125Å (5µM 2,1 x 250mm). The reaction mixture after light irradiation (blue trace) is the cross-link reaction between R_{Coil}-Tyr-8 (red trace) and E_{Coil}-Orn_{Fur}-13 (dark green trace) after 30 minutes of light irradiation with RB (orange trace) at 2.5 µM. The R_{Coil}-Tyr-8 oxidation (light red) trace and E_{Coil}-Orn_{Fur}-13 oxidation (green) trace were generated by exposure to singlet oxygen by light irradiation in the presence of RB at 2.5 µM for 30 minutes in absence of the other Coil.

7.2.5 R$_{Coil}$-Trp-8 scan with E$_{Coil}$-Orn$_{Fur}$-13

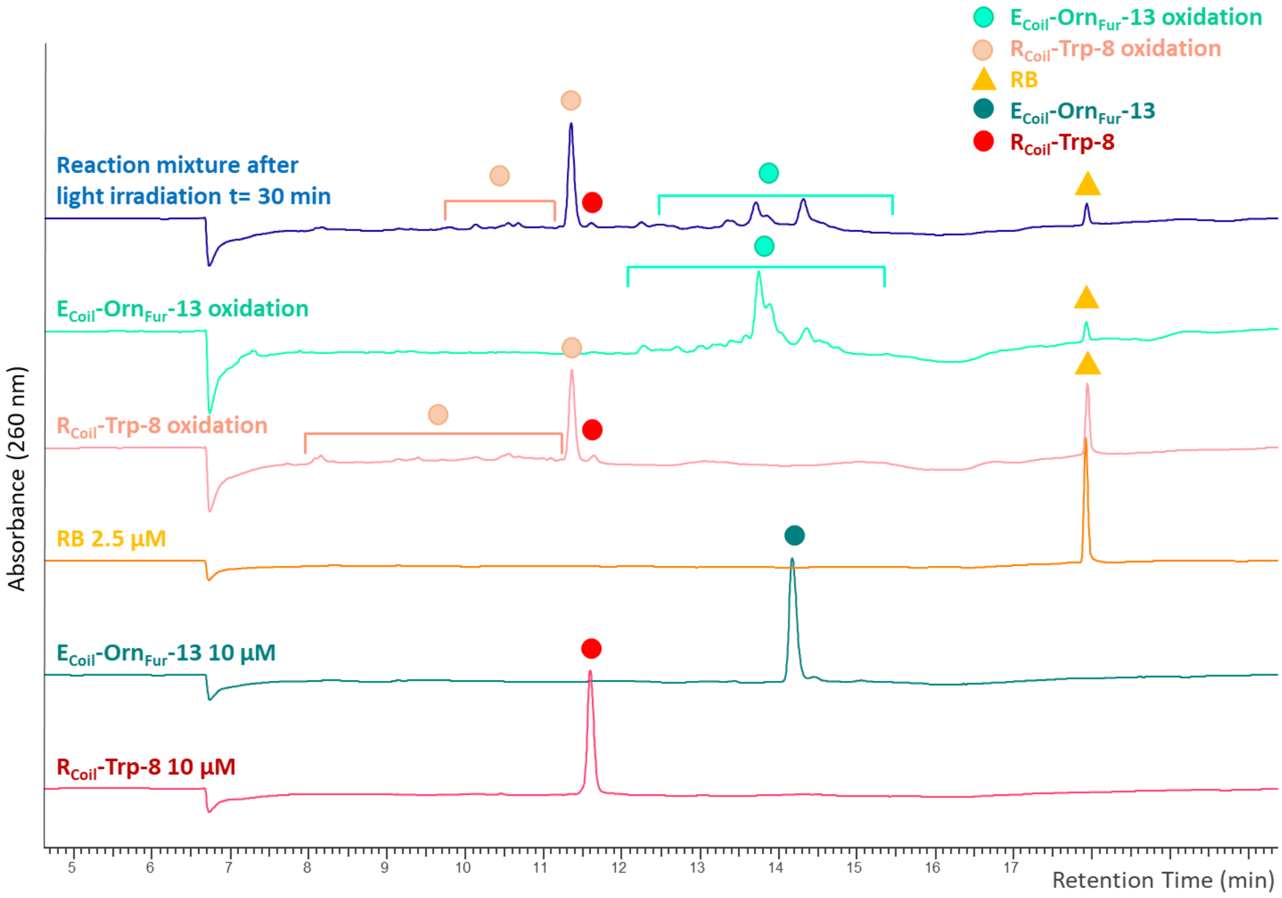

Supplementary Figure 41. HPLC-UV chromatograms recorded at 260 nm with a XTerra® Shield RP18 column, 125Å (5µM 2,1 x 250mm). The reaction mixture after light irradiation (blue trace) is the cross-link reaction between R$_{Coil}$-Trp-8 (red trace) and E$_{Coil}$-Orn$_{Fur}$-13 (dark green trace) after 30 minutes of light irradiation with RB (orange trace) at 2.5 µM. The R$_{Coil}$-Trp-8 oxidation (light red) trace and E$_{Coil}$-Orn$_{Fur}$-13 oxidation (green) trace were generated by exposure to singlet oxygen by light irradiation in the presence of RB at 2.5 µM for 30 minutes in absence of the other Coil.

8 Optimization of cross-linking conditions for Cys

8.1 Cross-link between R_{Coil}-Cys-8 and E_{Coil}-X_{Fur}-136

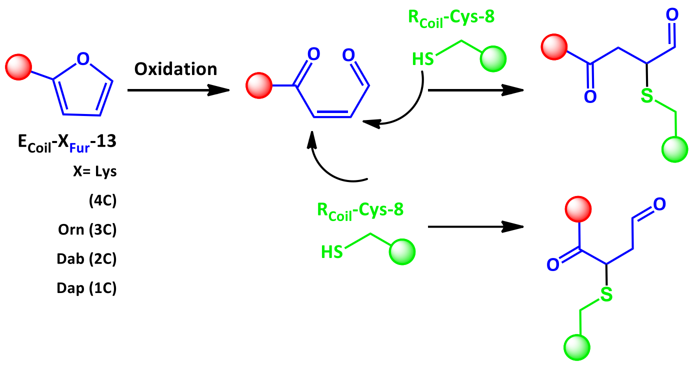

Supplementary Figure 42. Chemical structure of the plausible covalent bond that can be formed between R_{Coil}-Cys-8 and E_{Coil}-X_{Fur}-13. The cross-linked products are synthesized in Michael-type addition of the cysteine thiol group of R_{Coil}-Cys-8 to the activated furan moiety (keto-enal-E_{Coil}) after generation of singlet oxygen. The addition of the thiol may occur at the α or β-position relative to the ketone group. All synthesized cross-linked products are the mixtures of R and S enantiomers.

8.2 Quantitative analysis of the cross-link yield for Figure 7 in the manuscript

	Peak Area (Y units/ms)	% XL area
**Trace E**		
R_{Coil}-Cys-8	917619	
**Cross-linked product**	3986282	**81,3**
Total area	4903901	
**Trace D**		
R_{Coil}-Cys-8 + R_{Coil}-Cys-8 dimer	1525637	
**Cross-linked product**	4170034	**73,2**
Total area	5695671	
**Trace C**		
R_{Coil}-Cys-8 + R_{Coil}-Cys-8 dimer	1397282	
**Cross-linked product**	4569304	**72,9**
Total area	6269906	
**Trace B**		
R_{Coil}-Cys-8 + R_{Coil}-Cys-8 dimer	1280999	
**Cross-linked product**	2629132	**67,2**
Total area	3910131	
**Trace A**		
R_{Coil}-Cys-8 + R_{Coil}-Cys-8 dimer	779910	
**Cross-linked product**	1816636	**69,2**
Total area	2625953	

Table 4. The peak area was calculated integrating the peak in the HPLC chromatogram. The percentage of the cross-linked product (XL) area is the ratio between the XL area and the total area, multiplied by 100. The total area considered is the area of the R_{Coil}-Cys-8 peak plus the area of the R_{Coil}-Cys-8 dimer peak plus the area of the cross-linked product.

8.3 R_{Coil}-Cys-8 dimerization by irradiation with Rhodamine B

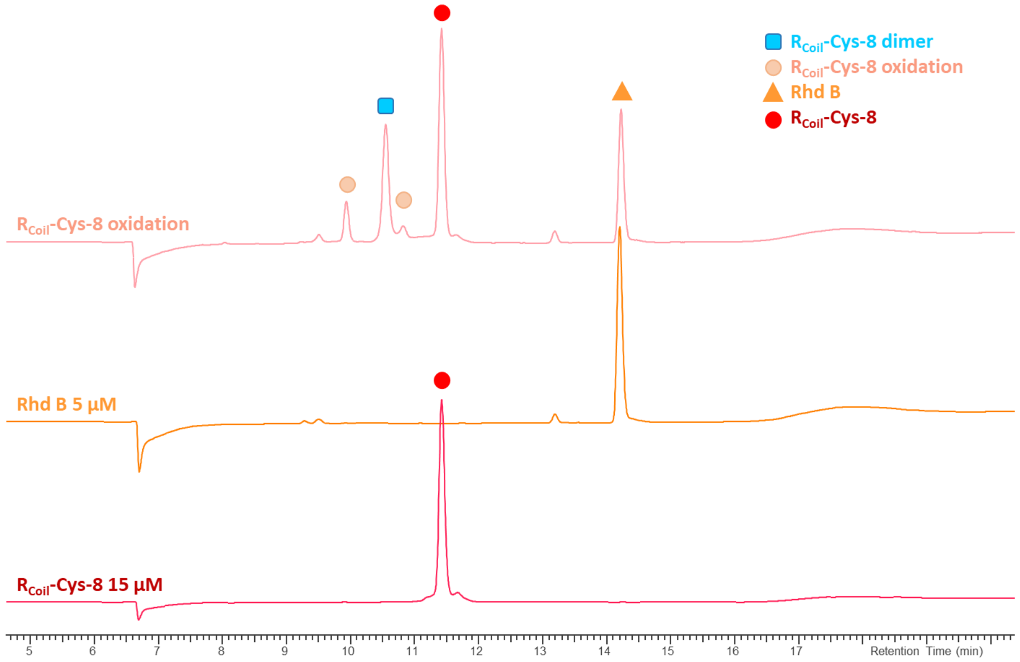

Supplementary Figure 43. HPLC-UV chromatograms recorded at 260 nm with a XTerra® Shield RP18 column, 125Å (5µM 2,1 x 250mm). The R_{Coil}-Cys-8 (red trace) is exposed to singlet oxygen generation by light irradiation in the presence of Rhd B (orange trace) at 5 µM for 60 minutes to give R_{Coil}-Cys-8 oxidation (light red trace).

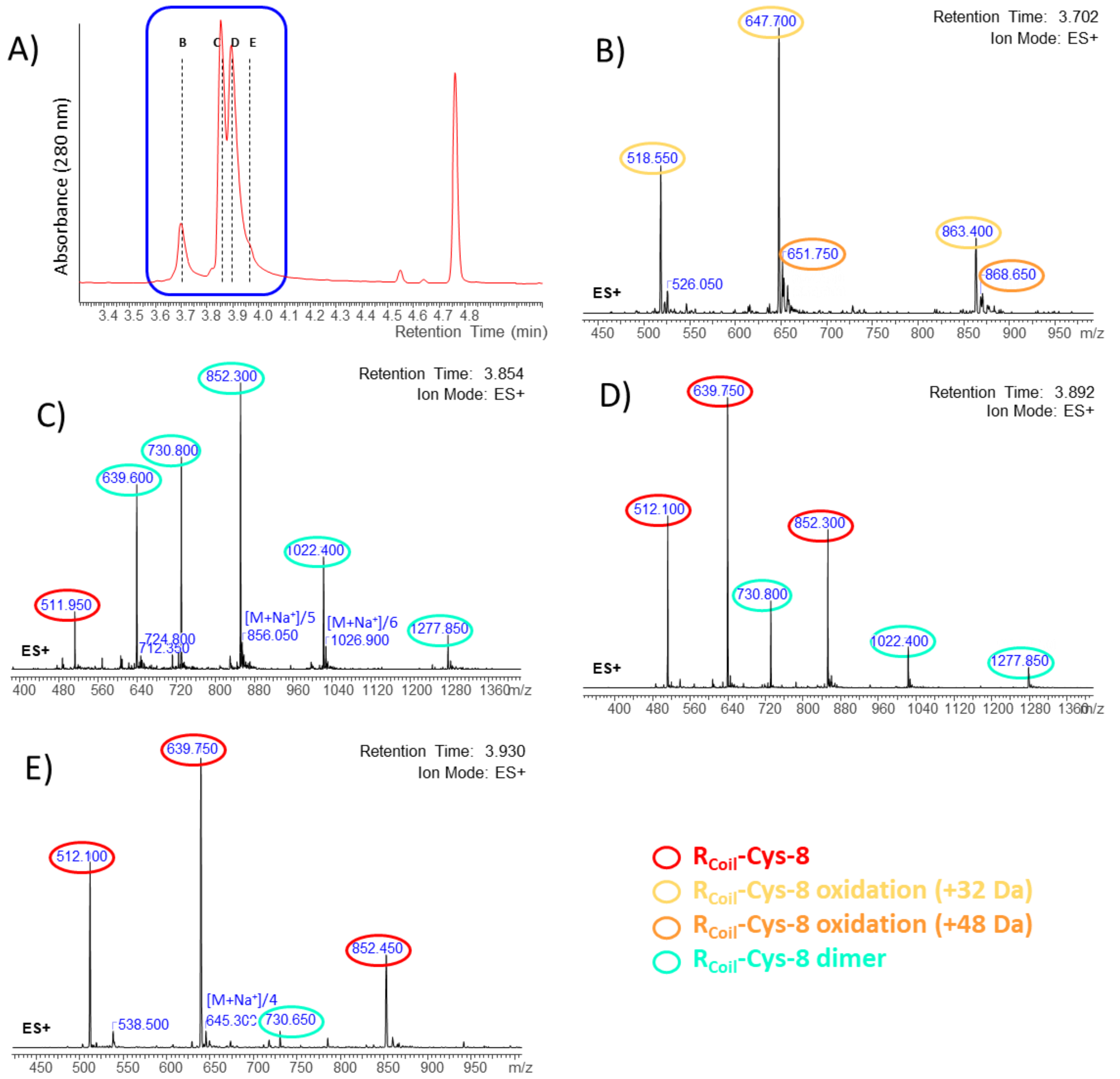

Supplementary Figure 44. A) HPLC-UV chromatogram recorded at 280 nm with the column Phenomenex Kinetex EVO C18 100 Å (150 x 4.6 mm, 5 µm, at 35 °C). B-E) ESI-MS spectrum of the peaks at 3.702, 3.854, 3.892, and 3.930 minutes in A (blue rectangle). The red circles correspond to the mass-to-charge ratio of ions of R_{Coil}-Cys-8. The yellow and orange circles correspond to the fragment ions of the oxidated R_{Coil}-Cys-8, +32 Da (2O) and +48 Da (3O) respectively. The blue circles correspond to the mass-to-charge ratio of ions of R_{Coil}-Cys-8 dimerization: $[M+4H]^{4+}/4$= 1277.8; $[M+5H]^{5+}/5$= 1022.4; $[M+6H]^{6+}/6$= 852.3; $[M+7H]^{7+}/7$= 730.8, $[M+8H]^{8+}/8$= 639.6. Exact mass (m/z) and molecular weight (MW) calculated for $C_{222}H_{382}N_{76}O_{58}S_2$ $[M+1H]^+$: 5104.87 and 5108.01 Da; found 5108.5 Da.

8.4 Cross-linked product of R_{Coil}-Cys-8 with E_{Coil}-Lys_{Fur}-13

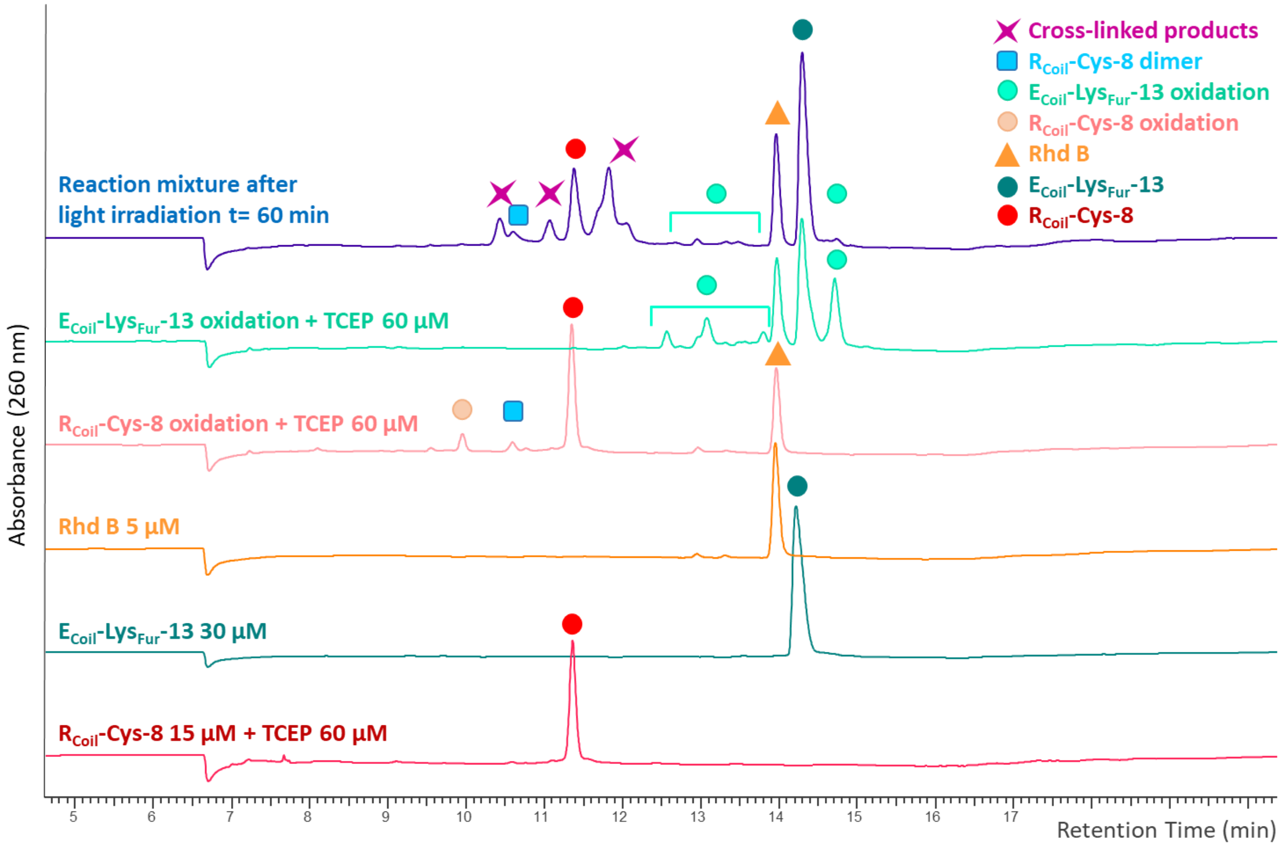

	Peak Area (Y units/ms)	*% XL product*
R_{Coil}-Cys-8 + R_{Coil}-Cys-8 dimer	3197502	
Cross-linked product	6401690	**66.7**
Total area	9599193	

Supplementary Figure 45. HPLC-UV chromatograms recorded at 260 nm with a XTerra® Shield RP18 column, 125Å (5µM 2,1 x 250mm). The reaction mixture after light irradiation (blue trace) is the cross-link reaction between R_{Coil}-Cys-8 + TCEP (red trace) and E_{Coil}-Lys_{Fur}-13 (dark green trace) after 60 minutes of light irradiation with Rhd B (orange trace) at 5 µM. The R_{Coil}-Cys-8 oxidation + TCEP (light red) trace and E_{Coil}-Lys_{Fur}-13 oxidation + TCEP (green) trace were generated by exposure to singlet oxygen by light irradiation in the presence of Rhd B at 5 µM for 60 minutes in absence of the other Coil. The cross-link yield was quantified as a percentage of the cross-linked product area and the values are indicated in the Table.

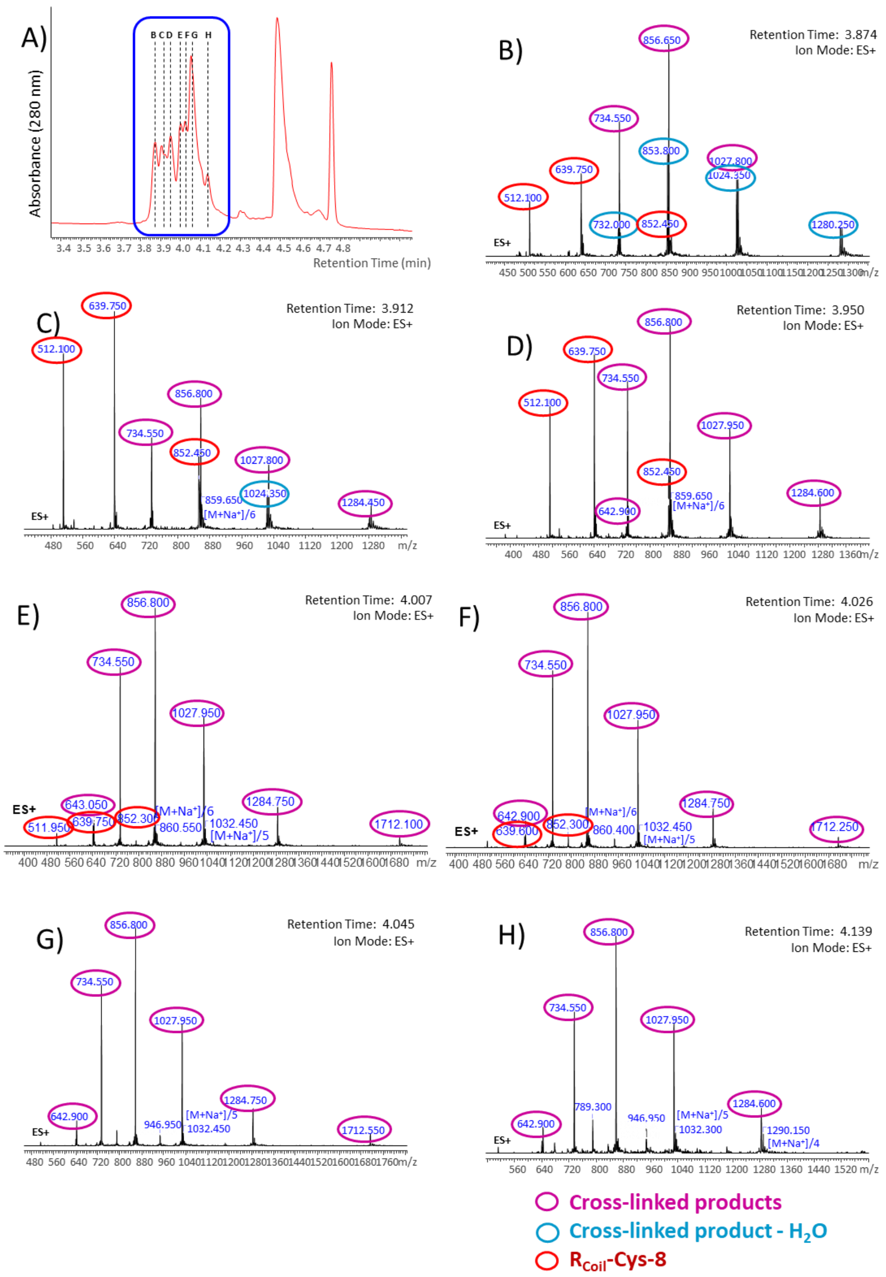

Supplementary Figure 46. A) HPLC-UV chromatogram recorded at 280 nm with the column Phenomenex Kinetex EVO C18 100 Å (150 x 4.6 mm, 5 µm, at 35 °C). B-H) ESI-MS spectrum of the peaks at 3.874, 3.912, 3.950, 4.007, 4.026, 4.045, and 4.139 minutes corresponding to the cross-linked products formed between R_{Coil}-Cys-8 and E_{Coil}-Lys_{Fur}-13 (blue rectangle in A). The red circles correspond to the mass-to-charge ratio of ions of R_{Coil}-Cys-8. The purple circles correspond to the mass-to-charge ratio of ions of the cross-linked product: $[M+3H]^{3+}/3$= 1712.2; $[M+4H]^{4+}/4$= 1284.7; $[M+5H]^{5+}/5$= 1027.9; $[M+6H]^{6+}/6$= 856.8; $[M+7H]^{7+}/7$= 734.5, $[M+7H]^{7+}/7$= 642.9. Exact mass (m/z) and molecular weight (MW) calculated for $C_{230}H_{387}N_{65}O_{65}S$ $[M+1H]^{+}$: 5131.9 Da and 5135.0; found 5135.0 Da. The blue circles correspond to the dehydrated (-18 Da) form of the cross-linked product. Peaks not assigned correspond to degradation products.

8.5 Cross-linked product of R_{Coil}-Cys-8 with E_{Coil}-Orn_{Fur}-13

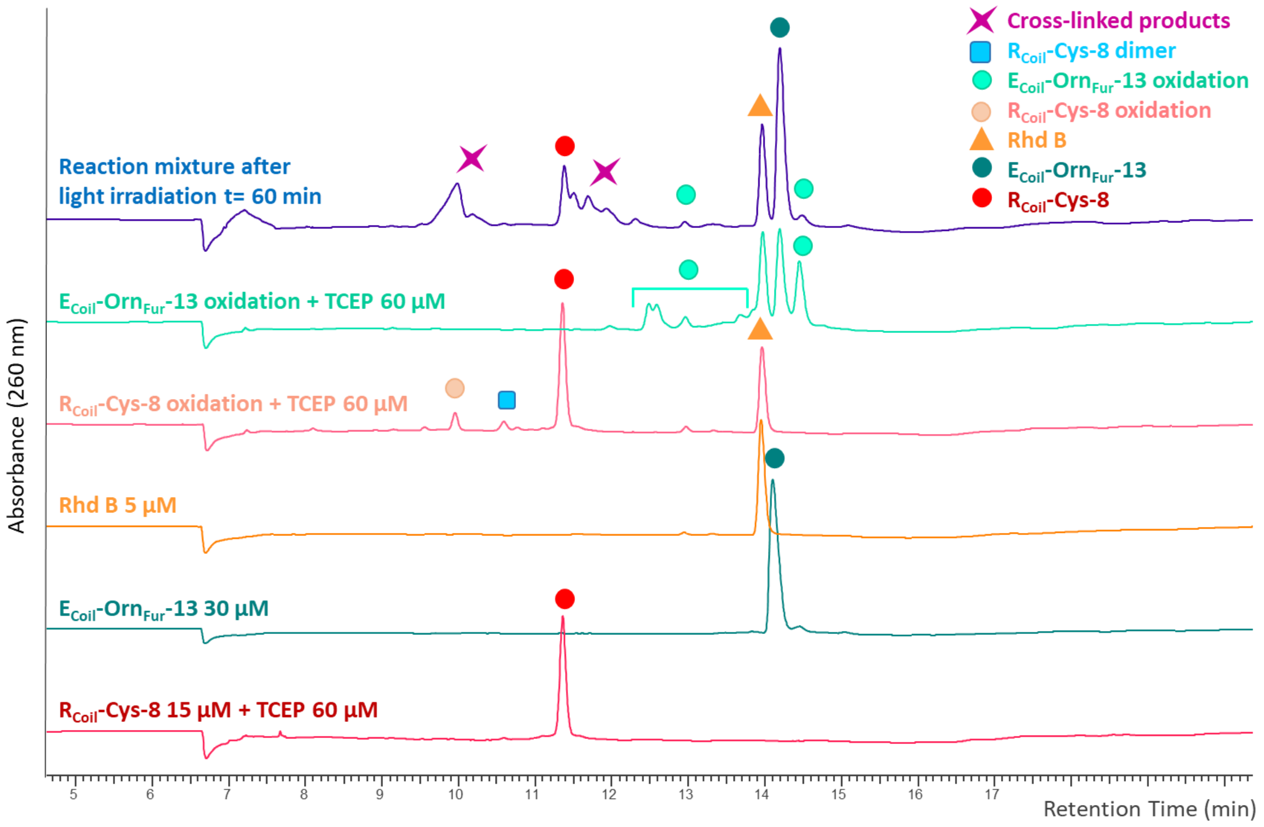

	Peak Area (Y units/ms)	*% XL area*
R_{Coil}-Cys-8	917619	
Cross-linked product	3986282	**81.3**
Total area	4903901	

Supplementary Figure 47. HPLC-UV chromatograms recorded at 260 nm with a XTerra® Shield RP18 column, 125Å (5µM 2,1 x 250mm). The reaction mixture after light irradiation (blue trace) is the cross-link reaction between R_{Coil}-Cys-8 + TCEP (red trace) and E_{Coil}-Orn_{Fur}-13 (dark green trace) after 60 minutes of light irradiation with Rhd B (orange trace) at 5 µM. The R_{Coil}-Cys-8 oxidation + TCEP (light red) trace and E_{Coil}-Orn_{Fur}-13 oxidation + TCEP (green) trace were generated by exposure to singlet oxygen by light irradiation in the presence of Rhd B at 5 µM for 60 minutes in absence of the other Coil. The cross-link yield was quantified as a percentage of the cross-linked product area and the values are indicated in the Table.

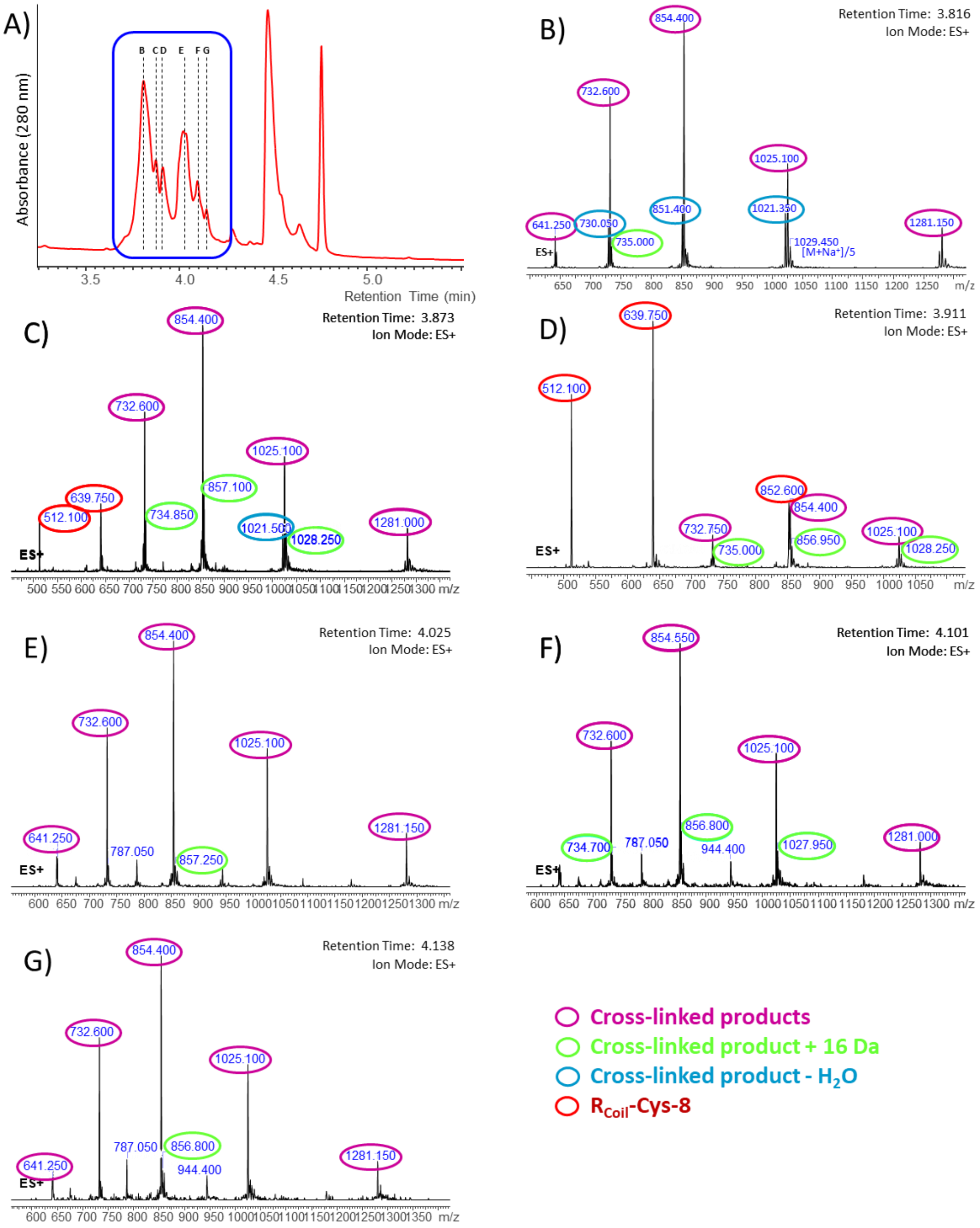

Supplementary Figure 48. A) HPLC-UV chromatogram recorded at 280 nm with the column Phenomenex Kinetex EVO C18 100 Å (150 x 4.6 mm, 5 µm, at 35 °C). B-H) ESI-MS spectrum of the peaks at 3.816, 3.873, 3.911, 4.025, 4.101, and 4.138 minutes corresponding to the cross-linked products formed between R_{Coil}-Cys-8 and E_{Coil}-Orn_{Fur}-13 (blue rectangle in A). The red circles correspond to the mass-to-charge ratio of ions of R_{Coil}-Cys-8. The purple circles correspond to the mass-to-charge ratio of ions of the cross-linked product: $[M+4H]^{4+}/4$= 1281.1; $[M+5H]^{5+}/5$= 1025.1; $[M+6H]^{6+}/6$= 854.4; $[M+7H]^{7+}/7$= 732.6, $[M+7H]^{7+}/7$= 641.2. Exact mass (m/z) and molecular weight (MW) calculated for $C_{229}H_{385}N_{65}O_{65}S$ $[M+1H]^{+}$: 5117.9 Da and 5121.0; found 5120.9 Da. The blue circles correspond to the dehydrated (-18 Da) form of the cross-linked product, and the green circles correspond to the oxidated cross-linked product (+16 Da). Peaks not assigned correspond to degradation products.

8.6 Cross-linked product of R_{Coil}-Cys-8 with E_{Coil}-Dab_{Fur}-13

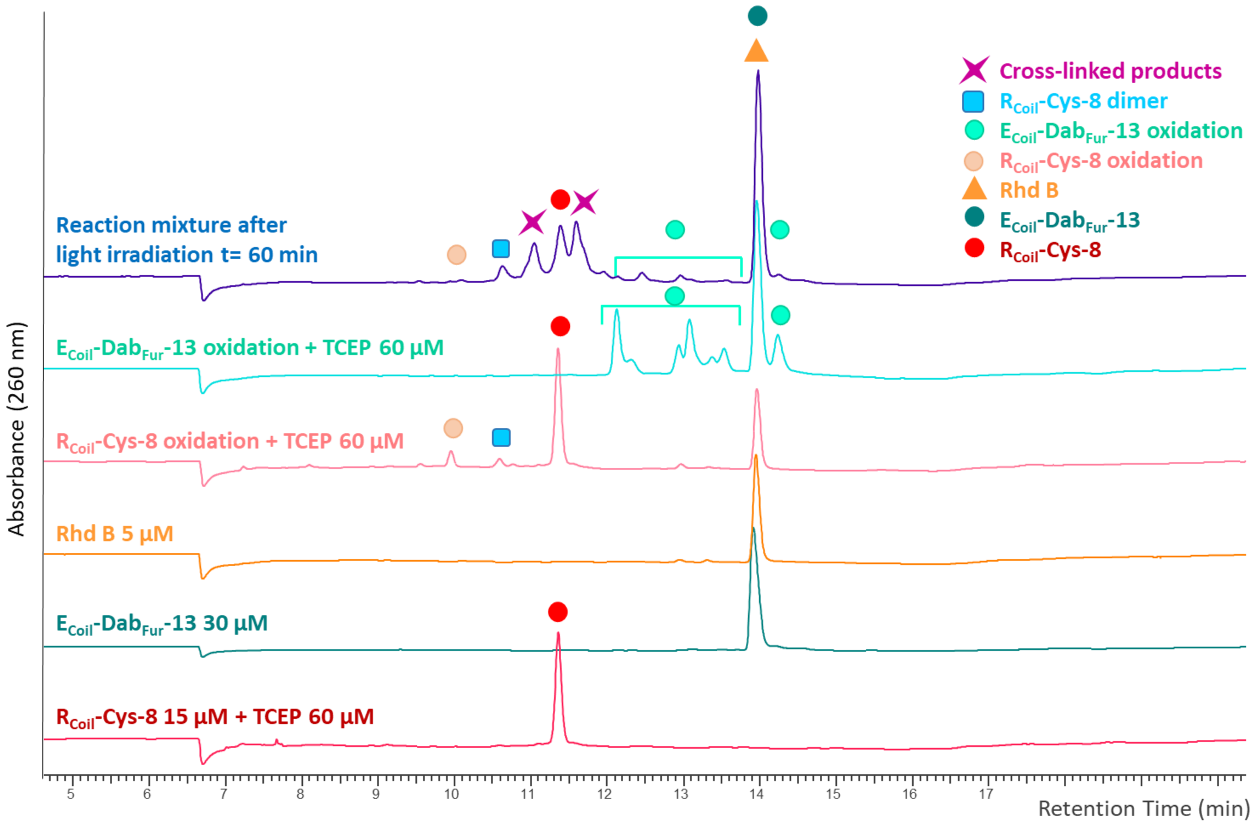

	Peak Area (Y units/ms)	*% XL area*
R_{Coil}-Cys-8 + R_{Coil}-Cys-8 dimer	2035419	
Cross-linked product	3467359	**61.5**
Total area	5502778	

Supplementary Figure 49. HPLC-UV chromatograms recorded at 260 nm with a XTerra® Shield RP18 column, 125Å (5µM 2,1 x 250mm). The reaction mixture after light irradiation (blue trace) is the cross-link reaction between R_{Coil}-Cys-8 + TCEP (red trace) and E_{Coil}-Dab_{Fur}-13 (dark green trace) after 60 minutes of light irradiation with Rhd B (orange trace) at 5 µM. The R_{Coil}-Cys-8 oxidation + TCEP (light red) trace and E_{Coil}-Dab_{Fur}-13 oxidation + TCEP (green) trace were generated by exposure to singlet oxygen by irradiation in the presence of Rhd B at 5 µM for 60 minutes in absence of the other Coil. The cross-link yield was quantified as a percentage of the cross-linked product area and the values are indicated in the Table.

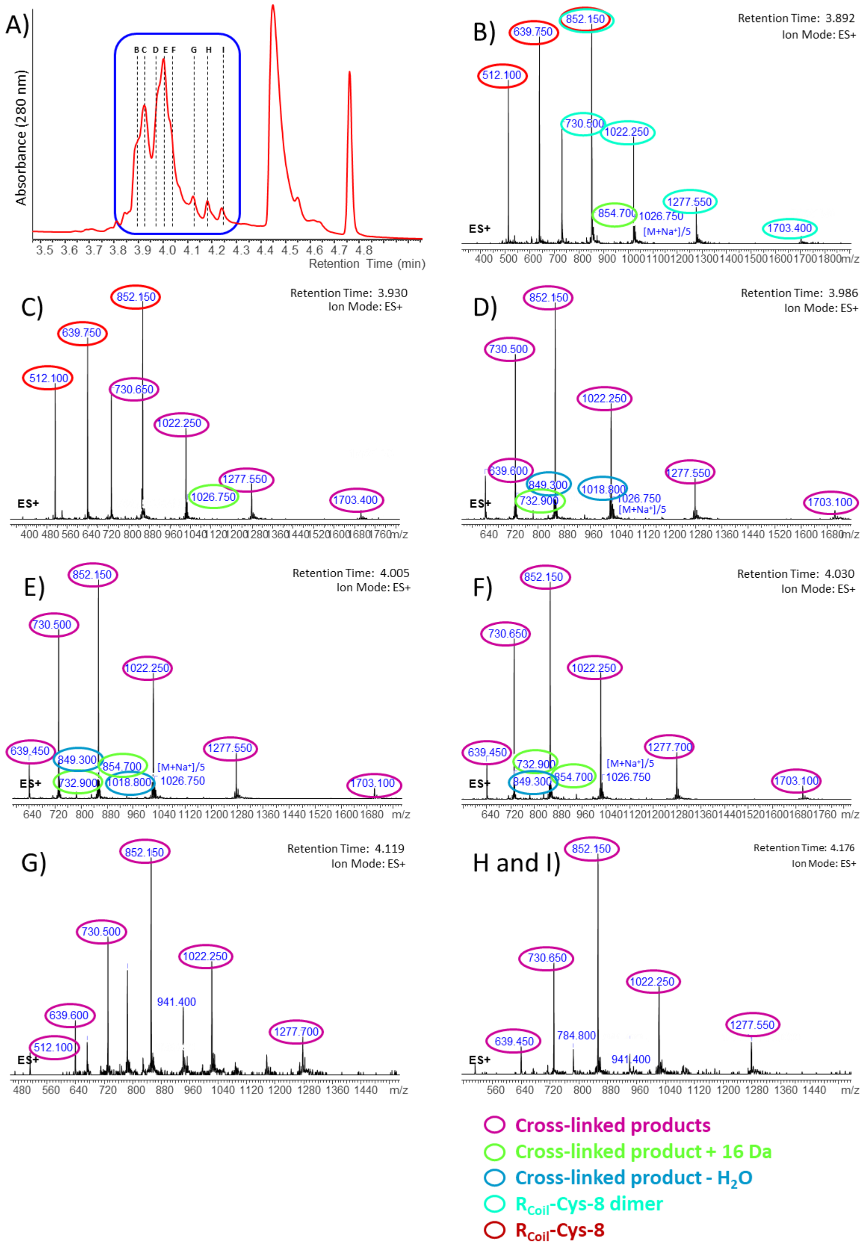

Supplementary Figure 50. A) HPLC-UV chromatogram recorded at 280 nm with the column Phenomenex Kinetex EVO C18 100 Å (150 x 4.6 mm, 5 µm, at 35 °C). B-I) ESI-MS spectrum of the peaks at 3.892, 3.930, 3.986, 4.005, 4.030, 4.119, and 4.176 minutes corresponding to the cross-linked products formed between R_{Coil}-Cys-8 and E_{Coil}-Dab_{Fur}-13 (blue rectangle in A). The red circles correspond to the mass-to-charge ratio of ions of R_{Coil}-Cys-8. The light blue circles correspond to the fragment ions of R_{Coil}-Cys-8 dimerization: $[M+3H]^{3+}/3$= 1703.4; $[M+4H]^{4+}/4$= 1277.5; $[M+5H]^{5+}/5$= 1022.2; $[M+6H]^{6+}/6$= 852.1; $[M+7H]^{7+}/7$= 730.5. The purple circles correspond to the mass-to-charge ratio of ions of the cross-linked product: $[M+3H]^{3+}/3$= 1703.1; $[M+4H]^{4+}/4$= 1277.5; $[M+5H]^{5+}/5$= 1022.2; $[M+6H]^{6+}/6$= 852.1; $[M+7H]^{7+}/7$= 730.6, $[M+7H]^{7+}/7$= 639.4. Exact mass (m/z) and molecular weight (MW) calculated for $C_{228}H_{383}N_{65}O_{65}S$ $[M+1H]^{+}$: 5103.8 Da and 5106.9; found 5106.9 Da. The blue circles correspond to the dehydrated (-18 Da) form of the cross-linked product, and the green circles correspond to the oxidated cross-linked product (+16 Da). Peaks not assigned correspond to degradation products.

8.7 Cross-linked product of R$_{Coil}$-Cys-8 with E$_{Coil}$-Dap$_{Fur}$-13

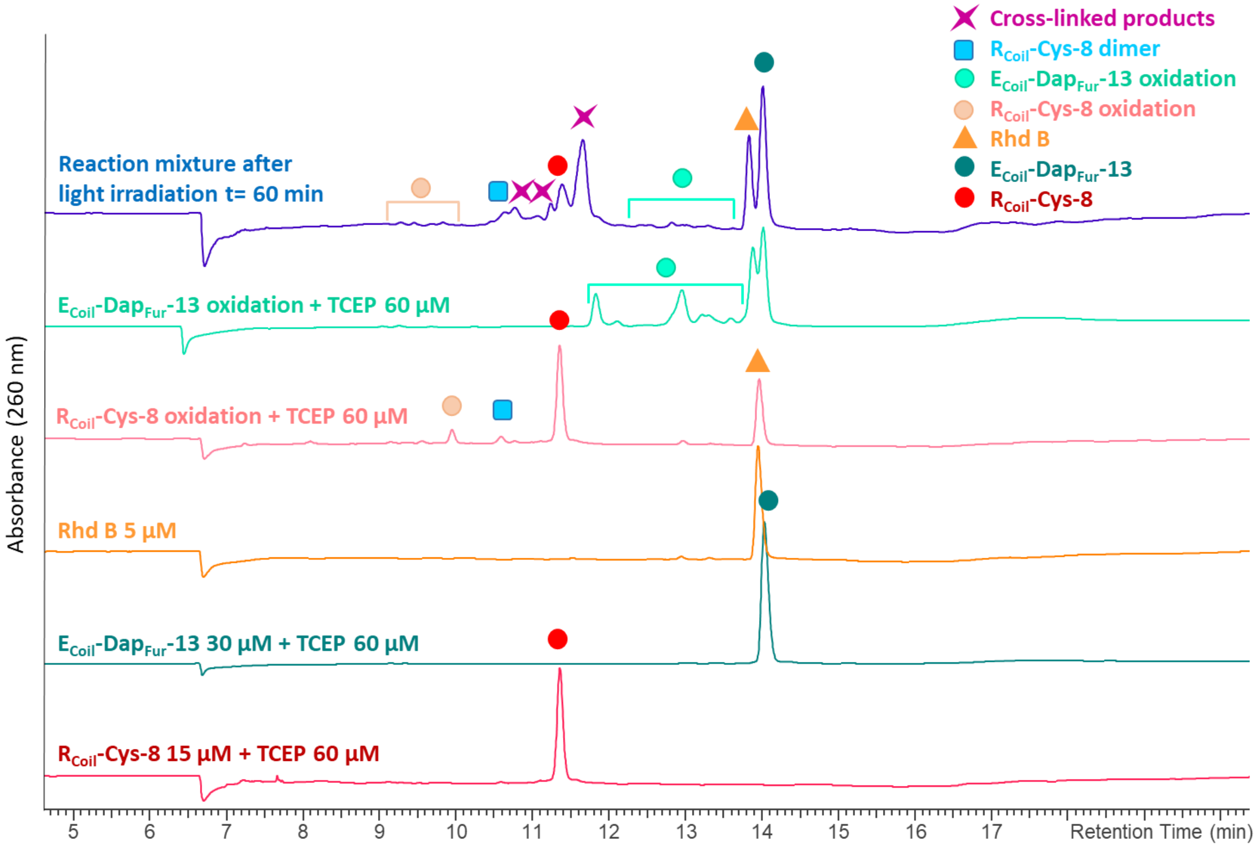

	Peak Area (Y units/ms)	*% XL area*
R$_{Coil}$-Cys-8 + R$_{Coil}$-Cys-8 dimer + R$_{Coil}$-Cys-8 oxidation	648267	
Cross-linked product	1621085	**71.4**
Total area	2269351	

Supplementary Figure 51. HPLC-UV chromatograms recorded at 260 nm with a XTerra® Shield RP18 column, 125Å (5µM 2,1 x 250mm). The reaction mixture after light irradiation (blue trace) is the cross-link reaction between R$_{Coil}$-Cys-8 + TCEP (red trace) and E$_{Coil}$-Dap$_{Fur}$-13 (dark green trace) after 60 minutes of light irradiation with Rhd B (orange trace) at 5 µM. The R$_{Coil}$-Cys-8 oxidation + TCEP (light red) trace and E$_{Coil}$-Dap$_{Fur}$-13 oxidation + TCEP (green) trace were generated by exposure to singlet oxygen generation by light irradiation in the presence of Rhd B at 5 µM for 60 minutes in absence of the other Coil. The cross-link yield was quantified as a percentage of the cross-linked product area and the values are indicated in the Table.

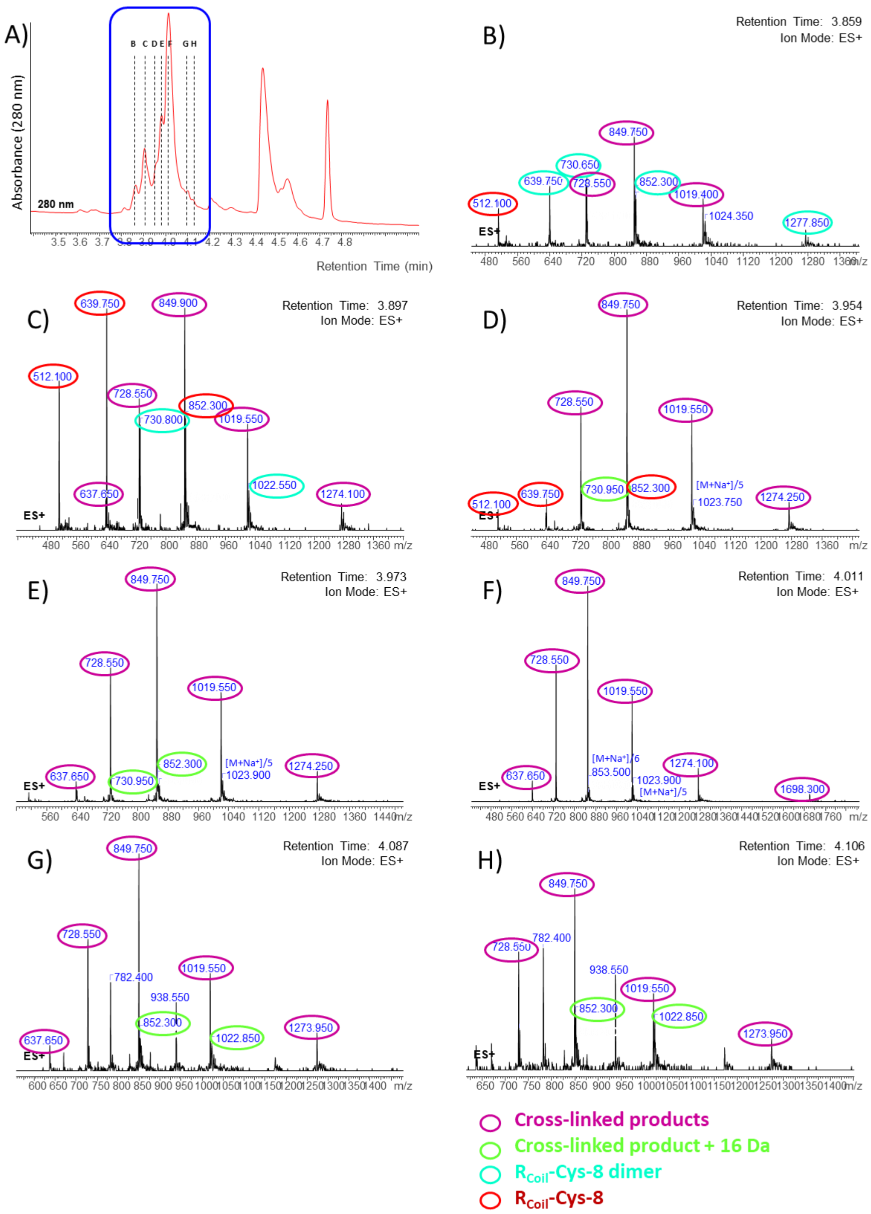

Supplementary Figure 52. A) HPLC-UV chromatogram recorded at 280 nm with the column Phenomenex Kinetex EVO C18 100 Å (150 x 4.6 mm, 5 µm, at 35 °C). B-I) ESI-MS spectrum of the peaks at 3.859, 3.897, 3.954, 3.973, 4.011, 4.087, and 4.106 minutes corresponding to the cross-linked products formed between R_{Coil}-Cys-8 and E_{Coil}-Dap_{Fur}-13 (blue rectangle in A). The red circles correspond to the mass-to-charge ratio of ions of R_{Coil}-Cys-8. The light blue circles correspond to the fragment ions of R_{Coil}-Cys-8 dimerization: $[M+4H]^{4+}/4$= 1277.8; $[M+5H]^{5+}/5$= 1022.5; $[M+6H]^{6+}/6$= 852.3; $[M+7H]^{7+}/7$= 730.6; $[M+8H]^{8+}/8$= 639.7. The purple circles correspond to the mass-to-charge ratio of ions of the cross-linked product: $[M+3H]^{3+}/3$= 1698.3; $[M+4H]^{4+}/4$= 1274.1; $[M+5H]^{5+}/5$= 1019.5; $[M+6H]^{6+}/6$= 849.7; $[M+7H]^{7+}/7$= 728.5, $[M+7H]^{7+}/7$= 637.6. Exact mass (m/z) and molecular weight (MW) calculated for $C_{227}H_{381}N_{65}O_{65}S$ $[M+1H]^{+}$: 5089.8 Da and 5092.9; found 5092.7 Da. The green circles correspond to the oxidated cross-linked product (+16 Da). Peaks not assigned correspond to degradation products.

9 Optimization of cross-linking conditions for Tyr

9.1 Cross-link between R_{Coil}-Tyr-8 and E_{Coil}-Lys_{Fur}-13

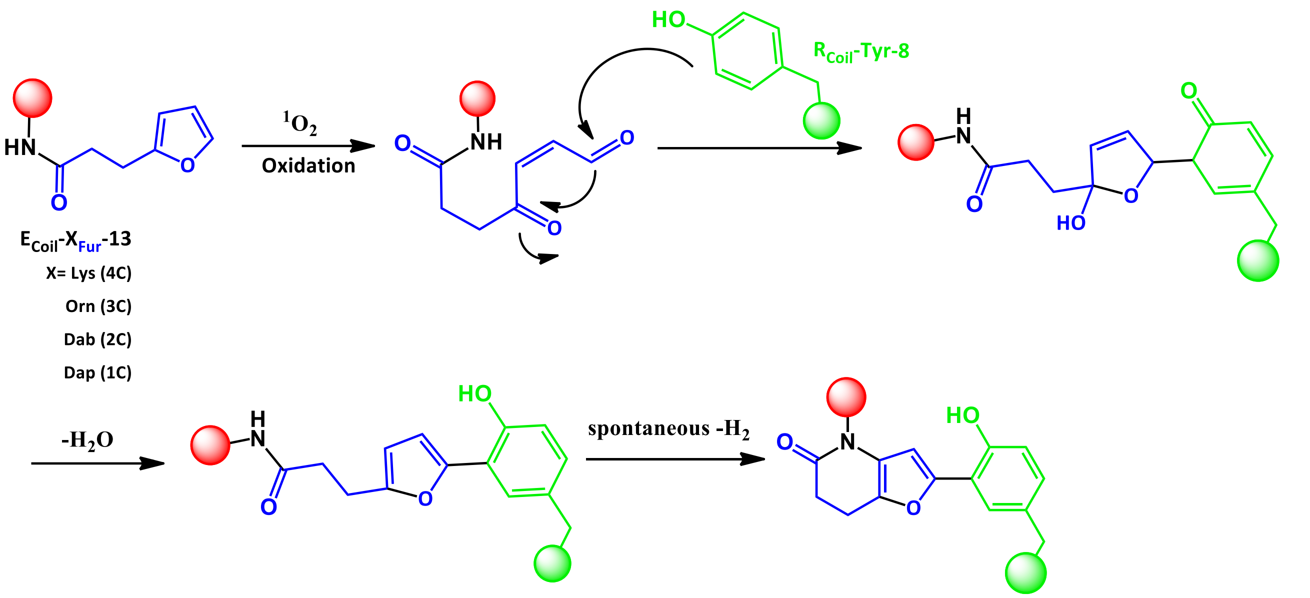

Supplementary Figure 53. A) Chemical structure of the plausible covalent bond that can be formed between R_{Coil}-Tyr-8 and E_{Coil}-Lys_{Fur}-13.

9.2 Quantitative analysis of the cross-link yield for Figure 8 in the manuscript

	Peak Area (Y units/ms)	% XL area
**Trace C**		
R_{Coil}-Tyr-8 + R_{Coil}-Tyr-8 oxidation	2758614	
**Cross-linked product**	968980	**26,0**
Total area	3727594	
**Trace B**		
R_{Coil}-Tyr-8 + R_{Coil}-Tyr-8 oxidation	1167478	
**Cross-linked product**	340810	**22,6**
Total area	1508287	
**Trace A**		
R_{Coil}-Tyr-8	1795120	
**Cross-linked product**	75112	**4,0**
Total area	1870233	

Table 5. The peak area was calculated integrating the peak in the HPLC chromatogram. The percentage of the cross-linked product (XL) area is the ratio between the XL area and the total area, multiplied by 100. The total area considered is the area of the R_{Coil}-Tyr-8 peak plus the area of the R_{Coil}-Tyr-8 oxidation peak plus the area of the cross-linked product.

9.3 Cross-linked product of R_{Coil}-Tyr-8 with E_{Coil}-Lys_{Fur}-13

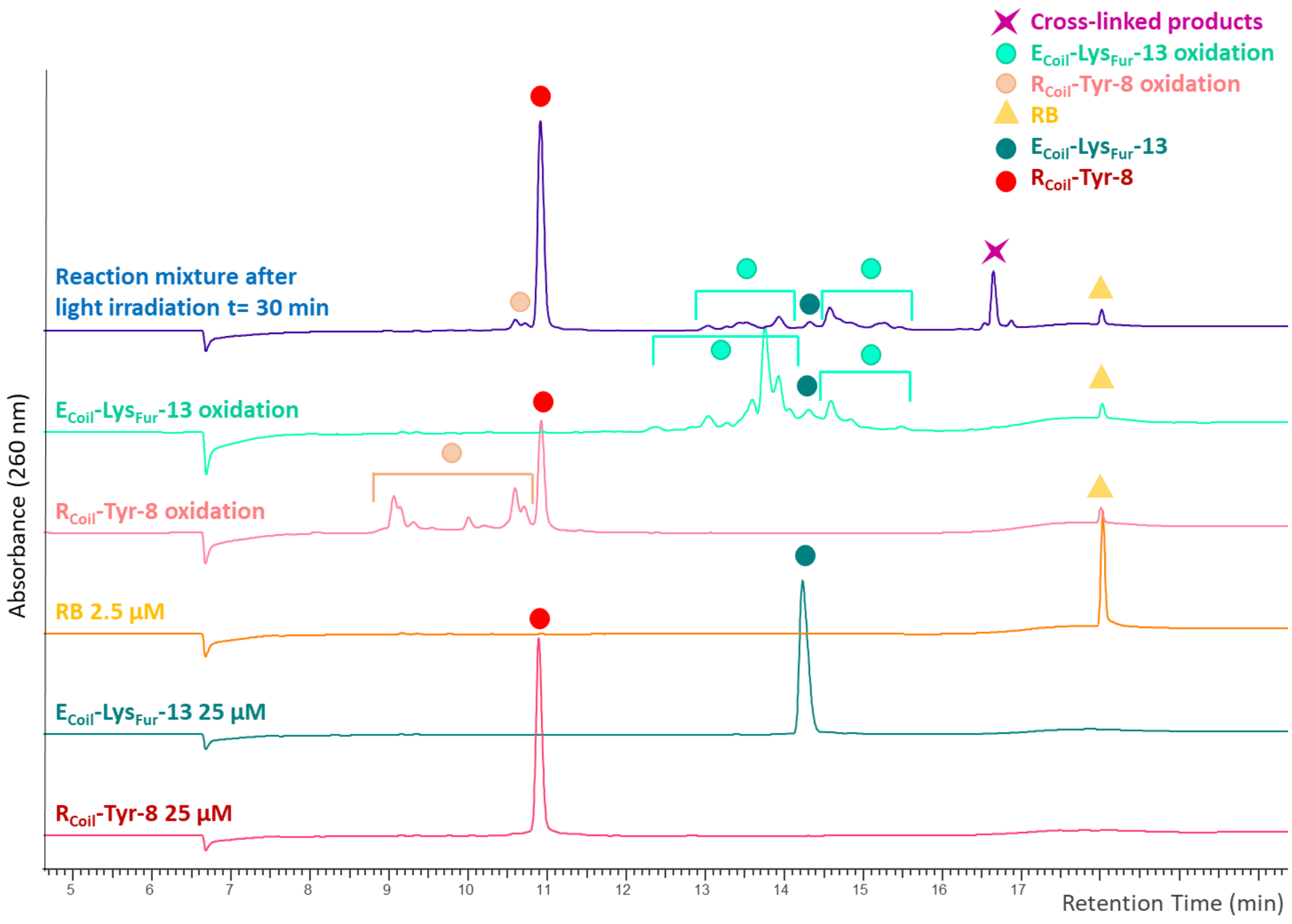

	Peak Area (Y units/ms)	*% XL area*
R_{Coil}-Tyr-8 + R_{Coil}-Tyr-8 oxidation	2765473	
Cross-linked product	756419	**21.5**
Total area	3521892	

Supplementary Figure 54. HPLC-UV chromatograms recorded at 260 nm with a XTerra® Shield RP18 column, 125Å (5µM 2,1 x 250mm). The reaction mixture after light irradiation (blue trace) is the cross-link reaction between R_{Coil}-Tyr-8 (red trace) and E_{Coil}-Lys_{Fur}-13 (dark green trace) after 30 minutes of light irradiation with RB (orange trace) at 2.5 µM. The R_{Coil}-Tyr-8 oxidation (light red) trace and E_{Coil}-Lys_{Fur}-13 oxidation (green) trace were generated by exposure to singlet oxygen by light irradiation in the presence of RB at 2.5 µM for 30 minutes in absence of the other Coil. The cross-link yield was quantified as a percentage of the cross-linked product area and the values are indicated in the Table.

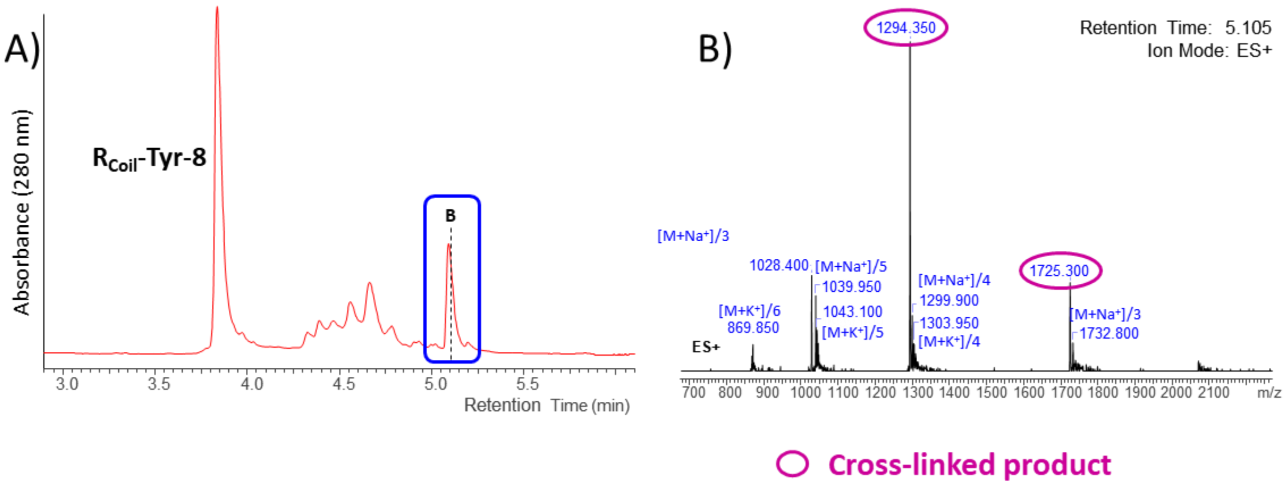

Supplementary Figure 55. A) HPLC-UV chromatogram recorded at 280 nm with the column Phenomenex Kinetex EVO C18 100 Å (150 x 4.6 mm, 5 µm, at 35 °C). B) ESI-MS spectrum of the peak at 5.105 minutes corresponding to the cross-linked product formed between R_{Coil}-Tyr-8 and E_{Coil}-Lys_{Fur}-13 (blue rectangle in A). The purple circles correspond to the mass-to-charge ratio of ions of the cross-linked product: $[M+3H]^{3+}/3$= 1725.3; $[M+4H]^{4+}/4$= 1294.34. Exact mass (m/z) and molecular weight (MW) calculated for $C_{236}H_{385}N_{65}O_{65}$ $[M+1H]^{+}$: 5169.9 Da and 5173.0; found 5173.3 Da. Peak not assigned correspond to degradation product.

9.4 Cross-linked product of R_{Coil}- Tyr-8 with E_{Coil}-Orn_{Fur}-13

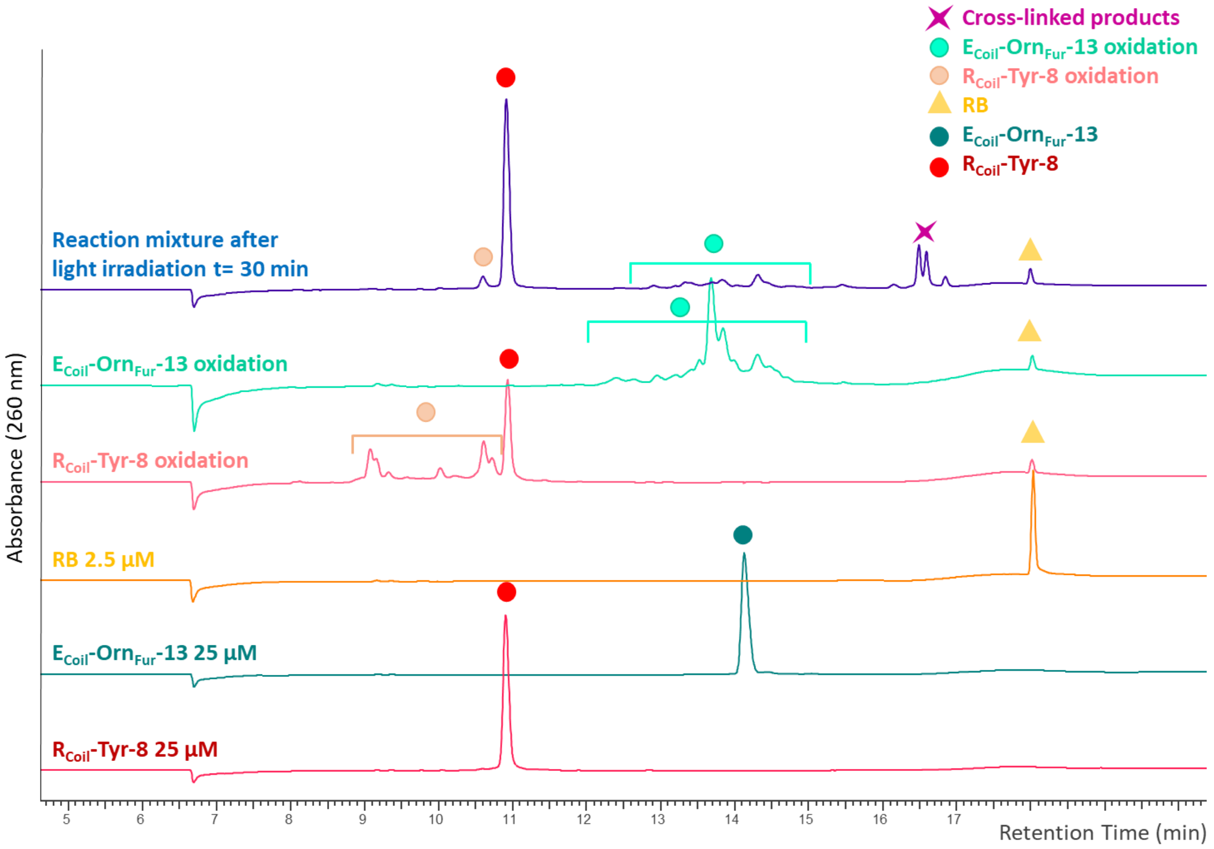

	Peak Area (Y units/ms)	*% XL area*
R_{Coil}-Tyr-8 + R_{Coil}-Tyr-8 oxidation	2758614	
Cross-linked product	968.980	**26.0**
Total area	3727594	

Supplementary Figure 56. HPLC-UV chromatograms recorded at 260 nm with a XTerra® Shield RP18 column, 125Å (5µM 2,1 x 250mm). The reaction mixture after light irradiation (blue trace) is the cross-link reaction between R_{Coil}-Tyr-8 (red trace) and E_{Coil}-Orn_{Fur}-13 (dark green trace) after 30 minutes of light irradiation with RB (orange trace) at 2.5 µM. The R_{Coil}-Tyr-8 oxidation (light red) trace and E_{Coil}-Orn_{Fur}-13 oxidation (green) trace were generated by exposure to singlet oxygen by light irradiation in the presence of RB at 2.5 µM for 30 minutes in absence of the other Coil. The cross-link yield was quantified as a percentage of the cross-linked product area and the values are indicated in the Table.

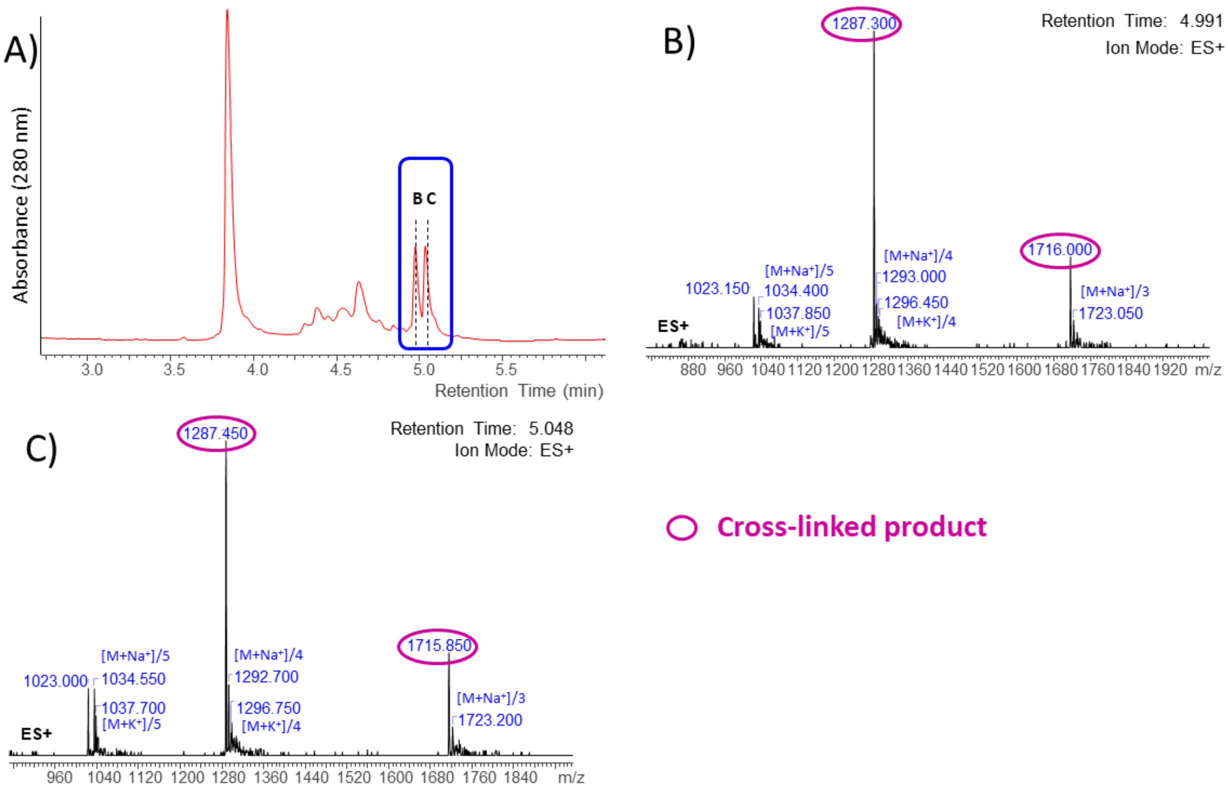

Supplementary Figure 57. A) HPLC-UV chromatogram recorded at 280 nm with the column Phenomenex Kinetex EVO C18 100 Å (150 x 4.6 mm, 5 µm, at 35 °C). B-C) ESI-MS spectrum of the peak at 4.991 and 5.048 minutes corresponding to the cross-linked product formed between R_{Coil}-Tyr-8 and E_{Coil}-Orn_{Fur}-13 (blue rectangle in A). The purple circles correspond to the mass-to-charge ratio of ions of the cross-linked product: $[M+3H]^{3+}/3$= 1716.0; $[M+4H]^{4+}/4$= 1287.3. Peak not assigned correspond to degradation product.

9.5 Cross-linked product of R$_{Coil}$- Tyr-8 with E$_{Coil}$-Dab$_{Fur}$-13

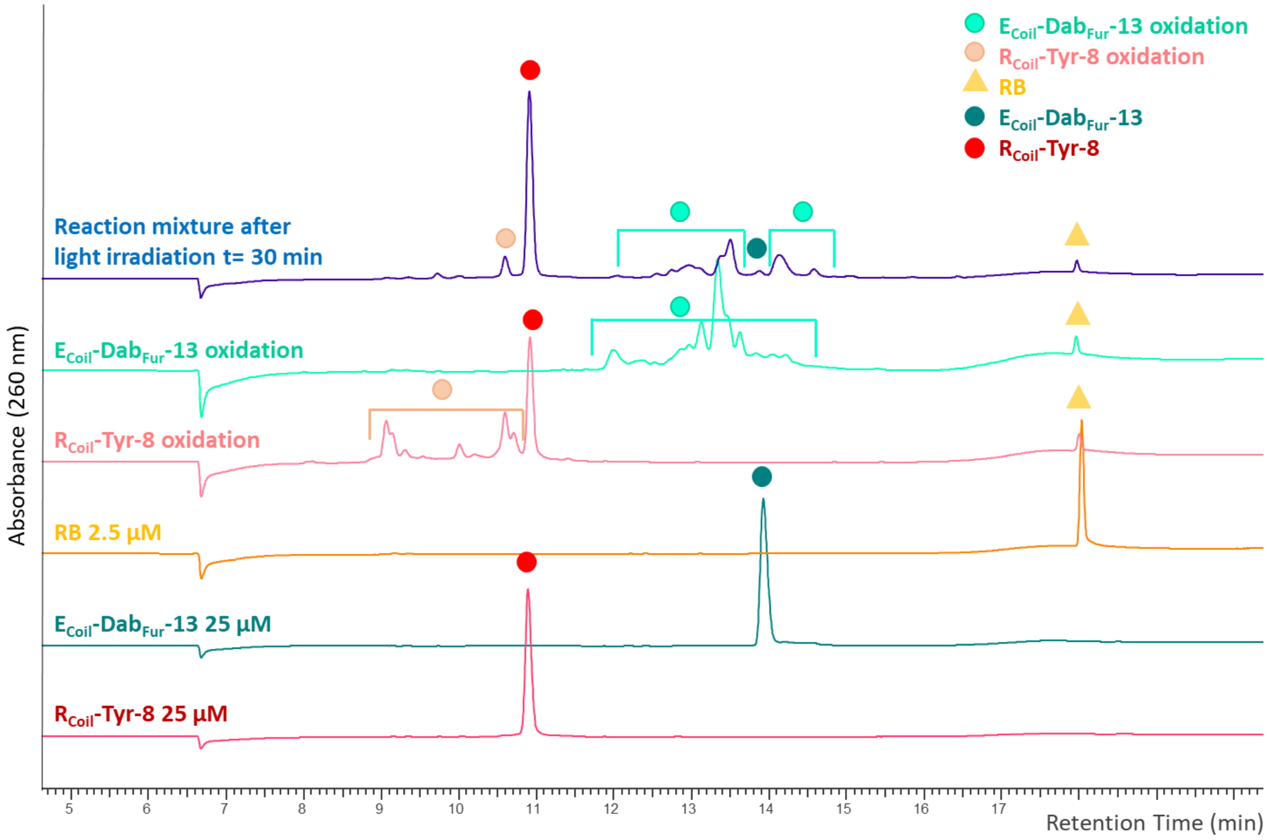

Supplementary Figure 58. HPLC-UV chromatograms recorded at 260 nm with a XTerra® Shield RP18 column, 125Å (5µM 2,1 x 250mm). The reaction mixture after light irradiation (blue trace) is the cross-link reaction between R$_{Coil}$-Tyr-8 (red trace) and E$_{Coil}$-Dab$_{Fur}$-13 (dark green trace) after 30 minutes of light irradiation with RB (orange trace) at 2.5 µM. The R$_{Coil}$-Tyr-8 oxidation (light red) trace and E$_{Coil}$-Dab$_{Fur}$-13 oxidation (green) trace were generated by exposure to singlet oxygen by light irradiation in the presence of RB at 2.5 µM for 30 minutes in absence of the other Coil.

9.6 Cross-linked product of R_{Coil}- Tyr-8 with E_{Coil}-Dap_{Fur}-13

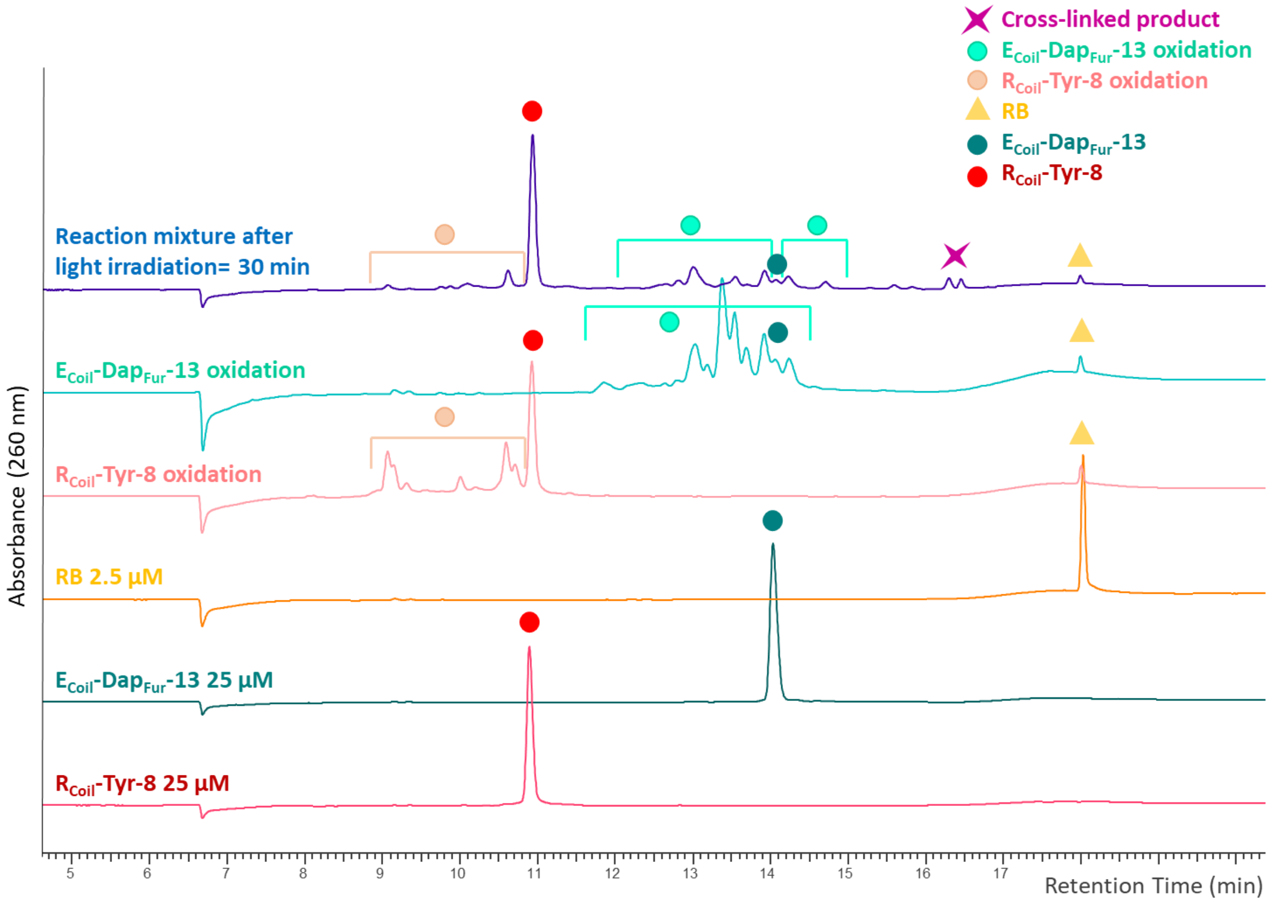

	Peak Area (Y units/ms)	*% XL area*
R_{Coil}-Tyr-8 + R_{Coil}-Tyr-8 oxidation	2810249	
Cross-linked product	222312	**7.3**
Total area	3032561	

Supplementary Figure 59. HPLC-UV chromatograms recorded at 260 nm with a XTerra® Shield RP18 column, 125Å (5µM 2,1 x 250mm). The reaction mixture after light irradiation (blue trace) is the cross-link reaction between R_{Coil}-Tyr-8 (red trace) and E_{Coil}-Dap_{Fur}-13 (dark green trace) after 30 minutes of light irradiation with RB (orange trace) at 2.5 µM. The R_{Coil}-Tyr-8 oxidation (light red) trace and E_{Coil}-Dap_{Fur}-13 oxidation (green) trace were generated by exposure to singlet oxygen by light irradiation in the presence of RB at 2.5 µM for 30 minutes in absence of the other Coil. The cross-link yield was quantified as a percentage of the cross-linked product area and the values are indicated in the Table.

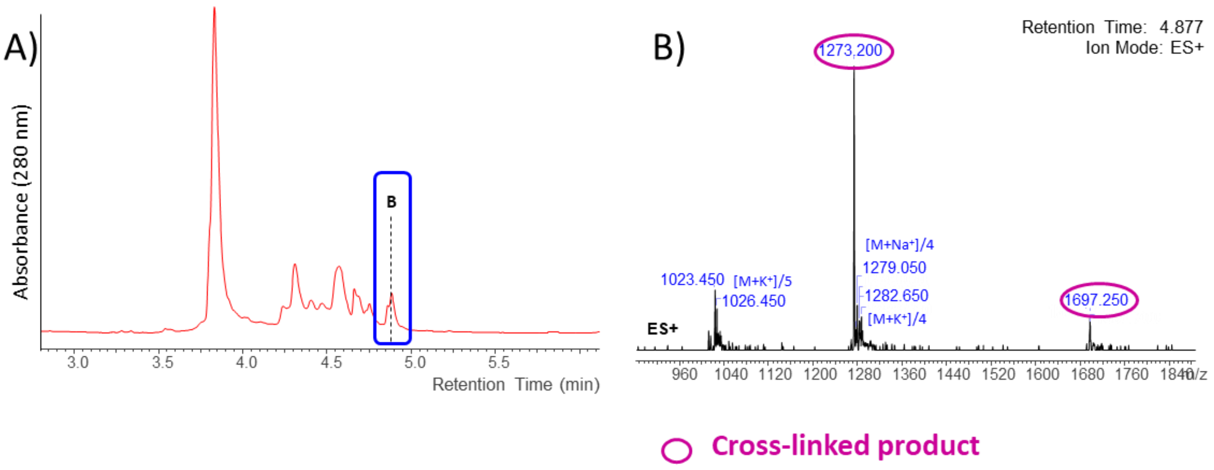

Supplementary Figure 60. A) HPLC-UV chromatogram recorded at 280 nm with the column Phenomenex Kinetex EVO C18 100 Å (150 x 4.6 mm, 5 µm, at 35 °C). B) ESI-MS spectrum of the peak at 4.877 minutes corresponding to the cross-linked product formed between R_{Coil}-Tyr-8 and E_{Coil}-Dap_{Fur}-13 (blue rectangle in A). The purple circles correspond to the mass-to-charge ratio of ions of the cross-linked product: $[M+3H]^{3+}/3$= 1697.3; $[M+4H]^{4+}/4$= 1273.2. Peak not assigned correspond to degradation product.

10 Optimization of cross-linking conditions for Trp

10.1 Cross-linked product of R_{Coil}-Trp-8 with E_{Coil}-Lys_{Fur}-13

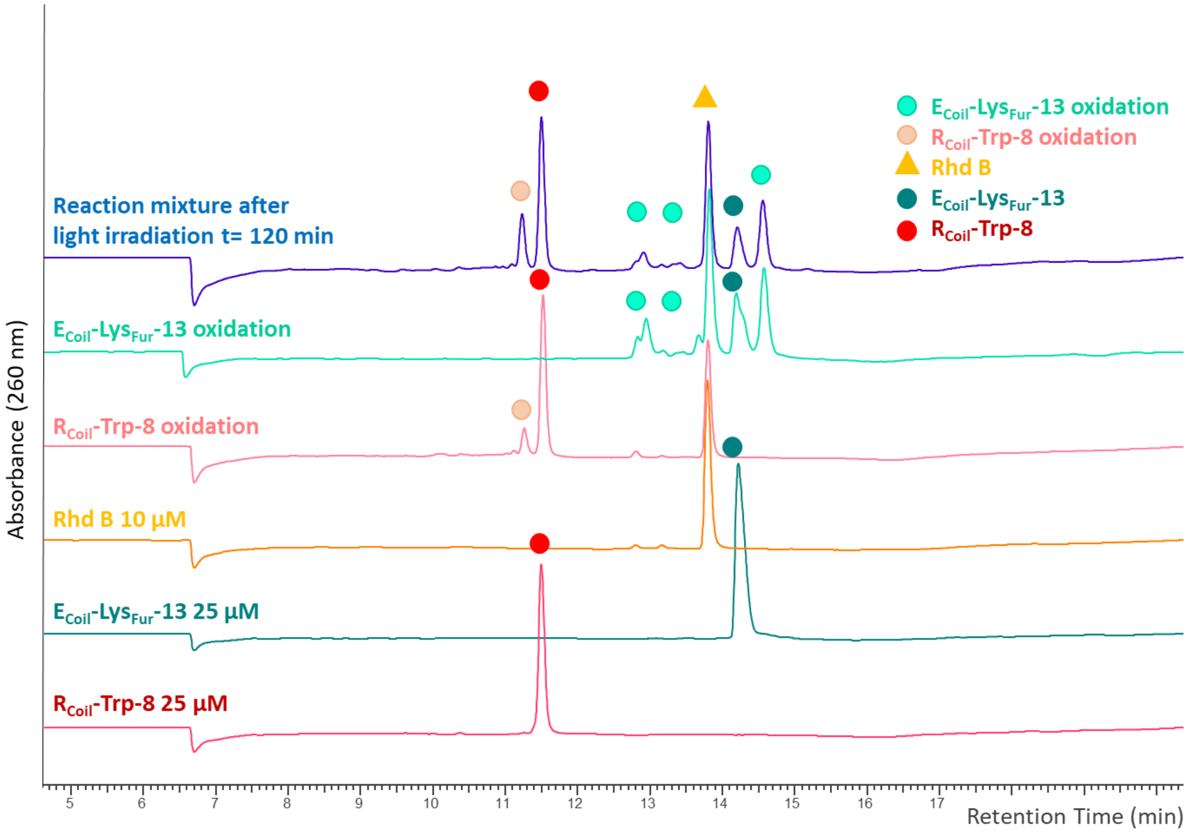

Supplementary Figure 61. HPLC-UV chromatograms recorded at 260 nm with a XTerra® Shield RP18 column, 125Å (5µM 2,1 x 250mm). The reaction mixture after light irradiation (blue trace) is the cross-link reaction between R_{Coil}-Trp-8 (red trace) and E_{Coil}-Lys_{Fur}-13 (dark green trace) after 120 minutes of light irradiation with Rhd B (orange trace) at 10 µM. The R_{Coil}-Trp-8 oxidation (light red) trace and E_{Coil}-Lys_{Fur}-13 oxidation (green) trace were generated by exposure to singlet oxygen by light irradiation in the presence of Rhd B at 10 µM for 120 minutes in absence of the other Coil.

10.2 Cross-linked product of R$_{Coil}$- Trp-8 with E$_{Coil}$-Orn$_{Fur}$-13

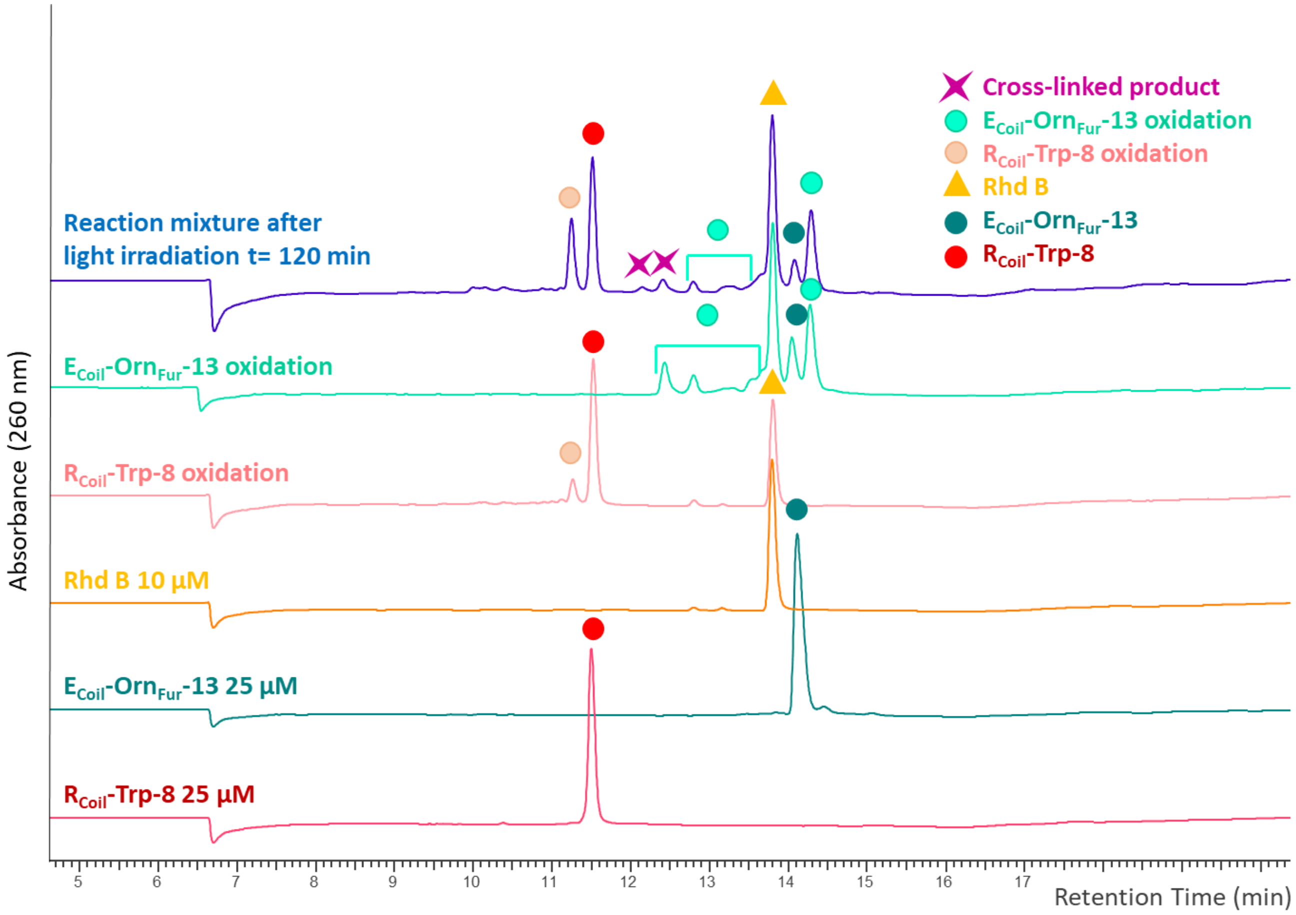

	Peak Area (Y units/ms)	*% XL area*
R$_{Coil}$-Trp-8 + R$_{Coil}$-Trp-8 oxidation	1805474	
Cross-linked product	205532	**10.2**
Total area	2011006	

Supplementary Figure 62. HPLC-UV chromatograms recorded at 260 nm with a XTerra® Shield RP18 column, 125Å (5µM 2,1 x 250mm). The reaction mixture after light irradiation (blue trace) is the cross-link reaction between R$_{Coil}$-Trp-8 (red trace) and E$_{Coil}$-Orn$_{Fur}$-13 (dark green trace) after 120 minutes of light irradiation with Rhd B (orange trace) at 10 µM. The R$_{Coil}$-Trp-8 oxidation (light red) trace and E$_{Coil}$-Orn$_{Fur}$-13 oxidation (green) trace were generated by exposure to singlet oxygen by light irradiation in the presence of Rhd B at 10 µM for 120 minutes in absence of the other Coil. The cross-link yield was quantified as a percentage of the cross-linked product area and the values are indicated in the Table.

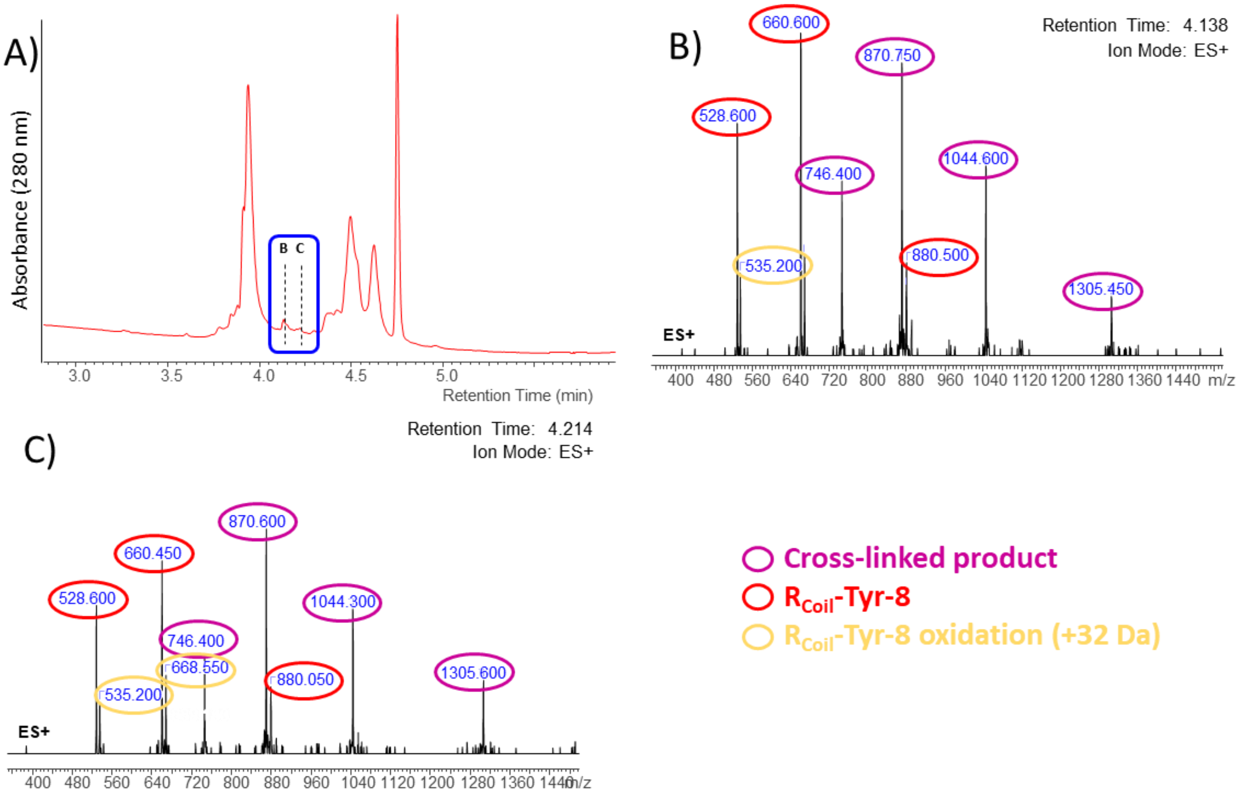

Supplementary Figure 63. A) HPLC-UV chromatogram recorded at 280 nm with the column Phenomenex Kinetex EVO C18 100 Å (150 x 4.6 mm, 5 µm, at 35 °C). B-C) ESI-MS spectrum of the peaks at 4.138 and 4.214 minutes corresponding to the cross-linked products formed between R_{Coil}-Trp-8 and E_{Coil}-Orn_{Fur}-13 (blue rectangle in A). The purple circles correspond to the mass-to-charge ratio of ions of the cross-linked product: $[M+4H]^{4+}/4$= 1305.5; $[M+5H]^{5+}/5$= 1044.6; $[M+6H]^{6+}/6$= 870.6; $[M+7H]^{7+}/7$= 746.4. Found mass: 5218.02 Da. The red circles correspond to the mass-to-charge ratio of ions of R_{Coil}-Trp-8. The yellow circles correspond to the mass-to-charge ratio of ions of the oxidated R_{Coil}-Trp-8 (+32 Da).

10.3 Cross-linked product of R_{Coil}- Trp-8 with E_{Coil}-Dab_{Fur}-13

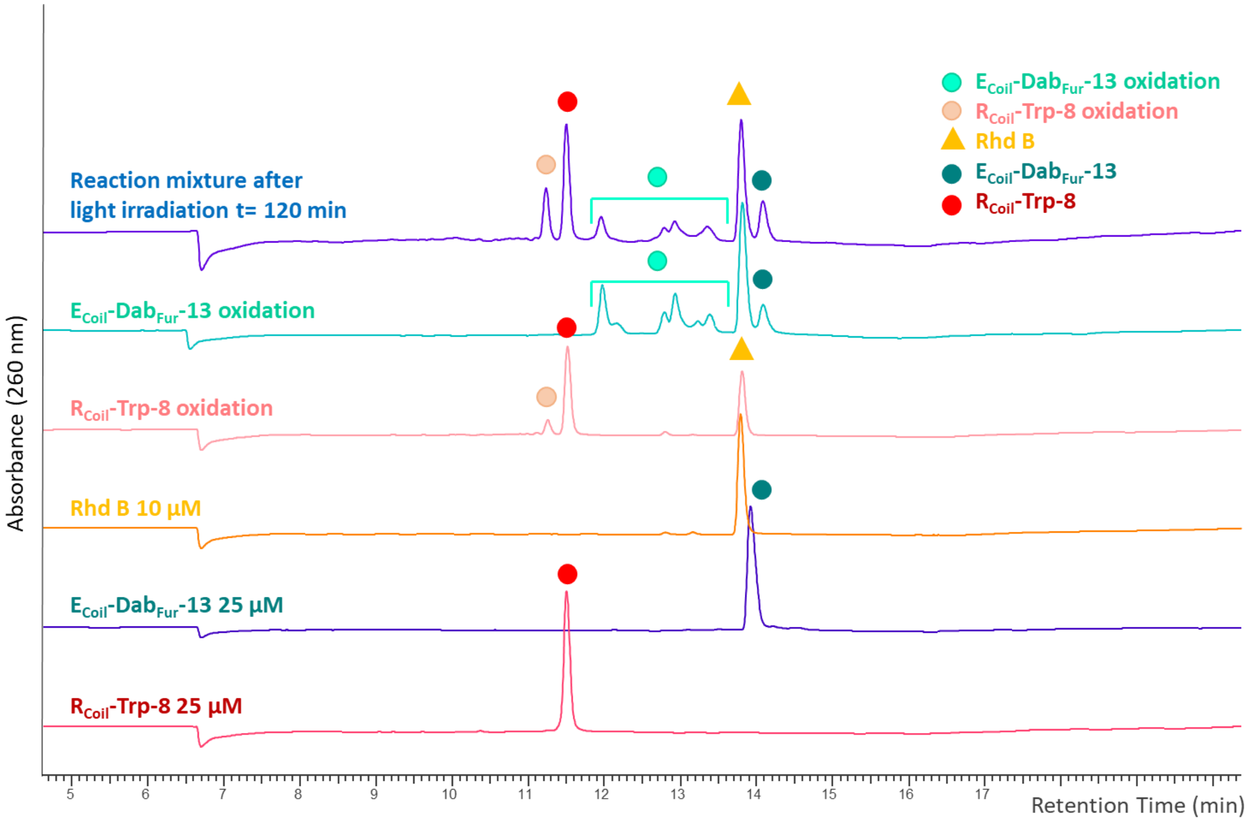

Supplementary Figure 64. HPLC-UV chromatograms recorded at 260 nm with a XTerra® Shield RP18 column, 125Å (5µM 2,1 x 250mm). The reaction mixture after light irradiation (blue trace) is the cross-link reaction between R_{Coil}-Trp-8 (red trace) and E_{Coil}-Dab_{Fur}-13 (dark green trace) after 120 minutes of light irradiation with Rhd B (orange trace) at 10 µM. The R_{Coil}-Trp-8 oxidation (light red) trace and E_{Coil}-Dab_{Fur}-13 oxidation (green) trace were generated by exposure to singlet oxygen by light irradiation in the presence of Rhd B at 10 µM for 120 minutes in absence of the other Coil.

11 REFERENCES

[1] K. Katoh, and D.M. Standley, MAFFT multiple sequence alignment software version 7: improvements in performance and usability. Mol Biol Evol 30 (2013) 772-80.

[2] A.M. Waterhouse, J.B. Procter, D.M. Martin, M. Clamp, and G.J. Barton, Jalview Version 2--a multiple sequence alignment editor and analysis workbench. Bioinformatics 25 (2009) 1189-91.

[3] B. Webb, and A. Sali, Comparative Protein Structure Modeling Using MODELLER. Curr Protoc Bioinformatics 54 (2016) 5 6 1-5 6 37.

[4] R.A. Laskowski, M.W. Macarthur, D.S. Moss, and J.M. Thornton, Procheck - a Program to Check the Stereochemical Quality of Protein Structures. J Appl Crystallogr 26 (1993) 283-291.

[5] D.A.e.a. Case, AMBER 2016. University of California, San Francisco (2016).

[6] J. Diharce, M. Cueto, M. Beltramo, V. Aucagne, and P. Bonnet, In Silico Peptide Ligation: Iterative Residue Docking and Linking as a New Approach to Predict Protein-Peptide Interactions. Molecules 24 (2019).

[7] J.R. Litowski, and R.S. Hodges, Designing heterodimeric two-stranded alpha-helical coiled-coils. Effects of hydrophobicity and alpha-helical propensity on protein folding, stability, and specificity. J Biol Chem 277 (2002) 37272-9.
